# Extracting Diffusive States of Rho GTPase in Live Cells: Towards *In Vivo* Biochemistry

**DOI:** 10.1371/journal.pcbi.1004297

**Published:** 2015-10-29

**Authors:** Peter K. Koo, Matthew Weitzman, Chandran R. Sabanaygam, Kenneth L. van Golen, Simon G. J. Mochrie

**Affiliations:** 1 Department of Physics, Yale University, New Haven, Connecticut, United States of America; 2 Department of Biological Sciences, University of Delaware, Newark, Delaware, United States of America; 3 Delaware Biotechnology Institute, Bioimaging Center, Newark, Delaware, United States of America; 4 Department of Applied Physics, Yale University, New Haven, Connecticut, United States of America; ISRAEL

## Abstract

Resolving distinct biochemical interaction states when analyzing the trajectories of diffusing proteins in live cells on an individual basis remains challenging because of the limited statistics provided by the relatively short trajectories available experimentally. Here, we introduce a novel, machine-learning based classification methodology, which we call perturbation expectation-maximization (pEM), that simultaneously analyzes a population of protein trajectories to uncover the system of diffusive behaviors which collectively result from distinct biochemical interactions. We validate the performance of pEM *in silico* and demonstrate that pEM is capable of uncovering the proper number of underlying diffusive states with an accurate characterization of their diffusion properties. We then apply pEM to experimental protein trajectories of Rho GTPases, an integral regulator of cytoskeletal dynamics and cellular homeostasis, *in vivo* via single particle tracking photo-activated localization microcopy. Remarkably, pEM uncovers 6 distinct diffusive states conserved across various Rho GTPase family members. The variability across family members in the propensities for each diffusive state reveals non-redundant roles in the activation states of RhoA and RhoC. In a resting cell, our results support a model where RhoA is constantly cycling between activation states, with an imbalance of rates favoring an inactive state. RhoC, on the other hand, remains predominantly inactive.

## Author Summary

Single particle tracking is a powerful tool that captures the diffusive dynamics of proteins as they undergo various interactions in living cells. Uncovering different biochemical interactions by analyzing the diffusive behaviors of individual protein trajectories, however, is challenging due to the limited statistics provided by short trajectories and experimental noise sources which are intimately coupled into each protein’s localization. Here, we introduce a novel, unsupervised, machine-learning based classification methodology, which we call perturbation expectation-maximization (pEM), that simultaneously analyzes a population of protein trajectories to uncover the system of diffusive behaviors which collectively result from distinct biochemical interactions. We validate the performance of pEM *in silico* and *in vivo* on the biological system of Rho GTPase, a signal transduction protein responsible for regulating cytoskeletal dynamics. We envision that the presented methodology will be applicable to a wide range of single protein tracking data where different biochemical interactions result in distinct diffusive behaviors. More generally, this study brings us an important step closer to the possibility of monitoring the endogenous biochemistry of diffusing proteins within live cells with single molecule resolution.

This is a *PLOS Computational Biology* Methods paper

## Introduction

As critical regulators of cytoskeletal dynamics, Rho family GTPases play numerous roles in normal biological processes and their dysregulation drives the progression of multiple pathological conditions, notably cancer [1–4]. The Rho subfamily consists of RhoA and RhoC, which are 85% identical in sequence, with much of their sequence divergence occurring in the C-terminal hypervariable domain [5]. Despite such high sequence homology, several reports indicate that RhoA and RhoC are not redundant [6–13]. For instance, RhoA is often inhibitory while RhoC facilitates cancer cell invasion [8–12]. Moreover, while RhoA is able to transform mouse fibroblasts in culture and enhance tumor formation by these cells in mice, RhoC is unable to do so [13].

**Fig 1:**
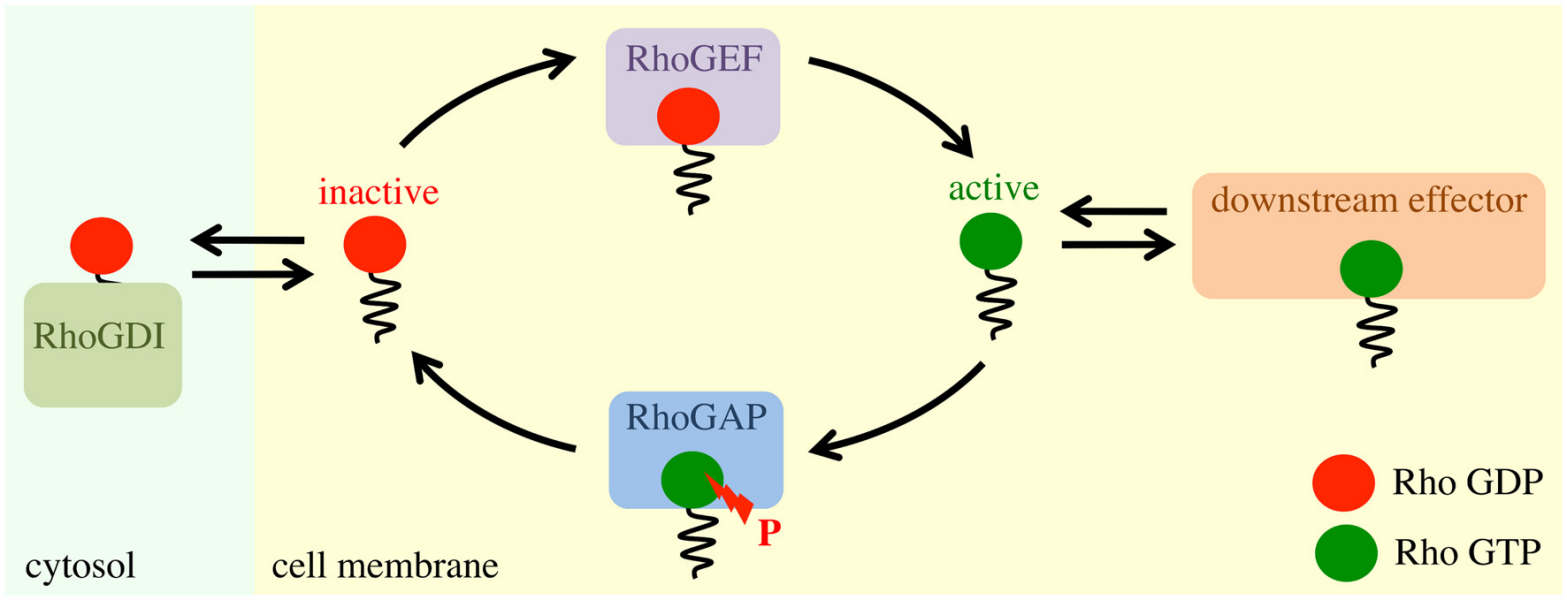
Diagram of Rho activation model. Rho can be found sequestered in the cytosol through association with RhoGDI. Otherwise, when not bound to RhoGDI, Rho is associated with the cell membrane. When diffusing on the membrane, RhoGEFs catalyze the exchange of GDP with GTP. Active GTP bound Rho freely diffuses as a single protein and associates with numerous downstream effectors as well as macromolecular complexes. Rho signaling is terminated by binding to RhoGAP, which facilitates the hydrolysis of GTP. The freely diffusing inactive RhoA may then reenter the cycle or be sequestered to the cytosol by RhoGDI.

A simplified model, which captures the main components of Rho GTPases’ activation cycle, is displayed in Fig 1. Rho guanine dissociation inhibitors (RhoGDIs) sequester Rho proteins in the cytoplasm, curtailing its activity. Numerous signaling cascades, however, lead to dissociation of this complex, driving Rho to associate with the plasma membrane. On the membrane, Rho becomes activated when the GDP is exchanged for GTP. This process is catalyzed by Rho guanine nucleotide exchange factors (RhoGEFs). In its active form, Rho may perform its function with various downstream effectors. The activation cycle is completed with the hydrolysis of GTP which is catalyzed by Rho GTPase activating proteins (RhoGAPs), returning Rho back to an inactive GDP form. In this model, Rho is constantly cycling between activation states [14–16]. RhoGEFs and RhoGAPs work in an antagonistic fashion to regulate the activation of Rho. The net balance of these activities favors the GDP-bound form in the resting state of the cell. Exogenous stimulation may then shift the equilibrium of nucleotide states towards an increase in the GTP-bound form through concomitant activation of GEFs and inhibition of GAPs [16]. How these interactions manifest inside live cells, however, remain poorly understood.

Recent advances in live cell imaging have now made it possible to non-invasively monitor the motions of individual proteins inside living cells [17]. Specifically, single particle tracking photoactivated localization microscopy (sptPALM) stochastically photoconverts a subset of fluorescently labeled proteins within a high population density, facilitating the unambiguous tracking of individual proteins simultaneously. Each resultant protein trajectory contains information on the protein’s interactions with the local environment, which includes the material properties of the diffusing medium, presence of a net flow, and spatial confinement. In addition, the underlying diffusivity may change when the protein is associated to other proteins or when it undergoes a conformational change, which alters its radius of gyration. In essence, the diffusion of a protein can act as a reporter, not only of the protein’s local environment, but also of the protein’s biochemical state. In this context, it is intriguing to consider the possibility of being able to reconstruct Rho’s interactions and biochemistry, and thus its biological roles, using the membrane-associated dynamics of individual Rho proteins inside live cells.

Quantitatively characterizing the information provided by single protein trajectories acquired via sptPALM, however, is challenging due to the limited statistics arising from the photoinstability of the fluorescent probes, which result in a premature truncation of the observed protein trajectories [18]. In addition, experimental noise sources obscure the true instantaneous positions of each diffusing protein via “static” [19–21] and “dynamic” localization noise [19]. Static localization noise results from the limited number of photons emitted by a fluorophore within the camera’s exposure time. Dynamic localization noise results from the protein’s diffusion during the camera’s exposure time, which in turn, creates motion blur. The resultant short, noisy protein trajectories demand statistically-correct analyses that properly account for these experimental noise sources so that the underlying protein motions—and hence the underlying biology—can be characterized as accurately as possible.

Due to the limited statistics of short protein trajectories, characterization of the long-time diffusive behavior, which manifests interactions with the environment leading to confinement, flow, or anomalous diffusion, remains challenging (S1 Text). Thus, it is common to focus on the short-time diffusive dynamics, which describe the protein’s micro-diffusion prior to these interactions, which presumably occur on longer time-scales [22–24]. In theory, short-time diffusion analysis allows for the determination of biochemical interaction states due to protein-protein and protein-lipid interactions. In practice, the short-time diffusivity is traditionally quantified by fitting the first few time lags of the mean squared displacement (MSD) from an individual protein trajectory [17, 25–29]. However, due to the stochastic nature of diffusion, the resulting distribution of short-time diffusion coefficients from a collection of protein trajectories tends to be difficult to interpret, especially when the track lengths are short (S1 Text). Moreover, protein species that collectively exhibit heterogeneous diffusive behaviors arising from different biochemical interaction states, as would be expected for Rho proteins, yield an even more complex distribution comprised of a mixture of component distributions each generated by a distinct diffusive state.

The simplest strategy for resolving the diffusive states within a collection of heterogeneous protein trajectories is to apply a least squares fit to the cumulative distribution of the population of protein track displacements with a multi-component cumulative distribution function (CDF), in which each component represents a set of Gaussian-distributed displacements [30]—a Gaussian being the propagator for normal diffusion [31]. Alternatively, variational Bayes single particle tracking (vbSPT) has been recently introduced to uncover the number and properties of normal diffusive states and their corresponding transition kinetics between each state [32]. CDF and vbSPT, however, make an implicit assumption that each displacement of the protein trajectory is independent. Even though this assumption is theoretically consistent with the Markovian nature of normal diffusion [33], experimental protein track displacements are, in fact, not Markovian as a result of correlations between nearest-neighbor displacements, which inevitably arise as a result of static and dynamic localization noise [34].

Here, we introduce and expound a novel machine-learning-based classification methodology, which we term perturbation expectation-maximization (pEM), that simultaneously analyzes a population of protein trajectories, accounting for the experimental correlations in a statistically-correct fashion, to uncover the system of biochemical interactions which collectively result in distinct diffusive behaviors, each defined by an underlying diffusivity and static localization noise. We first validate the performance of pEM against various sets of synthetic protein trajectories containing static and dynamic localization noise under realistic conditions. We illustrate how pEM performance depends on the number of protein trajectories and compare its performance with CDF and vbSPT. We then apply pEM to experimental protein trajectories of Rho GTPase in live cells acquired via sptPALM.

Remarkably, our pEM methodology uncovers six normal diffusive states, each with a diffusivity and static localization noise, that appear conserved across RhoA and RhoC, and their various functional mutants. These Rho GTPase family members, however, yield significant variability in their propensity for each of the diffusive states, revealing non-redundant roles for RhoA and RhoC. In a minimally stimulated cell, we find that activation of RhoA alone does not drive binding to large macromolecular complexes, whereas RhoC activation results in a significant rise in population into these complexes. In an unstimulated cell, our results support a model where RhoA constantly cycles between activation states with an imbalance in transition rates favoring inactive states. RhoC, on the other hand, remains predominantly inactive.

The pEM methodology introduced in this paper will be broadly applicable to a variety of biological systems, whenever different biochemical interactions manifest as distinct diffusive behaviors. More generally, pEM brings us an important step closer to the possibility of monitoring the endogenous biochemistry of diffusing proteins within live cells with single molecule resolution.

## Materials and Methods

### Maximum likelihood framework for a collection of diffusive states

Our classification procedure to uncover the system of biochemical interactions is based on determining the diffusive state of each interaction. Specifically, we employ a systems-level likelihood function, given by a Gaussian mixture model (GMM) of multivariate Gaussians, to account for the different diffusion coefficients and static localization noises which collectively result from different biochemical interactions experienced across a population of protein trajectories.

For a collection of *M* one-dimensional (1D) protein track displacements undergoing normal diffusion with *K* underlying diffusive states, the systems-level likelihood function, or, equivalently, the log-likelihood function is given by (see S2 Text for derivation):

$$\text{ln}\;ℒ(\Delta \hat{x}|\hat{\pi },\hat{\Sigma })=\sum_{m=1}^{M}\text{ln}\;\left\{\sum_{k=1}^{K}\pi_{k}P(\Delta x_{m}|\Sigma_{k})\right\},$$ (1)

where Δ**x**_*m*_ represents the vector of *N*_*m*_ displacements for protein trajectory *m*, $\Delta x_{m}={\left\{\Delta x_{m}\left(n\right)\right\}}_{n=1}^{N_{m}}$, $\Delta \hat{x}={\left\{\Delta x_{m}\right\}}_{m=1}^{M}$ is the set of *M* protein track displacements, $\hat{\pi }={\left\{\pi_{k}\right\}}_{k=1}^{K}$ is the set of variables which represent the fraction of the population of trajectories that realize diffusive state *k*, which is bounded and normalized: 0 ≤ *π*_*k*_ ≤ 1 and $\sum_{k=1}^{K}\pi_{k}=1$, $\hat{\Sigma }={\left\{\Sigma_{k}\right\}}_{k=1}^{K}$ is the set of covariance matrices which defines each diffusive state, and *P*(Δ**x**_*m*_∣**Σ**_*k*_) is the likelihood of protein trajectory *m* given by [34]:

$$P(\Delta x_{m}|\Sigma_{k})=\frac{1}{{\left(2\pi \right)}^{N_{m}/2}|\Sigma_{k}{|}^{1/2}}\text{exp}\;\left[-\frac{1}{2}\Delta x_{m}^{T}\Sigma_{k}^{-1}\Delta x_{m}\right],$$ (2)

where $\Delta x_{m}^{T}$ is the transpose, **Σ**_*k*_ is the covariance matrix for diffusive state *k*, ∣**Σ**_*k*_∣ is its determinant, and $\Sigma_{k}^{-1}$ is its inverse. Explicitly, the covariance matrix for a vector of displacements separated by Δ*t* undergoing normal diffusion is given by [34]:

$$\Sigma_{k}(i,j)=\{\begin{array}{ll} 2D_{k}\Delta t+2\sigma_{k}^{2}-4RD_{k}\Delta t & \;i=j\\ -\sigma_{k}^{2}+2RD_{k}\Delta t & \;i=j\pm 1\\ 0 & \;\text{otherwise} \end{array}$$ (3)

where *i* and *j* correspond to the row and column indices of the covariance matrix, respectively, *D*_*k*_ is the diffusion coefficient for diffusive state *k*, *σ*_*k*_ is the static localization noise for diffusive state *k*, and *R* is the motion blur coefficient [19, 34], which depends on the shutter state during the camera integration time. For a protein trajectory undergoing normal diffusion, $R\approx \frac{1}{6}\frac{\Delta t_{E}}{\Delta t}$, where Δ*t*_*E*_ is the exposure time [35]. For a shutter that is open throughout Δ*t*, as we assume in this paper, $R=\frac{1}{6}$.

Our goal is to determine the values of ${\left\{D_{k},\sigma_{k},\pi_{k}\right\}}_{k=1}^{K}$ that maximize Eq 1. Fortunately, GMMs are efficiently maximized with the expectation-maximization (EM) algorithm [36, 37]. In the expectation step, the posterior probability, *γ*_*mk*_, that protein trajectory *m* realizes diffusive state *k* given the covariance matrices of each diffusive state, **Σ**_*k*_, is calculated according to:

$$\gamma_{mk}=\frac{\pi_{k}P(\Delta x_{m}|\Sigma_{k})}{\sum {}_{j=1}^{K}\pi_{j}P(\Delta x_{m}|\Sigma_{j})}.$$ (4)

In the maximization step, the posterior probability is used to update the parameter estimates by first maximizing Eq 1 with respect to **Σ**_*k*_ and then solving for *D*_*k*_ based on the systems-level covariance-based estimator (CVE) which leads to the expression (see S2 Text for derivation):

$$D_{k}=\frac{1}{2\Delta tM_{k}}\sum_{m=1}^{M}\gamma_{mk}\left(\langle \Delta x_{m}{\left(n\right)}^{2}\rangle +2\langle \Delta x_{m}(n)\Delta x_{m}(n+1)\rangle \right)$$ (5)

where

$$\langle \Delta x_{m}{\left(n\right)}^{2}\rangle =\frac{1}{N_{m}}\sum_{n=1}^{N_{m}}\Delta x_{m}{\left(n\right)}^{2}$$

is the mean square displacement of the *N*_*m*_ displacements of trajectory *m*,

$$\langle \Delta x_{m}(n)\Delta x_{m}(n+1)\rangle =\frac{1}{N_{m}-1}\sum_{n=1}^{N_{m}-1}\Delta x_{m}(n)\Delta x_{m}(n+1)$$

is the mean correlation between nearest-neighbor displacements of trajectory *m* and

$$M_{k}=\sum_{m=1}^{M}\gamma_{mk}.$$

Similarly, the covariance-based maximization expression for $\sigma_{k}^{2}$ is given by:

$$\sigma_{k}^{2}=\frac{1}{2M_{k}}\sum_{m=1}^{M}\gamma_{mk}\langle \Delta x_{m}{\left(n\right)}^{2}\rangle -D_{k}\Delta t(1-2R).$$ (6)

We also maximize Eq 1 with respect to the population fraction of each diffusive state, *π*_*k*_, with the result that

$$\pi_{k}=\frac{M_{k}}{M}.$$ (7)

Because the posterior probabilities, *γ*_*mk*_, depend on *D*_*k*_, *σ*_*k*_, and *π*_*k*_, Eqs 5, 6 and 7 constitute coupled, non-linear equations for *D*_*k*_, *σ*_*k*_, and *π*_*k*_, respectively. The EM algorithm solves these equations iteratively [37]. In brief, the current estimates for the parameters, *D*_*k*_, *σ*_*k*_, and *π*_*k*_, are used to generate the analytical covariance matrix (Eq 3), which is used in the expectation step to evaluate the posterior probabilities (Eq 4). Then, the maximization step uses the posterior probabilities to re-estimate *D*_*k*_ (Eq 5), *σ*_*k*_ (Eq 6), and *π*_*k*_ (Eq 7). This procedure is iterated, until the change in the log-likelihood becomes smaller than a set threshold [37].

The extension to higher dimensions can be carried out facilely by treating each dimension separately. In this paper, we assume that protein trajectories undergo isotropic diffusion. Hence, we calculate the expectation step by averaging the posterior probability over each dimension using the same parameter estimates. For the maximization step, the maximized parameter estimates are calculated separately for each dimension and then averaged. At each step in the iteration procedure, the complete log-likelihood is calculated by summing the log-likelihood from each dimension.

### Perturbation expectation-maximization

Although the EM algorithm guarantees convergence to a maximum [37], convergence to the global maximum is not guaranteed. It turns out that global convergence is highly dependent on the initial parameter values for *D*_*k*_, *σ*_*k*_, and *π*_*k*_[38], and although the initialization sensitivity may be remedied by re-employing EM with a different set of initial parameters, this approach is very computationally expensive as it requires a large number of reinitialization trials to sufficiently explore parameter space. By contrast, we elect to employ a novel variant of the EM, that we term perturbation Expectation-Maximization (pEM), to directly handle EM convergence issues (S1 Data).

Initially, our pEM procedure employs the EM algorithm on the original set of protein trajectories with random initial parameter values. The mechanism by which pEM escapes from a local maximum is by reemploying the EM on a perturbed likelihood surface, which is generated with a Monte Carlo bootstrap set of the original protein trajectories [39]. Specifically, in each perturbation trial, the original protein trajectories are randomly sampled to create a new data set, with the same total number of trajectories as the original data set; however, some protein trajectories may be counted more than once, while other protein trajectories are excluded altogether. Consequently, each perturbation slightly alters the likelihood surface with the aim that a local maximum may no longer be a maximum in the perturbed likelihood surface. Since the perturbed likelihood surface may shift the location of the global maximum likelihood, we then verify whether a higher likelihood has truly been found by calculating the likelihood using the pEM-converged parameters with the original dataset. If the pEM-converged parameters indeed yield a higher likelihood, then the EM parameters are updated by reemploying the EM algorithm initialized with the pEM parameter estimates on the original dataset. Otherwise, the pEM estimates remain unchanged and we continue to perturb the likelihood surface with different Monte Carlo bootstrap datasets in search of an escape route. This process is repeated until a predetermined number of perturbations have been executed and yield no advance. The verification step guarantees only upwards movements along the unperturbed likelihood surface are permitted. When the global maximum is reached, each pEM trial terminates quickly as a higher likelihood cannot be found.

To generate each set of random initial values, *K* random numbers between 0 and 1 are drawn from a uniform distribution. The initial population fractions, ${\left\{\pi_{k}^{0}\right\}}_{k=1}^{K}$, are given by normalizing these random numbers so that the sum is equal to 1. The initial diffusivity values are set using the initial randomly-chosen population fractions and the empirical cumulative diffusivity distribution function. By dividing the cumulative distribution function into K regions proportional to the initial population fractions, the initial value of the diffusivity of diffusive state k ($D_{k}^{0}$) is then picked as the diffusivity corresponding to the midpoint of region k of the cumulative probability distribution, namely to $\sum_{j=1}^{k-1}\pi_{j}^{0}+\frac{\pi_{k}^{0}}{2}$. The initial static localization noise values are set to the mean CVE static localization noise estimates for each diffusive state. Thus, we achieve an initialization that serves as a non-parametric method to randomly sample from the observed distribution of diffusion coefficients.

Since the number of diffusive states is not known *a priori*, we repeat the pEM procedure for different numbers of diffusive states, finding the maximum likelihood in each case. However, the likelihood increases monotonically with increasing number of free parameters [36]. In other words, the maximum likelihood does not penalize for model complexity, which may lead to over-fitting. To maintain model parsimony, we employ the Bayesian Information Criterion (BIC) to penalize for the inclusion of additional diffusive states according to [36, 39]:

BIC=lnℒ−KlnM

where L is the maximum likelihood value, *K* is the assumed number diffusive states, and *M* is the number of protein trajectories. In this formulation, the model with the largest BIC score is selected.

### Generation of expression vectors

Generation of the pcDNA6/His-mEos2-Rho expression construct was achieved by classical molecular biology techniques. In brief, the coding sequence for mEos2 was extracted from a pSERTa-mEos2 vector (Addgene plasmid 20341) by digestion with BamHI and EcoRI (Promega, Madison, WI) restriction enzymes. The resulting coding DNA fragment was ligated into BamHI and EcoRI digested and alkaline phosphatase (Promega) treated pcDNA6/His-C (Life Technologies, Carlsbad, CA) vector. A c-terminal TGA stop codon carried in by the mEos2 coding sequence was mutated to GGA following a standard Stratagene quick change method. The Rho coding sequence was PCR amplified from a pTAG-RFP-RhoA expression vector (provided by Dr. William Cain) by PFU Ultra II DNA polymerase (Agilent Technologies, Santa Clara, CA) and the following phosphorylated primers: 5’-CCGACCATCCTCCAAAATC-3’ and 5’-GGATCCCTCCAGCAAGGT-3’ (Integrated DNA Technologies, Coralville, IA). The resulting PCR Rho fragment was blunt end ligated into EcoRV digested and alkaline phosphatase treated pcDNA6/his-mEos2 plasmid DNA. The resulting pcDNA6/His-mEos2-Rho vector was confirmed by Sanger sequencing.

### Cell culture and transfection

MCF10A cells were obtained from ATCC (Manassas, VI) and maintained in Dulbecco’s Modification of Eagle’s Medium/Ham’s F-12 50/50 medium (Cellgro, Manassas, VI) containing 5% fetal bovine serum, 50 *μ*g/mL Bovine Pituitary Extract (Sigma Aldrich), 0.5 *μ*g/mL hydrocortisone (Sigma Aldrich), 20 ng/mL human Epidermal Growth Factor (Sigma Aldrich), 10 *μ*g/mL insulin (Sigma Aldrich), 100 ng/mL cholera toxin (Sigma Aldrich) and 1% penicillin/streptomycin (Cellgro). 24hrs prior to transfection cells were plated at a confluence of 80% in 8-well nunc chambers (Lab-Tek). Transfections were carried out using X-tremeGENE HP transfection reagent (Roche) following manufacture’s recommended protocol and a transfection ratio of 4:1 (reagent:DNA). Cells were used for imaging 24 hours post transfection.

### Imaging setup

A custom built total internal reflection fluorescence (TIRF) system is assembled upon a Zeiss Axio Observer A1 body. Sample position is controlled by Physik Instrumente stage controller (C867). The system has 4 diode lasers used for fluorescence excitation: 50 mW 404 nm (Coherent Cube), 30 mW 488 nm (Coherent Sapphire), 50 mW 561 nm (Coherent Sapphire), and a 50 mW 642 nm (Coherent Cube). All four laser intensities are modulated by an acousto-optic tunable filter (AA Optical Electronic). The laser lines are expanded by a 5X beam expander and diverted to the back objective aperture with a quad dichroic filter (Semrock, Di01-R405/488/561/635). A movable achromatic doublet lens is used to focus the expanded beam onto the back focal plane of a 100X NA 1.46 Zeiss Plan Apo objective. TIRF is accomplished by translating the lens to the peripheral of the objective back aperture such that the emerging beam exceeds the total internal reflection critical angle. Excitation light is removed by a quad notch filter (Semrock NF01-405/488/557/640). The field of view is magnified 2X after the tube lens and the fluorescence image is projected on an Andor iXon DU897 EMCCD camera. Custom software was written in LabView (National Instruments) to control the camera, laser intensity, laser duration and image acquisition. Cells are maintained inside a stage incubator at 37C (Zeiss, TempControl 37–2) and 5% CO2 perfusion (Zeiss, CTI-Controller 3700) during imaging. The each cell was prebleached with 561 nm laser to remove background. Then, the samples were exposed to the 405 nm laser for 500 ms. Movies were taken subsequently at a frame rate of 31 Hz with a TIRF illumination created by the 561 nm laser.

### Single molecule pull down assay

Nunc Lab-Tek II chamber slides were cleaned with 1M NaOH. Mouse anti-human RhoA was incubated with glass (1 *μ*g/mL) for 1 hour. Unbound glass was blocked with 5% BSA for 1hr. HEK293T cell lysates containing mEos2-RhoA where incubate with antibody coated glass for 30 min and then washed with 1xPBS three times. Imaging was in PBS, areas were prebleached with 561 nm laser to remove background, then samples were photoconverted to reveal single molecule mEos2.

### Live cell protein tracking

Localization of individual Rho proteins was accomplished by placing each raw image through a spatial bandpass filter. The location of the peaks above a given threshold value were found and coarsely localized with a centroid moment calculation. Accurate localization estimates were then found by fitting each centroid to a radially symmetric Gaussian function [21]. The localized positions of the molecules in each frame are linked with a commonly used tracking algorithm for single protein tracking [17, 40]. The two user inputs the algorithm requires in order to link the protein positions are: the minimum number of frames, which constitute a trajectory, and the maximum distance a protein can travel from one frame to the next, which were set to 15 steps and 5 pixels (860 nm), respectively. These are typical values for sptPALM [17].

### Protein trajectory simulation procedure

Synthetic protein trajectories undergoing isotropic Brownian motion were constructed with the recursion [31]: *x*_*i*+1_ = *x*_*i*_ + (2*D*Δ*t*)^1/2^ *W*_*i*_ with *x*_1_ = 0, where *D* is the diffusion coefficient, Δ*t* is the time interval between positions, and *W*_*i*_ is a normally distributed random number with zero mean and unit variance. This process is carried out separately for two spatial dimensions and then combined to form the true two-dimensional (2D) synthetic protein trajectory. Motion blur is incorporated by simulating “micro-step” displacements for 1 ms time steps and averaging 32 successive positions. The net effect assumes that the exposure time is equal to the frame duration. Static localization noise is included by adding a normally distributed random number with zero mean and variance, $\sigma_{sim}^{2}$, to each motion-blurred position.

To recapitulate the track-length variability found in our experimental data, the length of each simulated trajectory is a random variable, *N*, given by *N* = (*N*_*max*_−*N*_*min*_)exp[−*rZ*] + *N*_*min*_, where *r* = (*N*_*max*_−*N*_*min*_)/(⟨*N*⟩−*N*_*min*_); *N*_*max*_ is the maximum length that a protein trajectory can realize and is set to 60; *N*_*min*_ is the minimum length and is set to 15; ⟨*N*⟩ is the average protein track length and is set to 25; Z is a uniform random number between 0 and 1. To create a collection of *M* heterogeneous protein trajectories, for each value of *k*, $\pi_{k}^{sim}M$ random-length tracks were simulated with a diffusion constant $D_{k}^{sim}$ and static localization noise $\sigma_{k}^{sim}$.

## Results

### pEM performance *in silico*

**Fig 2:**
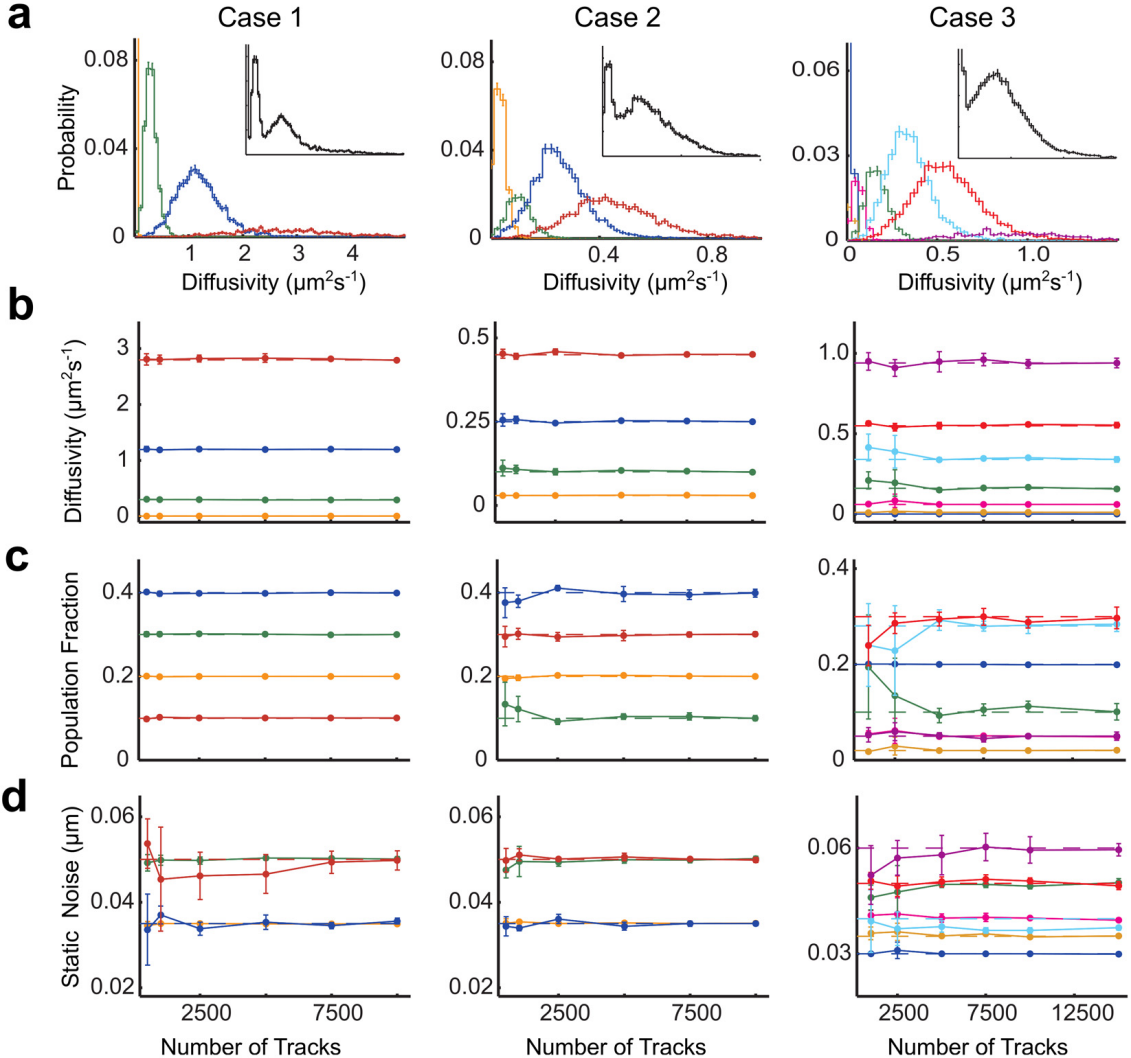
pEM performance on synthetic protein trajectories with variable localization noise. (a) The probability distributions of each diffusive state (shown in a different color) generated by the covariance-based estimator analysis on 10,000 protein trajectories for case 1 (left), case 2 (middle), and case 3 (right). The inset of each figure shows the total diffusivity distribution using the same scale as the main figure. (b-d) The average pEM estimates for each diffusive state, (b) diffusivity, (c) population fraction, and (d) static localization noise, determined by analyzing data sets with various number of protein trajectories given according to case 1 (left), case 2 (middle), and case 3 (right), assuming the correct model size for each case. Each color corresponds to a different diffusive state (a-d). Each data point represents the average estimate from five sets of protein trajectories and the error bars represent the observed standard deviation. The horizontal dashes represent the ground truth, *i.e*. the values input to the simulation, with the color corresponding to the diffusive state in question. In some cases, the horizontal dashes cannot be seen when pEM yields estimates near the simulated values.

To understand the strengths and limitations of pEM, it is necessary to appreciate the factors that contribute to the complexity of the system under study, which include: a large number of diffusive states; diffusive states with similar diffusivities, leading to overlapping component distributions; component distributions with small population fractions, leading to under-representation; and a restricted number of protein trajectories, leading to poorly defined component distributions. To elucidate the roles of these factors, we have generated three cases of synthetic protein trajectories, whose parameters are given in S1 Table: case 1 represents a relatively simple dataset, comprising four more-or-less non-overlapping and well-represented diffusive states (Fig 2a, left); case 2 represents a somewhat more complex dataset, comprising four overlapping but well-represented states (Fig 2a, middle); case 3 represents a very complex dataset, comprising protein trajectories containing 7 underlying diffusive states which include a number of overlapping and/or under-represented components (Fig 2a, right). To further test the capabilities of pEM, we apply different levels of static localization noise for each diffusive state, which may be realized experimentally as a consequence of dimerization or partial-quenching arising from either binding interactions or conformational changes. In S3 Text, we provide a detailed discussion of pEM’s improved estimation and efficiency over the alternative approach where multiple runs of the EM are performed with different initial parameters.

We explore how pEM performance depends on the number of protein trajectories, by varying the number of trajectories from a minimum of 500 to a maximum of 10,000 trajectories for case 1 and case 2, and from a minimum of 1,000 to a maximum of 15,000 trajectories for case 3 (see S1 Fig. for example trajectories). Remarkably, pEM was able to recover the correct number of diffusive states and assign reasonably accurate parameter values for all of the data sets of case 1, regardless of the number of trajectories analyzed (Fig 2b–2d, left). Even more remarkably, pEM was able to uncover the correct number of diffusive states with accurate parameter values for case 2, even for as few as 500 trajectories (Fig 2b–2d, middle). Clearly, the BIC is capable of reliably determining the number of diffusive states with high fidelity in these cases (S2 Fig). However, when pEM is applied to the data sets of case 3, the effect of analyzing a small number of protein trajectories results in unreliable parameter estimates (Fig 2b–2d, right). Specifically, when 2,500 protein trajectories or less were analyzed, pEM failed to consistently uncover all of the diffusive states, ultimately favoring a 6-diffusive-state model with more-or-less erratic parameter values. The inclusion of a sufficiently large number of protein trajectories, however, improved the accuracy and precision of the pEM estimates: When 5,000 protein trajectories or more were analyzed, pEM consistently uncovered a 7-diffusive-state model with reliable parameter values. Evidently, if enough trajectories were available to analyze, pEM was consistently able to accurately characterize some diffusive states (namely 1, 2, 3, and 7), despite the fact that some of these were under-represented, exhibiting population fractions as low as 0.02. Not surprisingly, the greatest variability in the estimates came from the diffusive states (namely 4, 5, and 6), which showed the most significantly overlapping component distributions (green, cyan, and red curves in Fig 2a, right). States 1, 2, and 3, on the other hand, lie close together in absolute terms, however the small widths of their diffusivity distributions render them statistically distinguishable.

### Comparison to other analysis methods *in silico*

To carry out a comparison of pEM to CDF and vbSPT, at a minimum, the static localization noise must be known for each diffusive state in order to be able to correct for the biased diffusivity estimates that emerge from CDF and vbSPT. Accordingly, we chose to simulate protein trajectories without any interconversions exhibiting the same localization noise for all diffusive states. For our CDF and vbSPT analyses, we took the localization noise to be known. For the pEM analysis, however, we continued to employ our standard procedure, which determines the static localization noise from the analysis itself.

When CDF is applied to 10,000 synthetic protein trajectories with diffusive states given according to either case 1 or case 2, we found that CDF fails to uncover the proper number of diffusive states, even when the simulation parameters are used as the initial fitting values (S2 Table). Nevertheless, the cumulative square displacements appear to be well fit with CDF (S3 Fig). When CDF is applied to protein trajectories *without* localization noise, for which CDF models properly, the quality of the CDF estimates improves, albeit only for very large sample sizes (S2 Table). Thus, CDF estimates should be treated with skepticism when trying to resolve the diffusive states of experimental protein trajectories.

vbSPT, on the other hand, was occasionally able to uncover the correct numbers of diffusive states with reasonably accurate parameter estimates, albeit after bias correction (S4 Fig). For synthetic protein trajectories generated according to case 1, vbSPT uncovered the correct number of diffusive states only when less than 2,500 protein trajectories were analyzed (S5 Fig). Paradoxically, the inclusion of more trajectories introduced an additional, spurious diffusive state. Specifically, the lowest diffusive state split into two distinct states with diffusivity values that lie about the true value. For case 2, vbSPT uncovered three diffusive states only, when less than 2,500 protein trajectories were analyzed (S6 Fig). With the inclusion of more protein trajectories, however, vbSPT was able to uncover the proper number of diffusive states with accurate estimates for the diffusivities. Even so, the population fraction estimates for the two slowest diffusive states became progressively worse with the addition of more protein trajectories. For case 3, vbSPT was only able to uncover 6 diffusive states even when analyzing 15,000 proteins trajectories (S7 Fig).

In general, we found vbSPT captured the more mobile diffusive states accurately, but was unsuccessful when applied to states with small diffusivities. Interestingly, among the states with small diffusivities (*D*_*k*_ ≤ 0.1 *μ*m^2^s^−1^), vbSPT nevertheless found significant transition probabilities (≥ 0.1 per step) between diffusive states, despite the fact that each simulated protein trajectory corresponds to the same diffusive state throughout. When vbSPT was applied to the same synthetic protein trajectories, but *without* localization noise, all diffusive states were accurately recovered, including negligibly small transition probabilities (S8 Fig). In the absence of localization noise, protein track displacements follow a Markov process. Thus, vbSPT, and more generally hidden Markov models [41, 42], describe the underlying diffusion process and kinetics accurately. However, experimental protein trajectories inevitably contain localization noise which introduces correlations between nearest-neighbor displacements. Thus, in general, analyses using standard hidden Markov models are only appropriate when the localization noise sources are negligible.

On the surface, the problem of uncovering the numbers of diffusive states and determining the properties of each diffusive state appears to be a cluster analysis problem [36]. Thus, the most direct approach would be to calculate the CVE estimates for each individual protein trajectory and apply a clustering algorithm to determine the underlying diffusive state properties for each cluster. However, the distribution of diffusion coefficients is not Gaussian, especially when the protein trajectories are short (S1 Text). Thus, applying a GMM approach on the empirical diffusion coefficients and static localization noises is improper. Moreover, non-parametric approaches, such as k-means clustering [36], also result in poor characterization of the underlying diffusive state properties (S9 Fig).

### Classification of protein trajectories to respective diffusive states

Beyond the identification of the diffusive states, that are present in the population of trajectories as a whole, it is also very interesting to consider the possibility of classifying individual protein trajectories into a particular diffusive state. In living cells, such a classification proffers the ability to explore the spatiotemporal dynamics of each diffusive state separately, and to determine additional properties of each diffusive state, such as average duration, directionality, *etc*. Fortunately, the posterior probability (Eq 4) gives the probability that a protein trajectory is generated from a given diffusive state. Although it would be possible for classification to be based on which state realizes the maximum posterior, information about the confidence of each diffusive state is lost upon definitive classification, which in turn, may lead to artifacts. Alternatively, the spatial distribution of the protein trajectories for each diffusive state may be better represented by retaining all of the information by rendering each protein trajectory with a color corresponding to the magnitude of the posterior probability as a heat map. The details of such a representation are described in S4 Text.

### Limitations of pEM

pEM makes two broad assumptions: (1) the underlying diffusive states are normal, and (2) there are no transitions between diffusive states. When these assumptions hold, we have shown that pEM is able to uncover the proper number of diffusive states and yield an accurate characterization of each diffusive state, that is, for protein trajectories with variable lengths similar to our experimental data. In S5 Text, we explore pEM’s performance dependence on the protein trajectories length. Since the width of the distribution of each underlying diffusive state is directly related to the length of the protein trajectory (S1 Text), pEM performs better with longer protein trajectories. As the protein trajectories become shorter, the diffusivity distribution of each diffusive state broadens leading to more overlap between diffusive states, thereby increasing the complexity.

Longer protein trajectories, however, provide more opportunities for transitions between diffusive states to occur. In S6 Text, we test pEM’s performance on synthetic protein trajectories which can transition between different diffusive states and for protein trajectories which contain a non-normal diffusive state. We find, in general, that even when transitions are present, pEM still yields reasonable estimates for the underlying diffusivity and static localization noise for each diffusive state. However, the population fractions become increasingly unreliable when more transitions are present. In comparison, even though vbSPT incorporates the ability to transition between diffusive states in principle, even in this case, we found that vbSPT still underperforms pEM, which we attribute to its neglect of nearest-neighbor correlations.

Since transition rates and the presence of non-normal modes of diffusion are not known *a priori* for experimental protein trajectories, we demonstrate how to test for transitions and for the presence of non-normal modes of diffusion in S6 Text.

### Application of pEM to Rho GTPase protein trajectories in live cells

**Fig 3:**
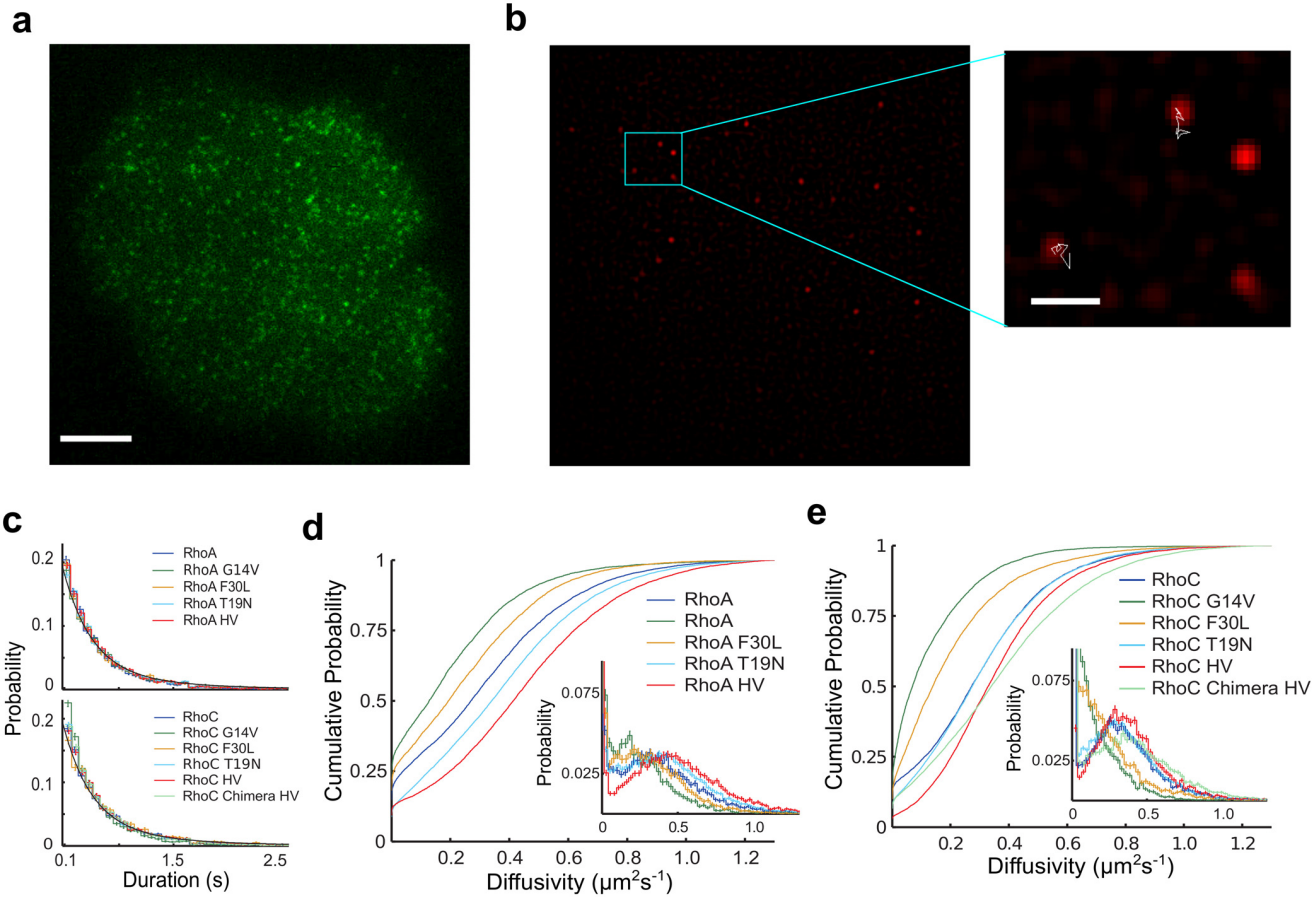
Rho GTPase ensemble results. (a) Representative cell expressing mEos2-RhoA prior to photo conversion visualized under 488 nm TIRF illumination. Scale bar is 10 *μ*m. (b) The same cell after photoconversion now visualized under 561 nm TIRF illumination. The magnified selection depicts two sufficiently long-lived fluorescent spots with their spatial trajectories shown in white. The fluorescent spots without a trajectory did not meet our criterion of a minimum track length of 15 steps. Scale bar in the magnified image is 2 *μ*m. (c) Empirical probability distribution of the protein trajectory durations of the RhoA wild type+mutants (top) and RhoC wild type+mutants (bottom). (d-e) Cumulative probabilities of the diffusivity for (d) RhoA wild type+mutants and (e) RhoC wild type+mutants from covariance-based estimator analysis on individual protein trajectories. The inset displays the corresponding binned probability distributions. The error bars in the binned distributions represent the standard error of the mean (c-e). For each construct, we extracted protein trajectories across several cells. The total numbers of trajectories analyzed for each construct is given in S3 Table.

As a first application of pEM to a biological system, we genetically tagged RhoA and RhoC to the C-terminal of the photo-convertible mEos2 fluorophore and employed sptPALM to examine their single molecule membrane associated dynamics in live MCF10A human epithelial cells, corresponding to a non-tumorigenic, spontaneously immortalized breast epithelial cell line [43], resting on a glass surface. Fig 3a shows an image of a representative cell expressing mEos2-RhoA observed in green light. The stochastic process of photoconversion alters the fluorescence properties of a small portion of the population, such that under red light, individual Rho proteins can be reliably monitored without making any modifications to Rho’s endogenous concentration levels (Fig 3b).

Membrane-associated Rho GTPases undergo numerous protein-protein interactions leading to variability in diffusive behaviors, which we envision to fall into three broad categories: (1) *active* or *inactive* unbound Rho undergoing free diffusion along the membrane, (2) *active* or *inactive* Rho bound to a single regulator/effector, undergoing reduced diffusion, and (3) *active* Rho associated with large immobile macromolecular complexes. To explore the breadth of Rho interactions, we investigated the diffusive behaviors of various point mutated functional mutants each tagged with mEos2 fluorophore. For brevity, we will refer to these chimeras only by their Rho designation henceforth. Specifically, the G14V point mutant represents a constitutively *active* mutant incapable of GTP hydrolysis [44, 45], which we expect to be predominately associated with macromolecular signaling complexes. The F30L mutant displays increased GDP-to-GTP exchange rate, while maintaining normal GTP hydrolytic activity [46], leading to spontaneous activation and effectively removing prolonged *inactive* states. The T19N mutant is a dominant negative mutant with reduced binding affinity for GTP [45] and RhoGDI [47]. This mutation prevents activation and leads to strong binding to RhoGEFs [48]. To determine what role the hypervariable (HV) domain plays in Rho diffusive dynamics, we generated truncation mutants, which contain only the last seventeen C-terminal amino acids of RhoA or RhoC fused to mEos2. Lastly, to extend our investigation into the role of the HV domain, we generated a chimeric mutant consisting for the N-terminal G-protein domain of RhoC fused to the HV domain of RhoA.

Unfortunately, the photoinstability of mEos2 prevented us from quantifying Rho’s residence time on the membrane. Specifically, we characterized Rho’s photostability using a pull down-assay, immobilizing mEos2-RhoA on a glass surface, monitoring the fluorescence intensity at each fluorescent spot over time, and employing a two-state hidden Markov Model to extract the flurophore on-times and off-times (S10 Fig). The cumulative distribution functions of the on- and off-times so-obtained were fit using a double exponential function (S10 Fig). For both the on- and off-time distributions, the characteristic time of the fast component is shorter than the 15 frame minimum cutoff, that we set for each protein trajectory. For the slowly-decaying component, the off-time distribution showed a best-fit characteristic time of 3.2 s (100 frames), while the on-time distribution showed a best-fit characteristic time of 0.8 s (25 frames). This value for the on-lifetime is very similar to the characteristic duration of our experimental protein trajectories in live cells, irrespective of mutation (Fig 3c), strongly suggesting that the observed duration of these live-cell trajectories corresponds to the on-time of the fluorophore, and therefore that the typical duration of Rho membrane association is significantly longer than 0.8 s (25 frames). Only a subset of the observed fluorescent spots, namely those that met our criterion for a minimum track length of 15 frames, were reconstructed into protein trajectories (Fig 3b).

To compare ensemble differences in the diffusivities between various mutants, we generated a cumulative distribution of diffusivities for each Rho variant by analyzing individual protein trajectories with the covariance-based estimator [49] (Fig 3d and 3e). These distributions illustrate significant differences in the ensemble diffusive behavior among mutants. For instance, the activating mutants, G14V and F30L, shift the population towards reduced diffusivities for both RhoA and RhoC. Conversely, the dominate negative inactive mutant T19N, shifts the population towards higher diffusivities for RhoA. Wild type RhoC, on the other hand, appears nearly identical to RhoC T19N, with a slight divergence occurring at slower diffusivities. The hypervariable truncation mutant, HV, for RhoA and RhoC, and RhoC Chimera HV, exhibit population shifts towards even faster diffusivities relative to wild type. Interestingly, the majority of the diffusivity distributions exhibit a bimodal shape (Fig 3d and 3e, inset). However, it would be naive to infer on this basis that Rho experiences two main biochemical interactions, because of the counterexample provided by the diffusivity distributions of the simulated protein trajectories for case 2 and case 3 (Fig 2a, inset), which also appear bimodel, but which in fact correspond to significantly more complex diffusive heterogeneity. Clearly, diffusivity distributions are unreliable for resolving the system of diffusive states with fidelity.

Instead, to uncover the diffusive states, exhibited by Rho, we applied pEM to the population of protein trajectories across multiple cells for each Rho variant (S3 Table).

To validate that the assumptions made by pEM hold for our experimental data, and also bolstering our analysis methodology and the diffusive states that result, we verified *a posteriori*, first, that the observed protein trajectories of Rho GTPase on average remain in the same state diffusive state throughout, and secondly, that the underlying diffusion modes all correspond to normal diffusion over the timescales relevant to our experiments (S7 Text). Example RhoA protein trajectories for each diffusive state is shown in S1 Fig.

**Fig 4:**
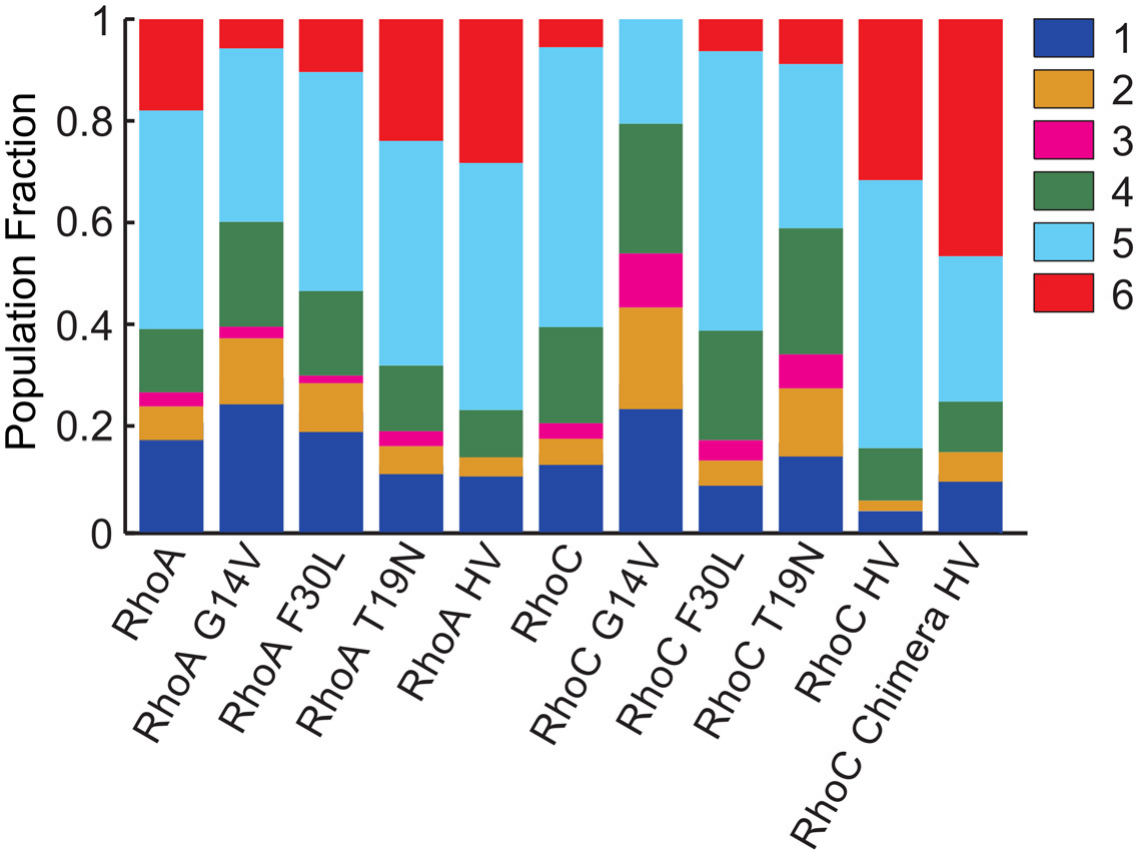
Diffusive state population fractions for Rho GTPase. Stacked bar graph of the population fractions for each Rho GTPase determined via our pEM approach. The color corresponds to a given diffusive state. The standard error for each diffusive state population fractions is < 0.02.

As shown in Table 1, in most cases, pEM uncovered six diffusive states ranging in diffusivity from about 0.0007 *μ*m^2^s^−1^ to about 0.7 *μ*m^2^s^−1^. Strikingly, the diffusivity and localization noise for each state are similar across all five mutants of RhoA and all six mutants of RhoC, strongly suggesting that the same interactions are manifest within all of these variants. By contrast, the population fractions of the different diffusive states vary significantly among the different variants (Fig 4). In general, Rho proteins reside predominantly in fast diffusing states (states 4–6) across all variants, with the exception of RhoC G14V. Nevertheless, the activating mutants, G14V and F30L, exhibit a decreased population of states 4–6 and a corresponding increase in the populations of states 1 and 2, compared to the dominant negative mutant T19N, and the HV truncation mutant, as we expected. These observations suggest that with activation, fewer protein trajectories populate the faster diffusive states, implying that they are more likely to be incorporated into binding interactions with large macromolecular complexes, resulting in a reduced diffusivity and therefore a higher population of states 1 and 2. Interestingly, the variability of the population fractions between states 1 and 2 across the RhoA mutants was much less pronounced, in comparison to the RhoC mutants. Moreover, wild type RhoA yields population fractions intermediate between those of constitutively active, RhoA G14V mutant and those of the dominant negative, RhoA T19N mutant. On the other hand, the population fractions for wild type RhoC are more closely aligned with the dominant negative RhoC T19N.

**Table 1:** Diffusive states of Rho GTPases acquired via pEM. The diffusivities (*μ*m^2^s^−1^), static localization noises (*μ*m), and population fractions for RhoA, RhoC, and various functional mutants are presented with a subscript corresponding to a respective diffusive state. Error bars are not presented for the reasons stated in S3 Text.

	RhoA	RhoA G14V	RhoA F30L	RhoA T19N	RhoA HV	RhoC	RhoC G14V	RhoC F30L	RhoC T19N	RhoC HV	RhoC Chimera HV
*D*_1_	0.0002	0.0001	0.0001	0.0005	0.0002	0.0004	0.0020	0.0011	0.0011	0.0003	0.0012
*D*_2_	0.0194	0.0187	0.0148	0.0308	0.0189	0.0227	0.0335	0.0237	0.0300	0.0231	0.0357
*D*_3_	0.0779	0.0761	0.0704	0.0848		0.0836	0.0855	0.0899	0.0790		
*D*_4_	0.1729	0.1538	0.1564	0.1726	0.1755	0.1856	0.1521	0.1503	0.1893	0.1533	0.1366
*D*_5_	0.3858	0.3468	0.3682	0.4340	0.4706	0.4173	0.3265	0.3122	0.4106	0.3359	0.3203
*D*_6_	0.6747	0.7259	0.6575	0.6749	0.7368	0.6331		0.5751	0.6112	0.5547	0.5576
*σ*_1_	0.0394	0.0398	0.0399	0.0397	0.0405	0.0384	0.0394	0.0409	0.0410	0.0403	0.0424
*σ*_2_	0.0572	0.0548	0.0577	0.0519	0.0543	0.0541	0.0455	0.0508	0.0534	0.0467	0.0505
*σ*_3_	0.0349	0.0338	0.0339	0.0355		0.0421	0.0367	0.0368	0.0397		
*σ*_4_	0.0499	0.0503	0.0523	0.0472	0.0468	0.0508	0.0472	0.0500	0.0485	0.0434	0.0466
*σ*_5_	0.0520	0.0544	0.0579	0.0503	0.0505	0.0525	0.0486	0.0527	0.0538	0.0484	0.0521
*σ*_6_	0.0500	0.0532	0.0640	0.0535	0.0601	0.0710		0.0638	0.0794	0.0557	0.0554
*π*_1_	0.1727	0.2428	0.1884	0.1066	0.1020	0.1247	0.2336	0.1410	0.0842	0.0340	0.0914
*π*_2_	0.0661	0.1296	0.0963	0.0552	0.0377	0.0504	0.1992	0.1333	0.0492	0.0205	0.0583
*π*_3_	0.0279	0.0228	0.0145	0.0279		0.0304	0.1068	0.0668	0.0390		
*π*_4_	0.1237	0.2065	0.1658	0.1293	0.0923	0.1894	0.2544	0.2476	0.2153	0.1029	0.0987
*π*_5_	0.4301	0.3403	0.4307	0.4414	0.4849	0.5498	0.2061	0.3227	0.5493	0.5259	0.2857
*π*_6_	0.1795	0.0580	0.1043	0.2395	0.2831	0.0553		0.0887	0.0630	0.3167	0.4659

To quantify the overall activation state of wild type cells, we carried out a least squares minimization of the difference between the cumulative diffusivity distribution of wild type Rho with the weighted sum of the cumulative diffusivity distributions of the activation extremes, namely G14V and T19N, where the weights are the free parameters (S8 Text). This procedure reveals that RhoA’s diffusive behavior was 32% similar to that of RhoA G14V and 68% similar to that of RhoA T19N; while, RhoC exhibited a stronger imbalance with its behavior 5% similar to that of RhoC G14V and 95% similar to that of RhoC T19N. Evidently, wild type RhoA exhibits a significant population of both active and inactive states, albeit with a slight imbalance towards inactive, in good agreement with our conceptual model (Fig 1). Wild type RhoC, on the other hand, exhibits diffusive behaviors more closely aligned with global inactivation.

The spatial distribution of Rho proteins for each diffusive state, provided by the pEM’s posterior-weighted classification (S4 Text), reveals that Rho proteins are ubiquitous across the plasma membrane, irrespective of their activation state (S11 and S12 Figs). Moreover, we do not observe any significant difference between the expression levels of RhoA and RhoC, nor among the various mutants.

## Discussion

Contrary to our expectations that HV mutants would undergo few protein-protein binding interactions, and would therefore exhibit a limited number of diffusive states, Table 1 reveals that HV mutants yielded nearly the same number of diffusive states as the full length Rho mutants. This observation suggests that the majority of the diffusive states may be related to interactions involving the HV domain and the heterogeneous environment of the plasma membrane inside live cells [50, 51]. In particular, RhoA HV shows a significant population fraction of very slow diffusive states (states 1 and 2), indicating its incorporation into large macromolecular complexes. One possible explanation is the presence of an import phosphorylation site in RhoA’s HV (S188) domain that is not found in RhoC. Phosphorylation of this site in RhoA has been shown to act as an important negative regulatory signal, increasing RhoA’s affinity for RhoGDI, leading to cytosolic sequestration [52]. It is conceivable that RhoA HV truncation mutants can be recognized as substrate for kinases leading to binding and reduced diffusion. cAMP-dependent kinase A (PKA) has been shown to phosphorylate RhoA on serine 188 [53]. PKA-anchoring proteins have also been described to provide spatiotemporal specificity for PKA signaling by localizing PKA to specific cell organelles, including the plasma membrane [54]. Interestingly, RhoC HV, which does not have this phosphorylation site in its hypervariable domain, yields significantly lower populations of states 1 and 2. Strikingly, RhoC Chimera HV, which is constructed with the G-domain of RhoC and the hypervariable domain of RhoA, recovers increased population fractions of the very slow diffusive states to similar levels as RhoA HV.

Although mutants with the HV tail alone may realize nearly all of the diffusive states found in the intact Rho complex, the wide variability in the population fractions across different mutants suggests that the HV tail is not solely responsible for the interactions of each diffusive state. For instance, relative to their wild type counterparts, activating mutants, G14V and F30L, yield larger population fractions of the very slow diffusive states (states 1 and 2), and smaller population fractions of the fast diffusive states (states 4–6), while the reverse trend is observed for the dominant negative mutant, T19N. State 3, on the other hand, was absent from HV mutant populations suggesting that this state requires G-domain specific interactions.

Interestingly, state 1 and state 3, consistently yield a smaller static localization noise across the full length Rho mutants (Table 1). We hypothesize that this result is because state 1 and state 3 are prevalent on particular regions of the membrane, that are slightly closer to the microscope coverslip, where the intensity of the exponentially-decaying evanescent TIRF excitation is higher. In principle, the cell membrane may be locally closer to the coverslip near focal adhesions, where Rho GTPases and integrins have been shown to play an interconnected role in focal adhesion assembly [45, 55, 56]. The diffusive behavior of integrins has been reported to undergo cycles of immobilization and free diffusion on focal adhesions with a reported diffusion coefficient that is similar to the diffusivity that we find for state 3 [25]. Since the HV mutants do not exhibit this population, we hypothesize that state 3 corresponds to the diffusion of a small complex, perhaps with integrins themselves or some protein mediator [56, 57]. For RhoA, a similar population fraction of state 3 is found across all variants, irrespective of activation state. RhoC, on the other hand, was more variable, yielding a significantly larger population of state 3 for the activating mutants, G14V and F30L. By contrast, wild type RhoC and the dominant negative RhoC (T19N) yielded lower population fractions of state 3, similar to RhoA. Since overexpression of RhoC has been shown to lead to an increase in actin stress fiber and focal adhesion contact formation [58], it is possible that RhoC has stronger interactions with focal adhesions when activated. Although the T19N mutants are expected to be unable to bind GTP or downstream effectors, the existence of these states may be the result of association with membrane localized RhoGEF complexes [59, 60].

Since constitutively active RhoA does not drive large scale population shifts to the slow diffusive states (states 1 and 2) relative to wild type RhoA, we postulate that endogenous activation alone may not be a sufficient condition for efficient binding to downstream effectors. Rather, a higher level of coordination with other signaling proteins is more likely to be required to facilitate RhoA’s ability to bind to downstream effectors [61–63], and/or activate the downstream effectors themselves [64, 65]. The high expression levels and ubiquitous spatial distribution of RhoA throughout the cell may serve to enable a faster signaling response to exogenous signals by removing the requirement of being recruited from greater distances along the cell membrane. Although active RhoA does not efficiently bind to large macromolecular complexes in the absence of exogenous stimuli, RhoA still likely undergoes a constant cycling between active and inactive states as a regulatory mechanism to allow RhoA to be readily available throughout the cell, but also to mitigate overreaction to spurious exogenous stimuli.

On the other hand, constitutively active RhoC G14V can bind more efficiently to downstream effectors, even in a resting cell. Thus, RhoC may either have more available potential downstream binding partners readily available, and/or RhoC does not require the same extent of orchestration with other signaling proteins as RhoA. According to this point of view, because RhoC exhibits similar high expression levels as RhoA, and is also ubiquitously distributed throughout the cell, RhoC requires more stringent regulation to prevent activation.

An important caveat to the present study stems from not knowing how the underlying biological signaling pathways may be altered with global activation [66], limiting the depth of information that can be extracted. In addition, further controls, which perturb specific biochemical interactions with potential downstream effectors, are undoubtedly required to determine what interactions correspond to each diffusive state. Finally, this study does not address the role of RhoGDI, which enforces an additional level of regulation by shuttling Rho on and off of the membrane, and possibly between different membrane domains [52].

Nevertheless, our study clearly demonstrates the power and potential of pEM by (1) uncovering the number of diffusive states, (2) determining the properties of each such diffusive state, and (3) classifying statistically each protein’s trajectory to a respective diffusive state. In the future, it will be interesting to see how the propensities for each diffusive state change spatially and temporally under various kinds of exogenous stimulation in both healthy and cancerous cells. More generally, we envision that pEM will have a broad impact on studies of a wide range of biological systems where different biochemical interactions result in distinct diffusive behaviors. The importance of uncovering the diffusive states of a protein, and eventually characterizing the transitions between different diffusive states within a protein’s trajectory is to permit the endogenous *in vivo* biochemistry of the diffusing protein to be probed and monitored within a live cell with single molecule resolution. Although pEM, in its current formulation, does not yet include transitions between different diffusion states, the determination of the number of diffusive states and their corresponding properties certainly brings us closer to realizing this transformational goal. Beyond single particle tracking data, pEM may help address the general challenge of global maximization via EM.

## Supporting Information

S1 Data Matlab-based graphical user interface for pEM with working examples. (ZIP)

S1 Table Simulation parameters for synthetic particle trajectories. Diffusivities, static localization noises, and population fractions used to generate synthetic protein trajectories are presented for case 1, case 2, and case 3. The columns represent the set of parameter values for each diffusive state.
(PDF)

S2 TableCDF parameter estimates for synthetic particle trajectories.
*D* ^*sim*^ and *π* ^*sim*^ represent the parameters used to simulate 10,000 synthetic protein trajectories with motion blur and a constant static localization noise, *σ* ^*sim*^ = 0.05 *μ*m, with diffusivities and population fractions given according to case 1 and case 2. The cumulative distribution function (CDF) parameter estimates are determined by applying a non-linear least squares fit to the cumulative square displacements, Δ*r* ^2^ = Δ*x* ^2^ + Δ*y* ^2^, across the population of synthetic protein trajectories according to [30]: $CDF\left(\Delta r^{2}\right)=1-(\sum_{k=1}^{K}\pi_{k}e^{-\Delta r^{2}/\left(2\rho_{k}\right)})$, where *ρ*_*k*_ = 2*D*_*k*_Δ*t* + 2*σ* ^2^ − 4*RD*_*k*_Δ*t*, {*D*_*k*_} are the diffusion coefficients, and {*π*_*k*_} are the population fractions which satisfy the normalization, $\sum_{k=1}^{K}\pi_{k}=1$. Here, *ρ*_*k*_ and *π*_*k*_ are the free parameters and the normalization constraint for *π*_*k*_ is enforced by replacing the population fraction of the *K*th diffusive state, *π*_*k*_, with $1-\sum_{i=1}^{K-1}\pi_{k}$. The fit is applied over the range 0 to ∞. *D*_*CDF*_ and *π*_*CDF*_ represent the CDF values from fitting the cumulative square displacements of the synthetic protein trajectories when using the simulated values as the initial guess and a known static localization noise (S3 Fig). *D*_*true*_ and *π*_*true*_ represent the CDF estimates when applied to the same protein trajectories, but without localization noise, and using the simulated values as the initial guess. Units of *D*, *μ*m^2^s^−1^.(PDF)

S3 Table Numbers of Rho protein trajectories used for pEM analysis. (PDF)

S1 Fig Example single protein trajectories. Example single protein trajectories for each diffusive state (shown in a different color) from synthetic protein trajectories given according to (a) case 1, (b) case 2, and (c) case 3. The underlying diffusivity of each diffusive state increases from left to right. (d) Shows example experimental RhoA trajectories based upon classification to each diffusive state. Protein trajectories, which had a posterior probability greater than 0.95 for the given diffusive state, were randomly selected for case 1 and case 2. Protein trajectories which have at least 40 steps and a posterior probability greater than 0.95 for the given diffusive state, were randomly selected for case 3 and for the experimental RhoA trajectories. The scale bar in each case represents 5 *μ*m.
(TIFF)

S2 Fig Bayesian information criterion scores for synthetic particle trajectories. BIC scores for various model sizes, acquired by applying pEM to (left) 500 synthetic protein trajectories corresponding to case 1 with a constant static localization noise, (middle) 1,000 synthetic protein trajectories corresponding to case 2 with a constant static localization noise, and (right) 5,000 synthetic protein trajectories corresponding to case 3 with variable static localization noises. Each plot shows the BIC score from 10 different initial parameter values (each shown in a different color). The maximum BIC score is consistently found for a four diffusive state model for cases 1 and 2, and seven diffusive state model for case 3. Evidently since the variability of the BIC score is smaller than its change for different number of states for all 3 cases, the BIC is a reliable method for model selection in this case.
(TIFF)

S3 Fig CDF of synthetic particle trajectories. The cumulative distribution of the square displacements, determined from 10,000 synthetic protein trajectories, corresponding to case 1 (left) and case 2 (right), is shown as the blue curve, while the best fit CDF is shown as the red curve. The fitted parameters, namely the diffusivity, *D*_*k*_, and the population fraction, *π*_*k*_, for each diffusive state, *k*, are given in S2 Table.
(TIFF)

S4 Fig vbSPT diffusivity estimates prior to bias correction for synthetic particle trajectories. Solid circles are the uncorrected diffusivity estimates, determined using vbSPT, for various numbers of synthetic protein trajectories, corresponding to case 1 (left), case 2 (middle), and case 3 (right). Different colors correspond to different diffusive states. Each data point represents the average of five sets of synthetic protein trajectories and the error bars represent the standard deviation. Where the error bars are not visible, they are smaller than the symbol size. The solid lines are a guide-to-the-eye. The horizontal dashed lines represent the ground truth, *i.e*. the values of the diffusion coefficients that are input to the simulation. The color of each dashed line indicates the corresponding underlying diffusive state. vbSPT was implemented with the open-source software described in Ref. [32]. Since the vbSPT framework does not directly incorporate localization noise, the diffusivity estimates generated by vbSPT are biased, except at particular values of *σ* ^2^ and *D*, at which the effects of static and dynamic localization noise on-average cancel, *i.e*. for 2*σ* ^2^ − 4*DR*Δ*t* ≈ 0. For diffusivities below this threshold value, the estimates exhibit a positive bias due to the dominant static localization noise. Above this threshold value, the estimates exhibit a negative bias due to the dominant dynamic localization noise.
(TIFF)

S5 FigComparison of pEM and vbSPT for synthetic particle trajectories given according to case 1.Diffusivity and population fraction estimates for various numbers of synthetic protein trajectories, corresponding to case 1, but with a constant static localization noise, *σ* = 0.05 *μ*m, for pEM (left) and vbSPT (right). The static localization noise is assumed to be known for vbSPT, but must be determined by pEM (bottom-left). Each color corresponds to a different diffusive state. Each data point represents the average of five sets of synthetic protein trajectories and the error bars represent the observed standard deviations. The solid lines are guides-to-the-eye. The horizontal dashed lines represent the ground truth, *i.e*. the values input to the simulations. The color of each dashed line indicates the corresponding diffusive state. Because the diffusivity estimates generated by vbSPT are biased, in order to present the analyis of protein trajectories with localization noise based on vbSPT, we propose a *post hoc* correction to the diffusivity, determined by vbSPT, namely $D_{k}^{vbSPT}$, to mitigate the bias, namely $D_{k}=\frac{D_{k}^{vbSPT}\Delta t-\sigma^{2}}{(1-2R)\Delta t}$. In this figure and in S6 and S7 Figs, the displayed results obtained using vbSPT have had this correction applied.(TIFF)

S6 Fig Comparison of pEM and vbSPT for synthetic particle trajectories given according to case 2. Diffusivity and population fraction estimates for various numbers of synthetic protein trajectories corresponding to case 2, but with a constant static localization noise, *σ* = 0.05 *μ*m, for pEM (left) and vbSPT (right). The static localization noise is assumed to be known for vbSPT but must be determined by pEM (bottom-left). Each color corresponds to a different diffusive state. Each data point represents the average of five sets of synthetic protein trajectories and the error bars represent the observed standard deviations. The solid lines are guides-to-the-eye. The horizontal dashed lines represent the ground truth, *i.e*. the values input to the simulation. The color of each dashed line indicates the corresponding diffusive state.
(TIFF)

S7 Fig Comparison of pEM and vbSPT for synthetic particle trajectories given according to case 3. Diffusivity and population fraction estimates for various numbers of synthetic protein trajectories, corresponding to case 3, but with a constant static localization noise, *σ* = 0.05 *μ*m, for pEM (left) and vbSPT (right). The static localization noise is assumed to be known for vbSPT but must be determined by pEM (bottom-left). Each color corresponds to a different diffusive state. Each data point represents the average of five sets of synthetic protein trajectories and the error bars represent the observed standard deviations. The solid lines are guides-to-the-eye. The horizontal dashed lines represent the ground truth, *i.e*. the values input to the simulation. The color of each dashed line indicates the corresponding diffusive state.
(TIFF)

S8 Fig vbSPT parameter estimates for particle trajectories without localization noise. Diffusivities and population fractions, given by vbSPT applied to various numbers of synthetic protein trajectories without localization noise, corresponding to case 1 (left), case 2 (middle), and case 3 (right). Each color corresponds to a different diffusive state. Each data point represents the average of five sets of synthetic protein trajectories and the error bars represent the observed standard deviation. The solid lines are guides-to-the-eye. The horizontal dashed lines represent the ground truth, *i.e*. the values input to the simulation. The color of each dashed line indicates the corresponding diffusive state.
(TIFF)

S9 Fig Performance of k-means clustering applied to synthetic particle trajectories. Diffusivity, static localization noise, and population fraction estimates for various numbers of synthetic protein trajectories, corresponding to case 1 (left) and case 2 (right) determined on the basis of k-means clustering applied to the diffusivity and static localization noise estimates for each protein trajectory [36]. Here, the covariance-based estimator was used to determine the diffusivity and static localization noise for each protein trajectory [49]. The k-means cluster for the correct model size was employed with a random initialization for each data set. Each data point represents the average of five sets of synthetic protein trajectories and the error bars represent the observed standard deviations. The solid lines are guides-to-the-eye. The horizontal dashed lines represent the ground truth, *i.e*. the values input to the simulation. The color of each dashed line indicates the simulated diffusive state. Each color of the k-means estimates corresponds to the closest diffusive state, except that one of the diffusive states found by k-means clustering is so far away from any actual diffusive state that it is given a different color (magenta). Because the performance of k-means clustering is so poor for case 1 and case 2, we did not test this method for case 3.
(TIFF)

S10 FigPhotostability of mEos2-RhoA.(a) Representative image from a pull-down assay of mEos2-RhoA. The inset shows the intensity profile of a single fluorophore within the red box. Scale bar is 10 *μ*m. (b) Representative intensity time series taken from each point-spread function (blue) and the states found using hidden Markov models (red). (c) The cumulative distribution of residence times in the on state (green) and the off state (blue). The fits represent a two component cumulative exponential fits given by: $y=1-w\;\text{exp}\;\left[-t/\tau_{1}\right]-(1-w)\;\text{exp}\;\left[-t/\tau_{2}\right],$ where *w* represent the fraction of component 1 decays, and *τ*_1_ and *τ*_2_ correspond to the characteristic residence times for component 1 and 2, respectively. The characteristic on-residence times were *τ*_1_ = 0.26 s and *τ*_2_ = 0.79 s with *w* = 0.6. The characteristic off-residence times were *τ*_1_ = 0.17 s and *τ*_2_ = 3.2 s with *w* = 0.33.(TIFF)

S11 Fig Spatial distribution of RhoA protein trajectories within a representative cell. Each column corresponds to a representative cell of RhoA, RhoA G14V, RhoA F30L, RhoA T19N, and RhoA HV. For each of these cells, the top row shows 2,000 protein trajectories, rendered using a false color scale, with the color corresponding to the diffusivity estimates found using the CVE. The images shown in rows 2 through 7 constitute a posterior-weighted localization map for each diffusive state in each cell. Specifically, the centroid of each protein trajectory is rendered as a blue square with a transparency equal to one-third of the posterior probability that the track in question realizes that row’s diffusive state. Thus, dark blue regions represent regions inside the cell where there is a high posterior probability of finding one or more protein trajectories corresponding to the diffusive state in question. Pale and white regions represent regions of low and zero probability, respectively, to find a protein trajectory corresponding to that diffusive state. The cell boundary is outlined in black in each panel. Scale bar is 10 *μ*m.
(TIFF)

S12 Fig Spatial distribution of RhoC protein trajectories within a representative cell. Each column corresponds to a representative cell of RhoC, RhoC G14V, RhoC F30L, RhoC T19N, RhoC HV, and RhoC Chimera HV. For each of these cells, the top row shows 2,000 protein trajectories, rendered using a false color scale, with the color corresponding to the diffusivity estimates found using the CVE. The images shown in rows 2 through 7 constitute a posterior-weighted localization map for each diffusive state in each cell. Specifically, the centroid of each protein trajectory is rendered as a blue square with a transparency equal to one-third of the posterior probability that the track in question realizes that row’s diffusive state. Thus, dark blue regions represent regions inside the cell where there is a high posterior probability of finding one or more protein trajectories corresponding to the diffusive state in question. Pale and white regions represent regions of low and zero probability, respectively, to find a protein trajectory corresponding to that diffusive state. The cell boundary is outlined in black in each panel. Scale bar is 10 *μ*m.
(TIFF)

S1 Text Distribution of diffusion coefficients. (PDF)

S2 Text Derivation of likelihood framework for diffusive states. (PDF)

S3 Text Performance comparison of pEM versus standard EM. (PDF)

S4 Text Classifying protein trajectories to respective diffusive states. (PDF)

S5 Text pEM’s performance dependence on track length. (PDF)

S6 Text Testing the pEM performance when assumptions are not satisfied. (PDF)

S7 Text Validating pEM assumptions for protein trajectories of Rho GTPase. (PDF)

S8 Text Determining the activation levels of Rho GTPase. (PDF)

S9 Text Supporting information references. (PDF)
