# Supplementary material for: Extracting Diffusive States of Rho GTPase in Live Cells: Towards In Vivo Biochemistry
Source: PLoS Comput Biol. 2015 Oct 29;11(10):e1004297. doi: 10.1371/journal.pcbi.1004297 (PMC4626024; doi:10.1371/journal.pcbi.1004297)
Supplement: S1 Data — (ZIP) [file pcbi.1004297.s001.zip › pEM GUI/README.rtf]

—————————————————————————————————————————————SUMMARY: This MATLAB graphical user interface allows the user to load a set of protein trajectories and run perturbation expectation-maximization to determine the number of diffusive states and determine the short-time diffusive properties of each state.  We have provided various sets of synthetic protein trajectories (Case&_@_#.mat, where & corresponds to the case number in the Table S1, @ corresponds to whether the static localization noise is constant (conts) or variable (var), and # corresponds to the number of protein trajectories in the dataset).  Each synthetic protein track begins on a different location of a square grid with a separation of 1 um for Case1 and 0.5 um for Cases 2 and 3.  The format of the protein trajectories that pEM requires to analyze is a cell array: X{1}, X{2}, … , X{M}, where M is the total number of particle tracks.  Within each cell, X{m} = [xpositions ypositions], where positions are given by a column vector.  (Note: positions for pEM analysis should be in um) Example: Case1_var_500.mat takes up to 15 minutes to run on a standard laptop; while Case1_const_1000.mat can take up to 30 minutes to run.  Running on a desktop with a powerful processor can certainly help to reduce these times.  To run pem_gui.m, copy “pem_gui” in the command prompt of MATLAB without the quotations.  Also provided is a MATLAB script called main_pEM.m to allow a user to modify the code to fit their needs. —————————————————————————————————————————————pem_gui.m - run down	1. Load Data - push button which prompts the user to select a mat file where the protein 	trajectories are stored.  This should be a cell array called X, where each element contains the 	2D protein trajectory.  Positions should be in um. (See Case1_500.mat for an example)	2. Filename - displays the filename of the selected file from Load DataMovie Parameters ->	3. Frame Duration (s) - user has to set the frame duration between protein positions	4. Exposure time (s) - user has to set the exposure time rEM parameters ->	5. No. of reinitializations - number of reinitializations to try.  The minimum value should be 1.  	Increasing this value results in a longer time to run.  The default value is set at 3.  This is because a very 	poor initialization may yield poor results regardless of the number of perturbation trials.pEM parameters ->	6. Min. Model Size - smallest model size to start pEM	7. Max. Model Size - largest model size to consider	8. No. of Perturbations - number of perturbation trials to test Increasing the number of 	perturbations may yield more accurate results. Hence, when analyzing actual experimental 	data, we suggest this value be change to 50-100.  However, the computational time will 	increase correspondingly.  	9. Max. iterations - maximum iterations to allow within a single EM run	10. Convergence - convergence is when the change in likelihood falls below this value.  Larger 	numbers lead to suboptimal results, while smaller numbers require longer time to converge.	11. Show Progress - plots the pEM parameter estimates at each iteration (will slow down 	computational time, so it is not recommended on a regular basis.)  	12. RUN - push button to run rEM/pEM 	13. Display Results - displays pEM results on command window	14. Display Posterior MSD - plots the posterior-weighted average of the time-averaged MSDs 	for each diffusive state (shown in a different color)	15. Display Posterior Tracks - plots all of the protein trajectories rendered with a color 	proportional to the posterior probability of being in each diffusive state.	16. Save Results - push button to save the results structure of pEM with the output filename. 	The output file is saved in a Results directory which is generated if it does not already exist.—————————————————————————————————————————————Output:Results structure:	results - array of structures for each model size            results(model size).structure elements:		    .numberOfStates - model size, i.e. number of states, K 		    .BIC - Bayesian information criterion score for the model size (scalar)		    .optimalD - optimal diffusion coefficients for each state (vector, 1xK)		    .optimalS - optimal static localization noise for each state (vector, 1xK)    		    .optimalP - optimal population fraction for each state (vector, 1xK)		    .optimalL - maximum likelihood value found (scalar)		    .posteriorProb - posterior probability of each protein track to be in a given diffusive state (matrix, MxK)		    .elapsedTime - elapsed time to run pEM for the given model size (scalar)—————————————————————————————————————————————List of functions within pEM directory:main.m: matlab script to execute pEM pEM.m: main function to execute the perturbation expectation-maximization procedureEM.m:rEM.m: main function to execute the reinitialization expectation-maximization procedureEM.m:Expectation.m: executes the expectation by calculating the posterior probabilitiesMaximization.m: executes the maximization for each particle trackLogDeterminant.m: calculates the log of the determinant with eigenvalue decompositionRandomInitialization.m: random parameter initialization based on the CDF of the diffusion coefficientCovarianceProperties.m: calculates the features of the empirical covariance matrix for each particle track.  TrackLengthParameters: determines the length of each particle track and finds unique track lengthsDisplayPosteriorTracks.m: displays the particle tracks as an image with a color proportional to the posterior probability for a given diffusive stateDisplayWeightedMSD.m: displays the time-averaged mean square displacement of all particle tracks each weighed by the posterior probability for a given diffusive state—————————————————————————————————————————————For further support, please contact:simon.mochrie@yale.edu 
