# Supplementary material for: Extracting Diffusive States of Rho GTPase in Live Cells: Towards In Vivo Biochemistry
Source: PLoS Comput Biol. 2015 Oct 29;11(10):e1004297. doi: 10.1371/journal.pcbi.1004297 (PMC4626024; doi:10.1371/journal.pcbi.1004297)
Supplement: S1 Table — Diffusivities, static localization noises, and population fractions used to generate synthetic protein trajectories are presented for case 1, case 2, and case 3. The columns represent the set of parameter values for each diffusive state. (PDF) [file pcbi.1004297.s002.pdf]

S1 Table. Simulation parameters for synthetic particle trajectories. Diffusivities, static localization noises, and population fractions used to generate synthetic protein trajectories are presented for case 1, case 2, and case 3. The columns represent the set of parameter values for each diffusive state.

		1	2	3	4	5	6	7
1	$D_k^{sim}(\mu m^2 s^{-1})$	0.01	0.3	1.2	2.8			
	π_k^{sim}	0.20	0.30	0.40	0.10			
	$\sigma_k^{sim}(\mu m)$	0.035	0.05	0.035	0.05			
2	$D_k^{sim}(\mu m^2 s^{-1})$	0.03	0.1	0.25	0.45			
	π_k^{sim}	0.20	0.10	0.40	0.30			
	$\sigma_k^{sim}(\mu m)$	0.035	0.05	0.035	0.05			
3	$D_k^{sim}(\mu m^2 s^{-1})$	10^{-8}	0.01	0.06	0.16	0.34	0.55	0.94
	π_k^{sim}	0.20	0.02	0.05	0.10	0.28	0.30	0.05
	$\sigma_k^{sim}(\mu m)$	0.03	0.035	0.04	0.05	0.04	0.05	0.06
