# Supplementary material for: Extracting Diffusive States of Rho GTPase in Live Cells: Towards In Vivo Biochemistry
Source: PLoS Comput Biol. 2015 Oct 29;11(10):e1004297. doi: 10.1371/journal.pcbi.1004297 (PMC4626024; doi:10.1371/journal.pcbi.1004297)
Supplement: S2 Table — D sim and π sim represent the parameters used to simulate 10,000 synthetic protein trajectories with motion blur and a constant static localization noise, σ sim = 0.05 μm, with diffusivities and population fractions given according to case 1 and case 2. The cumulative distribution function (CDF) parameter estimates are determined by applying a non-linear least squares fit to the cumulative square displacements, Δr 2 = Δx 2 + Δy 2, across the population of synthetic protein trajectories according to [30]: CDF(Δr2)=1-(∑k=1Kπke-Δr2/(2ρk)), where ρ k = 2D kΔt + 2σ 2 − 4RD kΔt, {D k} are the diffusion coefficients, and {π k} are the population fractions which satisfy the normalization, ∑k=1Kπk=1. Here, ρ k and π k are the free parameters and the normalization constraint for π k is enforced by replacing the population fraction of the Kth diffusive state, π k, with 1-∑i=1K-1πk. The fit is applied over the range 0 to ∞. D CDF and π CDF represent the CDF values from fitting the cumulative square displacements of the synthetic protein trajectories when using the simulated values as the initial guess and a known static localization noise (S3 Fig). D true and π true represent the CDF estimates when applied to the same protein trajectories, but without localization noise, and using the simulated values as the initial guess. Units of D, μm2s−1. (PDF) [file pcbi.1004297.s003.pdf]

S2 Table. CDF parameter estimates for synthetic particle trajectories. D^{sim} and π^{sim} represent the parameters used to simulate 10,000 synthetic protein trajectories with motion blur and a constant static localization noise, $\sigma^{sim} = 0.05$ μm, with diffusivities and population fractions given according to case 1 and case 2. The cumulative distribution function (CDF) parameter estimates are determined by applying a non-linear least squares fit to the cumulative square displacements, $\Delta r^2 = \Delta x^2 + \Delta y^2$, across the population of synthetic protein trajectories according to [30]: $CDF(\Delta r^2) = 1 - \left(\sum_{k=1}^{K} \pi_k e^{-\Delta r^2/(2\rho_k)}\right)$, where $\rho_k = 2D_k\Delta t + 2\sigma^2 - 4RD_k\Delta t$, $\{D_k\}$ are the diffusion coefficients, and $\{\pi_k\}$ are the population fractions which satisfy the normalization, $\sum_{k=1}^{K} \pi_k = 1$. Here, ρ_k and π_k are the free parameters and the normalization constraint for π_k is enforced by replacing the population fraction of the Kth diffusive state, π_k, with $1 - \sum_{i=1}^{K-1} \pi_k$. The fit is applied over the range 0 to ∞. D_{CDF} and π_{CDF} represent the CDF values from fitting the cumulative square displacements of the synthetic protein trajectories when using the simulated values as the initial guess and a known static localization noise (S3 Fig.). D_{true} and π_{true} represent the CDF estimates when applied to the same protein trajectories, but without localization noise, and using the simulated values as the initial guess. Units of D, μm^{2}s^{-1}.

	Case 1				Case 2			
D_{sim}	0.01	0.3	1.2	2.8	0.03	0.1	0.25	0.45
D_{CDF}	0.031	0.056	0.21	1.12	0.03	0.04	0.10	0.35
D_{true}	0.01	0.28	1.22	3.71	0.01	0.08	0.31	0.73
π_{sim}	0.2	0.3	0.4	0.1	0.2	0.1	0.4	0.3
π_{CDF}	0.08	0.19	0.39	0.35	0.06	0.13	0.35	0.46
π_{true}	0.37	0.24	0.27	0.12	0.39	0.16	0.27	0.18
