# Supplementary material for: Extracting Diffusive States of Rho GTPase in Live Cells: Towards In Vivo Biochemistry
Source: PLoS Comput Biol. 2015 Oct 29;11(10):e1004297. doi: 10.1371/journal.pcbi.1004297 (PMC4626024; doi:10.1371/journal.pcbi.1004297)
Supplement: S3 Table — (PDF) [file pcbi.1004297.s004.pdf]

S3 Table. Numbers of Rho protein trajectories used for pEM analysis.

	Number of cells	Number of trajectories
RhoA	5	7329
RhoA G14V	7	6246
RhoA F30L	4	6293
RhoA T19N	5	15969
RhoA HV	6	10304
RhoC	8	9104
RhoC G14V	3	7655
RhoC F30L	4	25074
RhoC T19N	2	6572
RhoC HV	4	10742
RhoC Chimera HV	4	14274
