# Supplementary figures and images for: Extracting Diffusive States of Rho GTPase in Live Cells: Towards In Vivo Biochemistry
Source: PLoS Comput Biol. 2015 Oct 29;11(10):e1004297. doi: 10.1371/journal.pcbi.1004297 (PMC4626024; doi:10.1371/journal.pcbi.1004297)

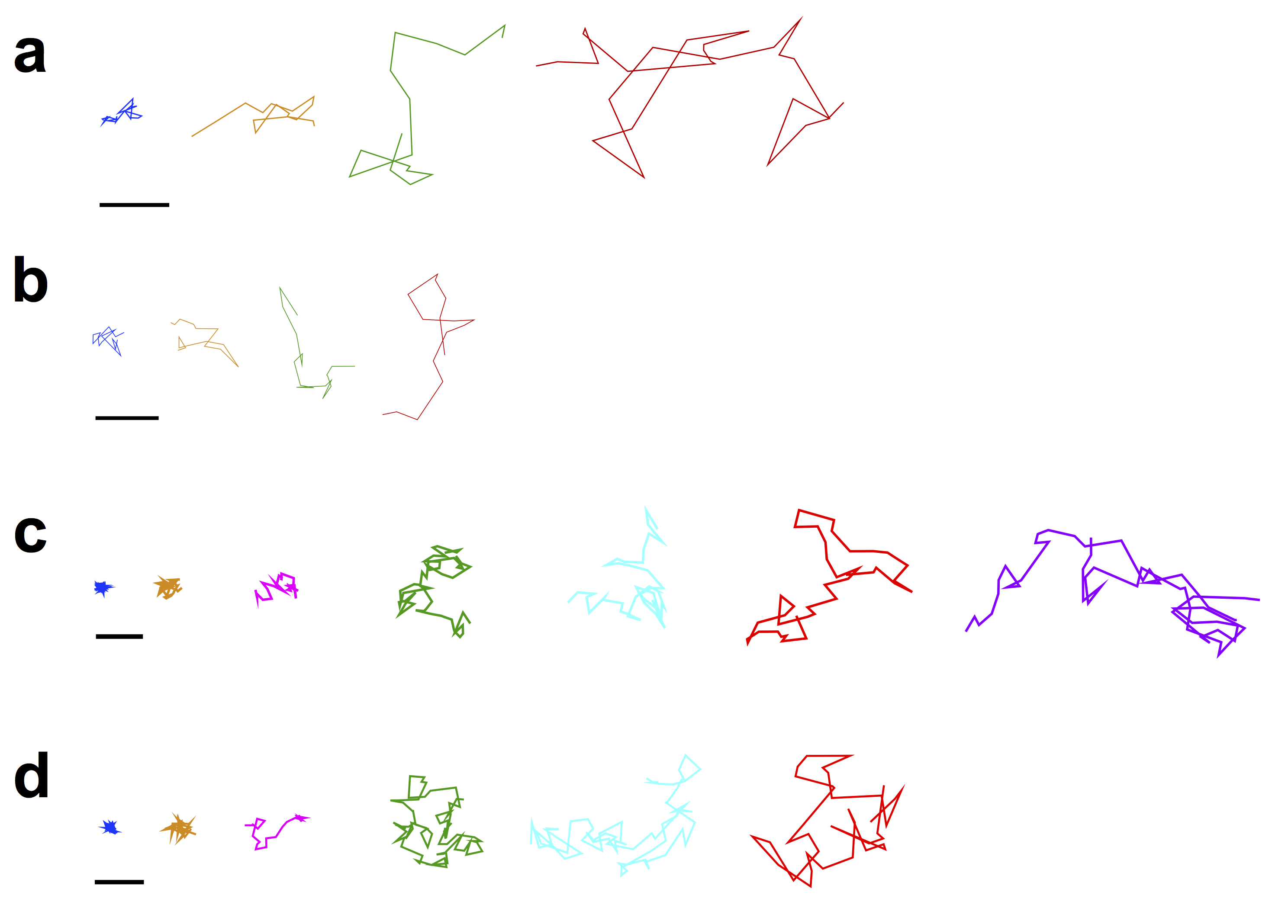

Supplement: S1 Fig — Example single protein trajectories for each diffusive state (shown in a different color) from synthetic protein trajectories given according to (a) case 1, (b) case 2, and (c) case 3. The underlying diffusivity of each diffusive state increases from left to right. (d) Shows example experimental RhoA trajectories based upon classification to each diffusive state. Protein trajectories, which had a posterior probability greater than 0.95 for the given diffusive state, were randomly selected for case 1 and case 2. Protein trajectories which have at least 40 steps and a posterior probability greater than 0.95 for the given diffusive state, were randomly selected for case 3 and for the experimental RhoA trajectories. The scale bar in each case represents 5 μm. (TIFF) [file pcbi.1004297.s005.tiff]

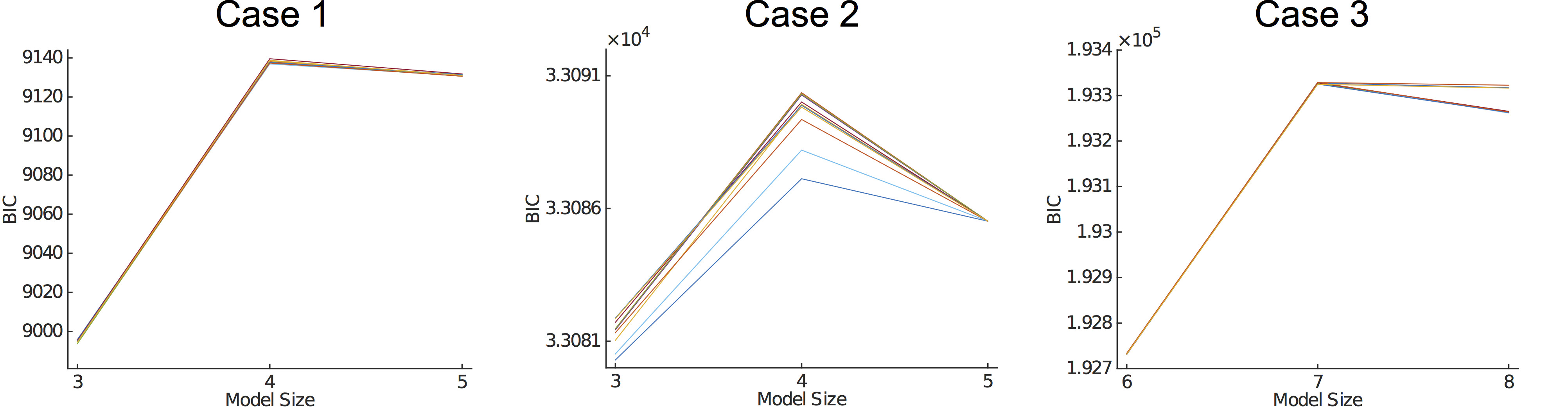

Supplement: S2 Fig — BIC scores for various model sizes, acquired by applying pEM to (left) 500 synthetic protein trajectories corresponding to case 1 with a constant static localization noise, (middle) 1,000 synthetic protein trajectories corresponding to case 2 with a constant static localization noise, and (right) 5,000 synthetic protein trajectories corresponding to case 3 with variable static localization noises. Each plot shows the BIC score from 10 different initial parameter values (each shown in a different color). The maximum BIC score is consistently found for a four diffusive state model for cases 1 and 2, and seven diffusive state model for case 3. Evidently since the variability of the BIC score is smaller than its change for different number of states for all 3 cases, the BIC is a reliable method for model selection in this case. (TIFF) [file pcbi.1004297.s006.tiff]

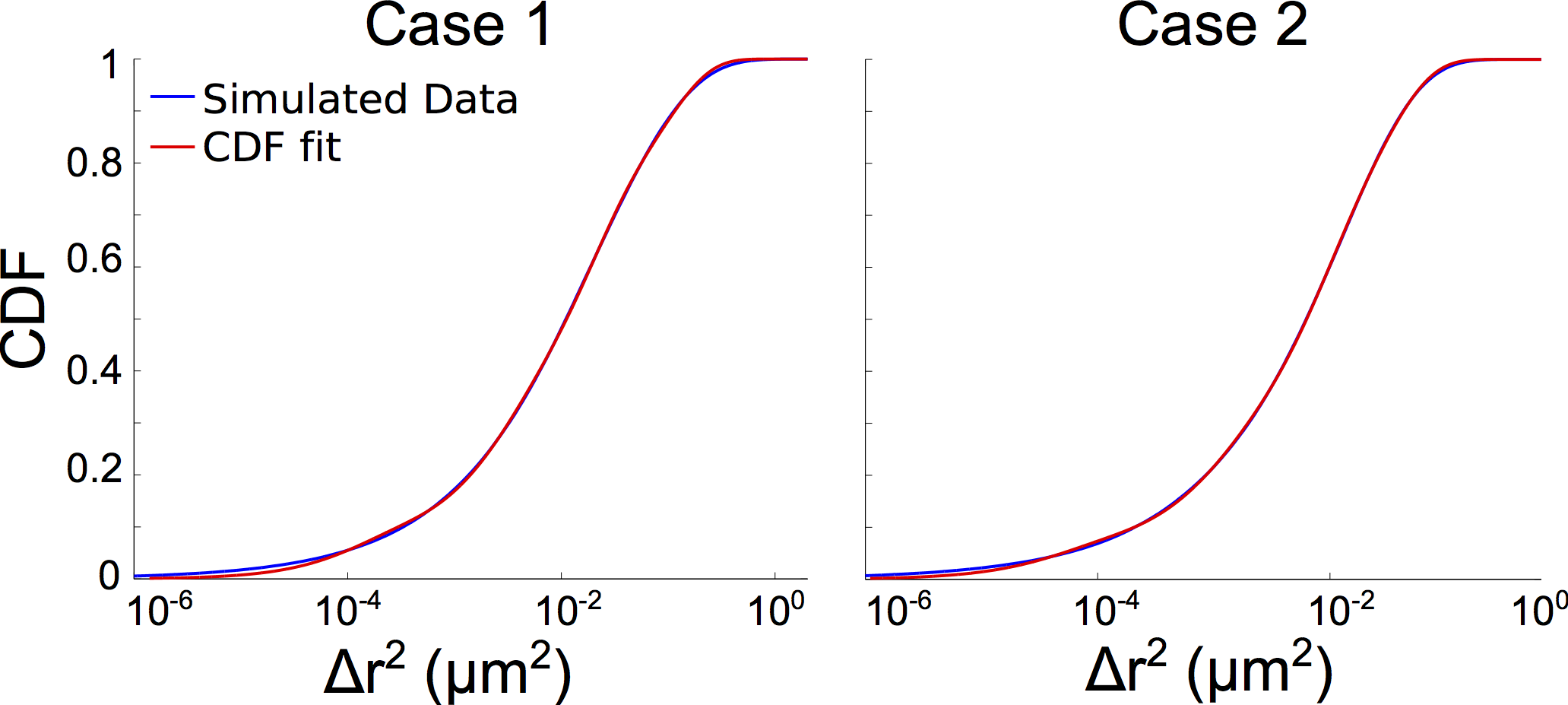

Supplement: S3 Fig — The cumulative distribution of the square displacements, determined from 10,000 synthetic protein trajectories, corresponding to case 1 (left) and case 2 (right), is shown as the blue curve, while the best fit CDF is shown as the red curve. The fitted parameters, namely the diffusivity, D k, and the population fraction, π k, for each diffusive state, k, are given in S2 Table. (TIFF) [file pcbi.1004297.s007.tiff]

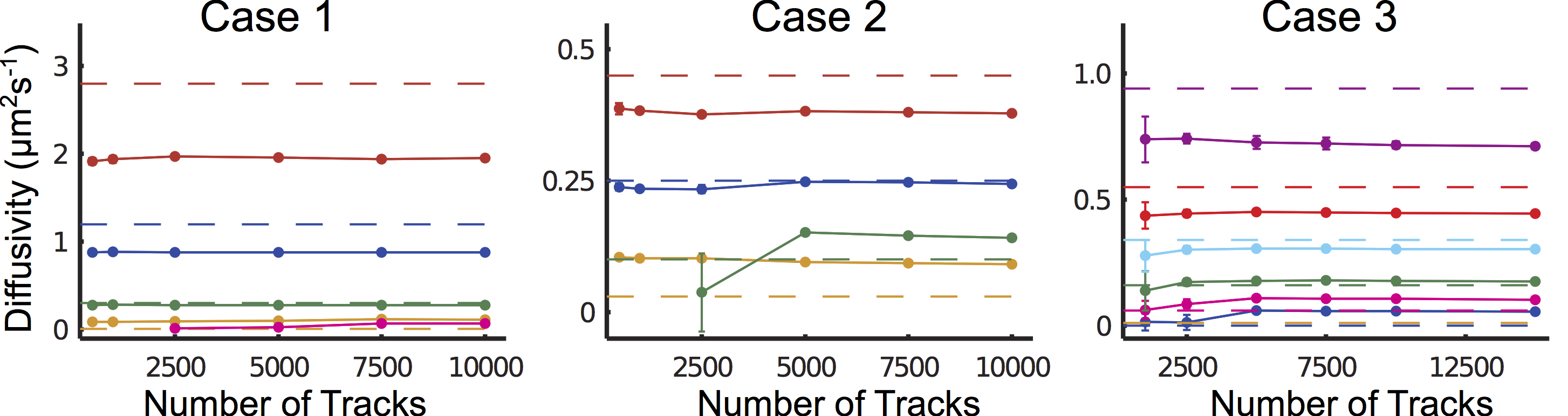

Supplement: S4 Fig — Solid circles are the uncorrected diffusivity estimates, determined using vbSPT, for various numbers of synthetic protein trajectories, corresponding to case 1 (left), case 2 (middle), and case 3 (right). Different colors correspond to different diffusive states. Each data point represents the average of five sets of synthetic protein trajectories and the error bars represent the standard deviation. Where the error bars are not visible, they are smaller than the symbol size. The solid lines are a guide-to-the-eye. The horizontal dashed lines represent the ground truth, i.e. the values of the diffusion coefficients that are input to the simulation. The color of each dashed line indicates the corresponding underlying diffusive state. vbSPT was implemented with the open-source software described in Ref. [32]. Since the vbSPT framework does not directly incorporate localization noise, the diffusivity estimates generated by vbSPT are biased, except at particular values of σ 2 and D, at which the effects of static and dynamic localization noise on-average cancel, i.e. for 2σ 2 − 4DRΔt ≈ 0. For diffusivities below this threshold value, the estimates exhibit a positive bias due to the dominant static localization noise. Above this threshold value, the estimates exhibit a negative bias due to the dominant dynamic localization noise. (TIFF) [file pcbi.1004297.s008.tiff]

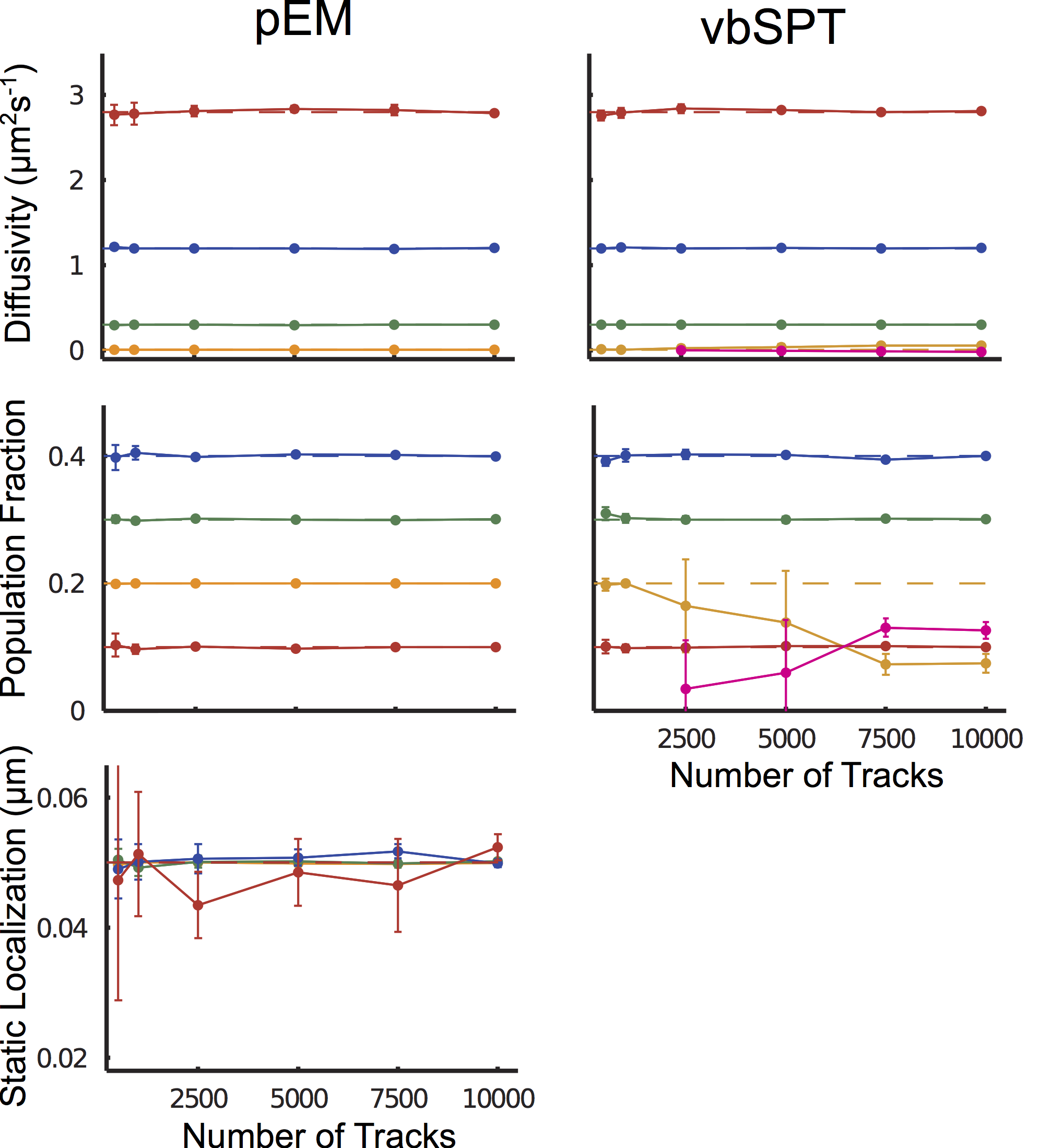

Supplement: S5 Fig — Diffusivity and population fraction estimates for various numbers of synthetic protein trajectories, corresponding to case 1, but with a constant static localization noise, σ = 0.05 μm, for pEM (left) and vbSPT (right). The static localization noise is assumed to be known for vbSPT, but must be determined by pEM (bottom-left). Each color corresponds to a different diffusive state. Each data point represents the average of five sets of synthetic protein trajectories and the error bars represent the observed standard deviations. The solid lines are guides-to-the-eye. The horizontal dashed lines represent the ground truth, i.e. the values input to the simulations. The color of each dashed line indicates the corresponding diffusive state. Because the diffusivity estimates generated by vbSPT are biased, in order to present the analyis of protein trajectories with localization noise based on vbSPT, we propose a post hoc correction to the diffusivity, determined by vbSPT, namely DkvbSPT, to mitigate the bias, namely Dk=DkvbSPTΔt-σ2(1-2R)Δt. In this figure and in S6 and S7 Figs, the displayed results obtained using vbSPT have had this correction applied. (TIFF) [file pcbi.1004297.s009.tiff]

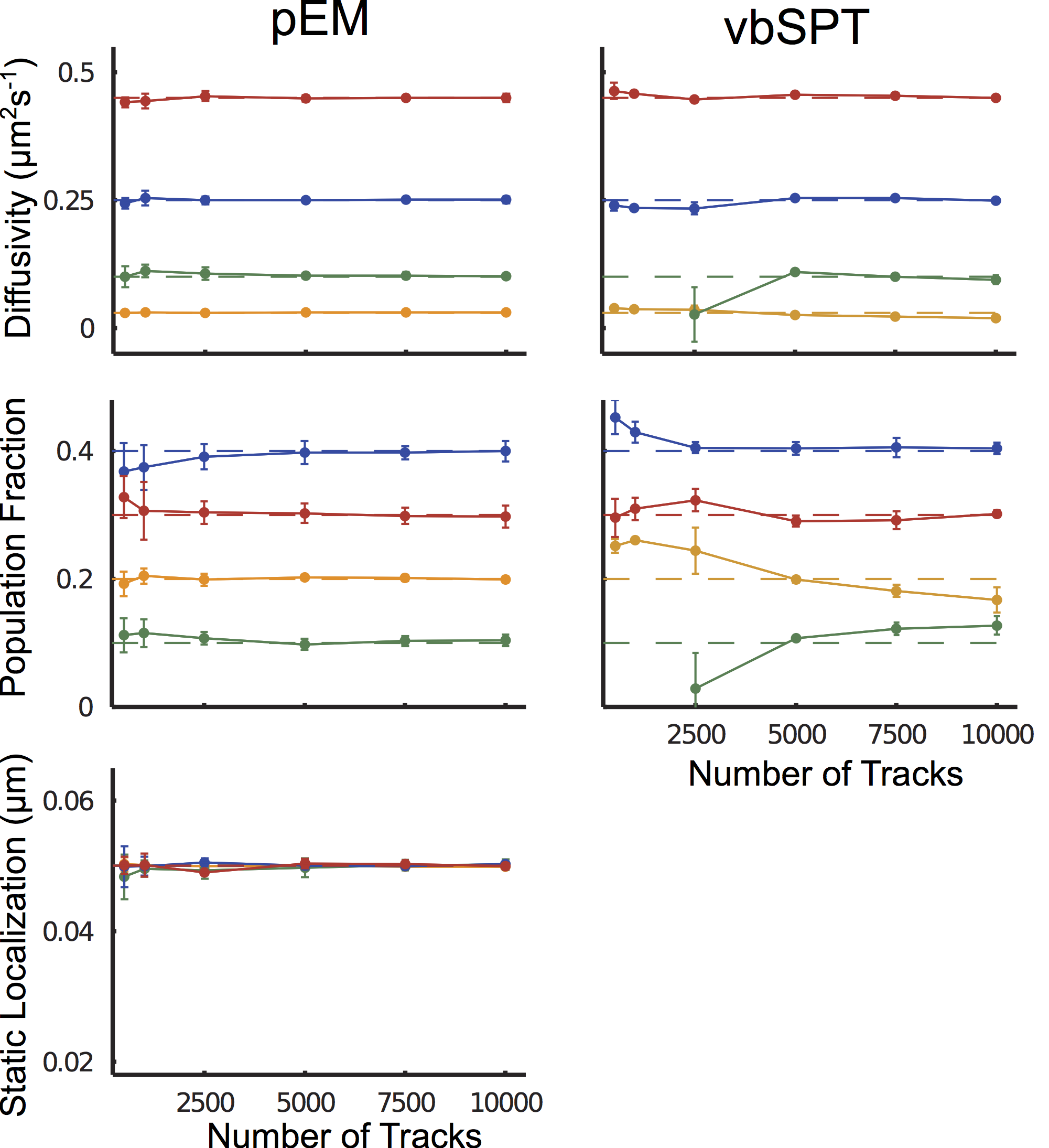

Supplement: S6 Fig — Diffusivity and population fraction estimates for various numbers of synthetic protein trajectories corresponding to case 2, but with a constant static localization noise, σ = 0.05 μm, for pEM (left) and vbSPT (right). The static localization noise is assumed to be known for vbSPT but must be determined by pEM (bottom-left). Each color corresponds to a different diffusive state. Each data point represents the average of five sets of synthetic protein trajectories and the error bars represent the observed standard deviations. The solid lines are guides-to-the-eye. The horizontal dashed lines represent the ground truth, i.e. the values input to the simulation. The color of each dashed line indicates the corresponding diffusive state. (TIFF) [file pcbi.1004297.s010.tiff]

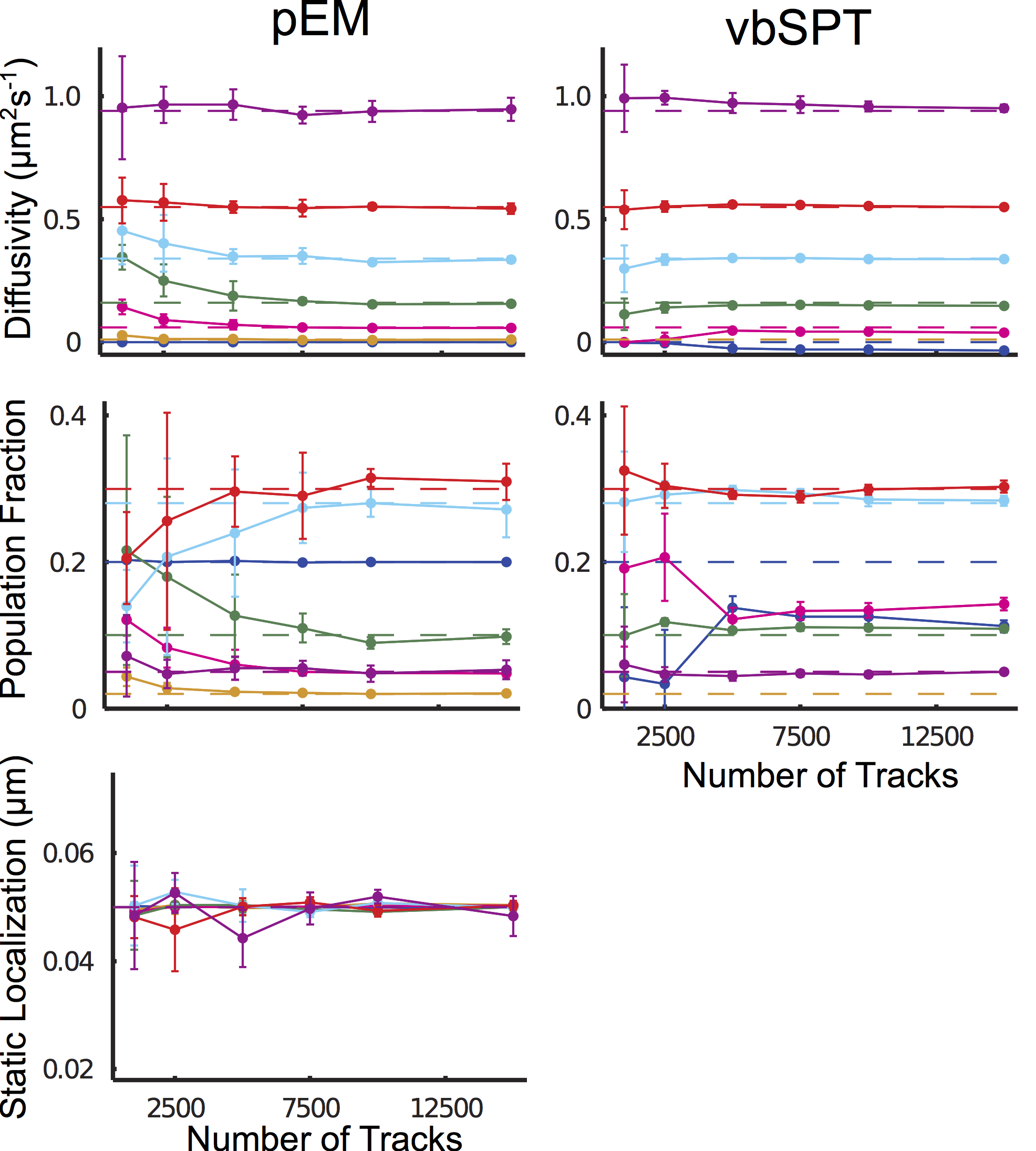

Supplement: S7 Fig — Diffusivity and population fraction estimates for various numbers of synthetic protein trajectories, corresponding to case 3, but with a constant static localization noise, σ = 0.05 μm, for pEM (left) and vbSPT (right). The static localization noise is assumed to be known for vbSPT but must be determined by pEM (bottom-left). Each color corresponds to a different diffusive state. Each data point represents the average of five sets of synthetic protein trajectories and the error bars represent the observed standard deviations. The solid lines are guides-to-the-eye. The horizontal dashed lines represent the ground truth, i.e. the values input to the simulation. The color of each dashed line indicates the corresponding diffusive state. (TIFF) [file pcbi.1004297.s011.tiff]

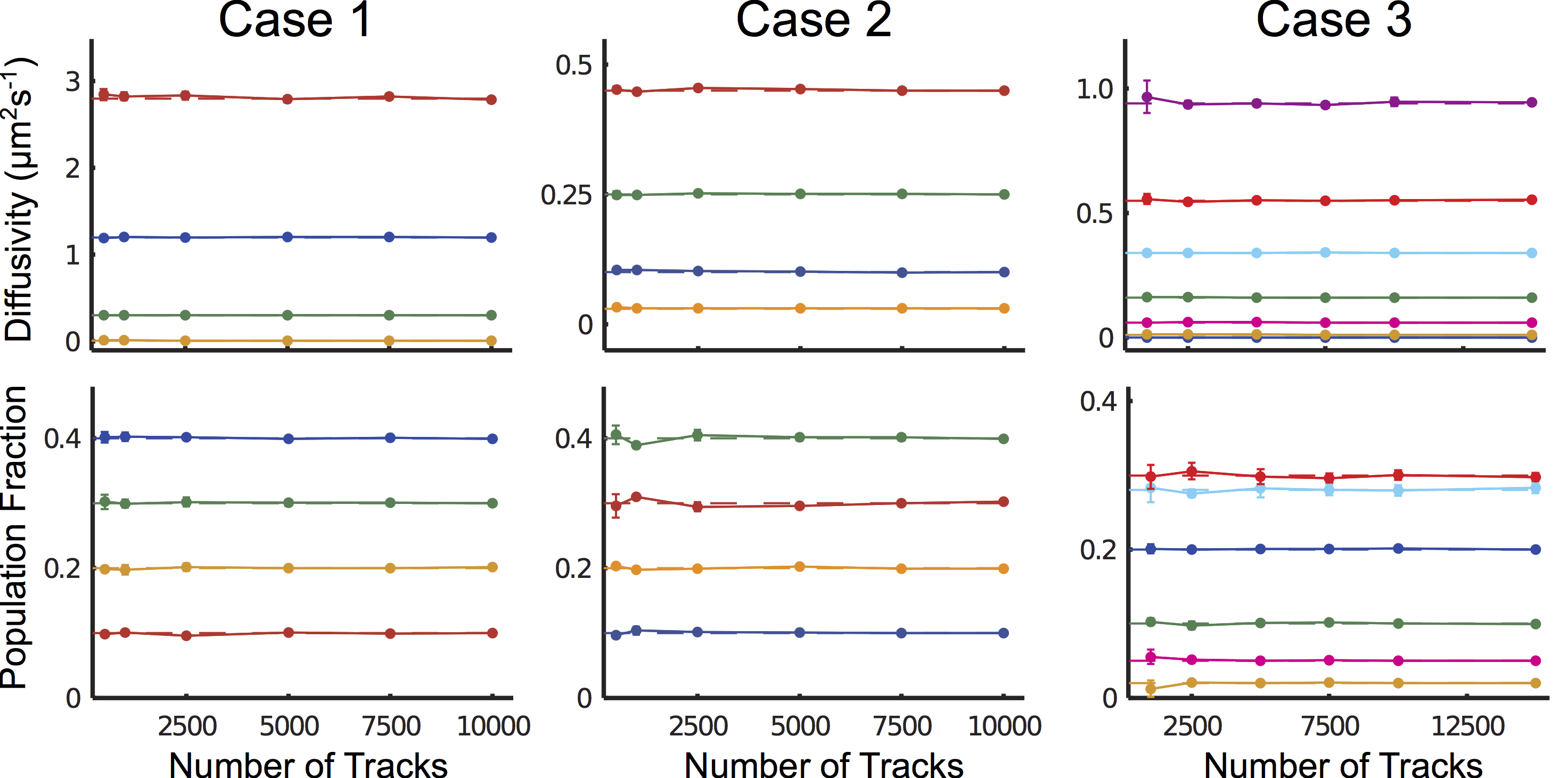

Supplement: S8 Fig — Diffusivities and population fractions, given by vbSPT applied to various numbers of synthetic protein trajectories without localization noise, corresponding to case 1 (left), case 2 (middle), and case 3 (right). Each color corresponds to a different diffusive state. Each data point represents the average of five sets of synthetic protein trajectories and the error bars represent the observed standard deviation. The solid lines are guides-to-the-eye. The horizontal dashed lines represent the ground truth, i.e. the values input to the simulation. The color of each dashed line indicates the corresponding diffusive state. (TIFF) [file pcbi.1004297.s012.tiff]

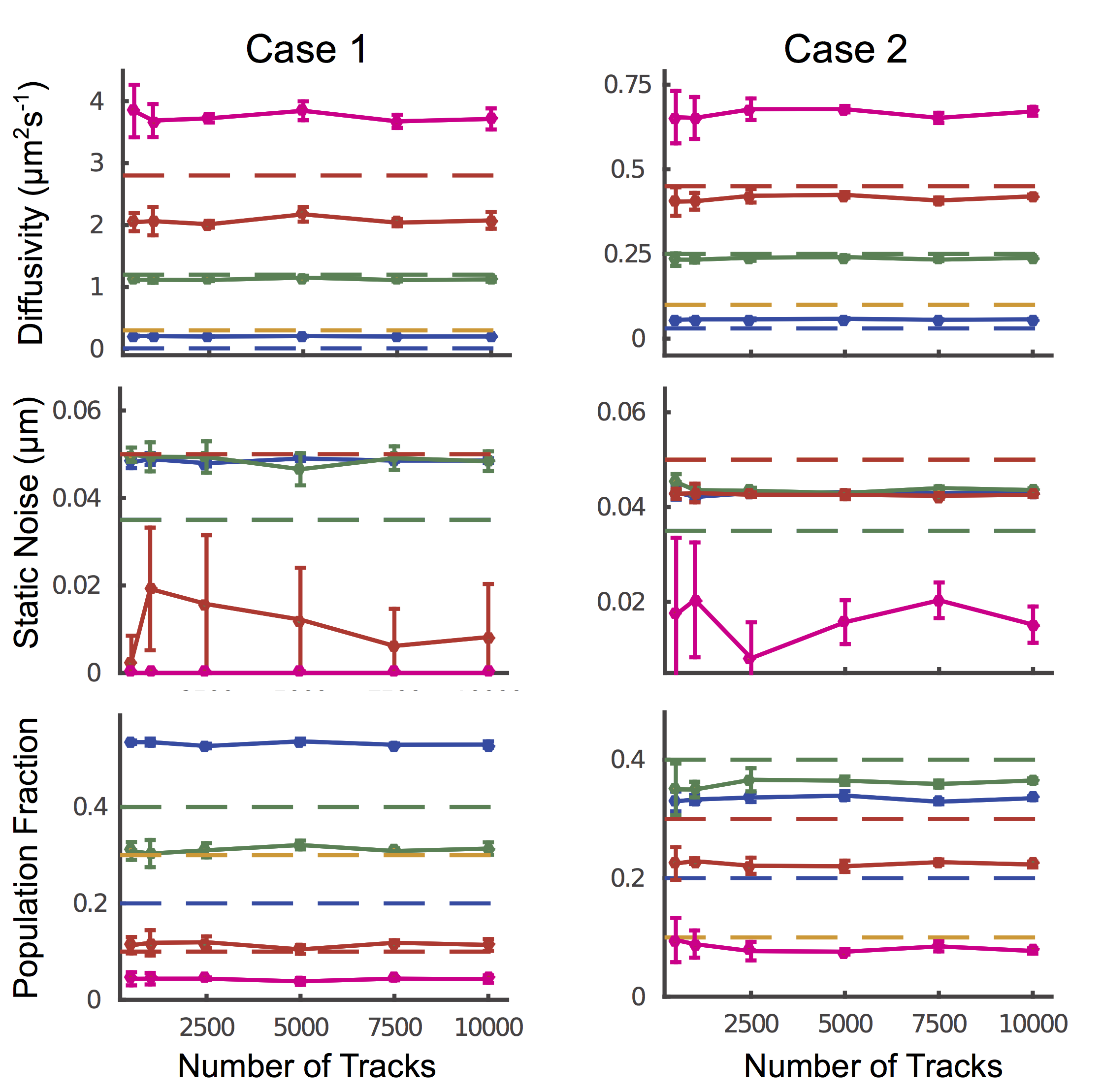

Supplement: S9 Fig — Diffusivity, static localization noise, and population fraction estimates for various numbers of synthetic protein trajectories, corresponding to case 1 (left) and case 2 (right) determined on the basis of k-means clustering applied to the diffusivity and static localization noise estimates for each protein trajectory [36]. Here, the covariance-based estimator was used to determine the diffusivity and static localization noise for each protein trajectory [49]. The k-means cluster for the correct model size was employed with a random initialization for each data set. Each data point represents the average of five sets of synthetic protein trajectories and the error bars represent the observed standard deviations. The solid lines are guides-to-the-eye. The horizontal dashed lines represent the ground truth, i.e. the values input to the simulation. The color of each dashed line indicates the simulated diffusive state. Each color of the k-means estimates corresponds to the closest diffusive state, except that one of the diffusive states found by k-means clustering is so far away from any actual diffusive state that it is given a different color (magenta). Because the performance of k-means clustering is so poor for case 1 and case 2, we did not test this method for case 3. (TIFF) [file pcbi.1004297.s013.tiff]

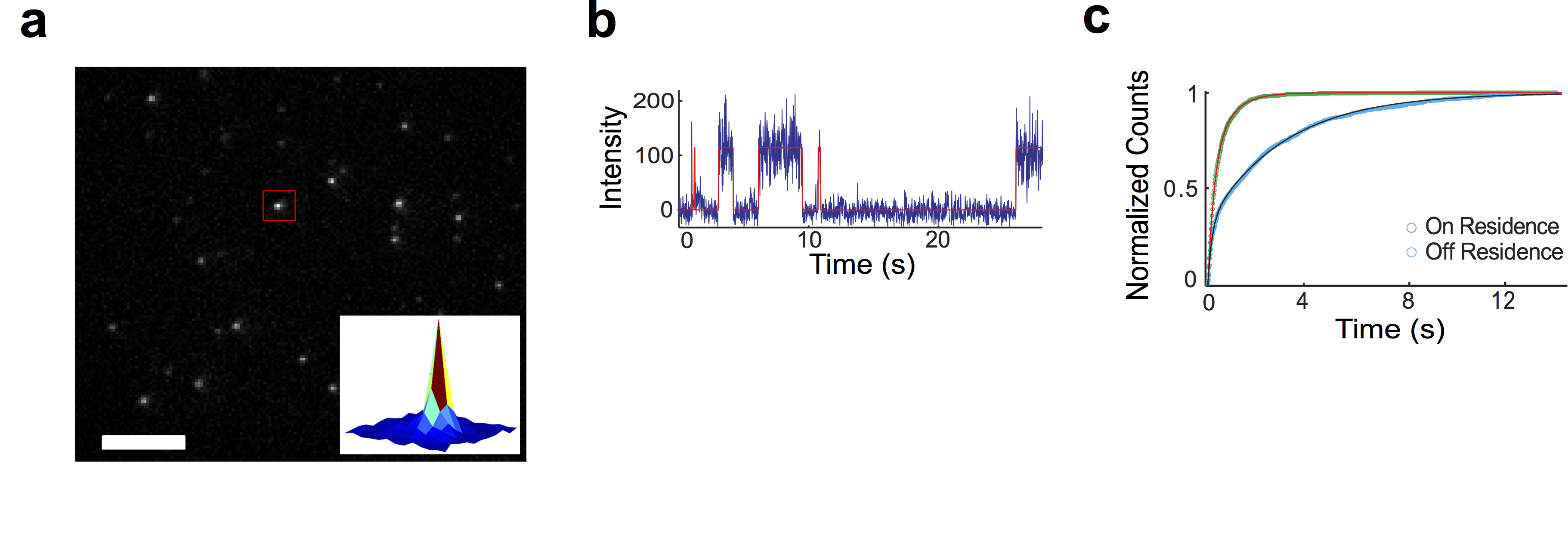

Supplement: S10 Fig — (a) Representative image from a pull-down assay of mEos2-RhoA. The inset shows the intensity profile of a single fluorophore within the red box. Scale bar is 10 μm. (b) Representative intensity time series taken from each point-spread function (blue) and the states found using hidden Markov models (red). (c) The cumulative distribution of residence times in the on state (green) and the off state (blue). The fits represent a two component cumulative exponential fits given by: y=1−wexp[−t/τ1]−(1−w)exp[−t/τ2], where w represent the fraction of component 1 decays, and τ 1 and τ 2 correspond to the characteristic residence times for component 1 and 2, respectively. The characteristic on-residence times were τ 1 = 0.26 s and τ 2 = 0.79 s with w = 0.6. The characteristic off-residence times were τ 1 = 0.17 s and τ 2 = 3.2 s with w = 0.33. (TIFF) [file pcbi.1004297.s014.tiff]

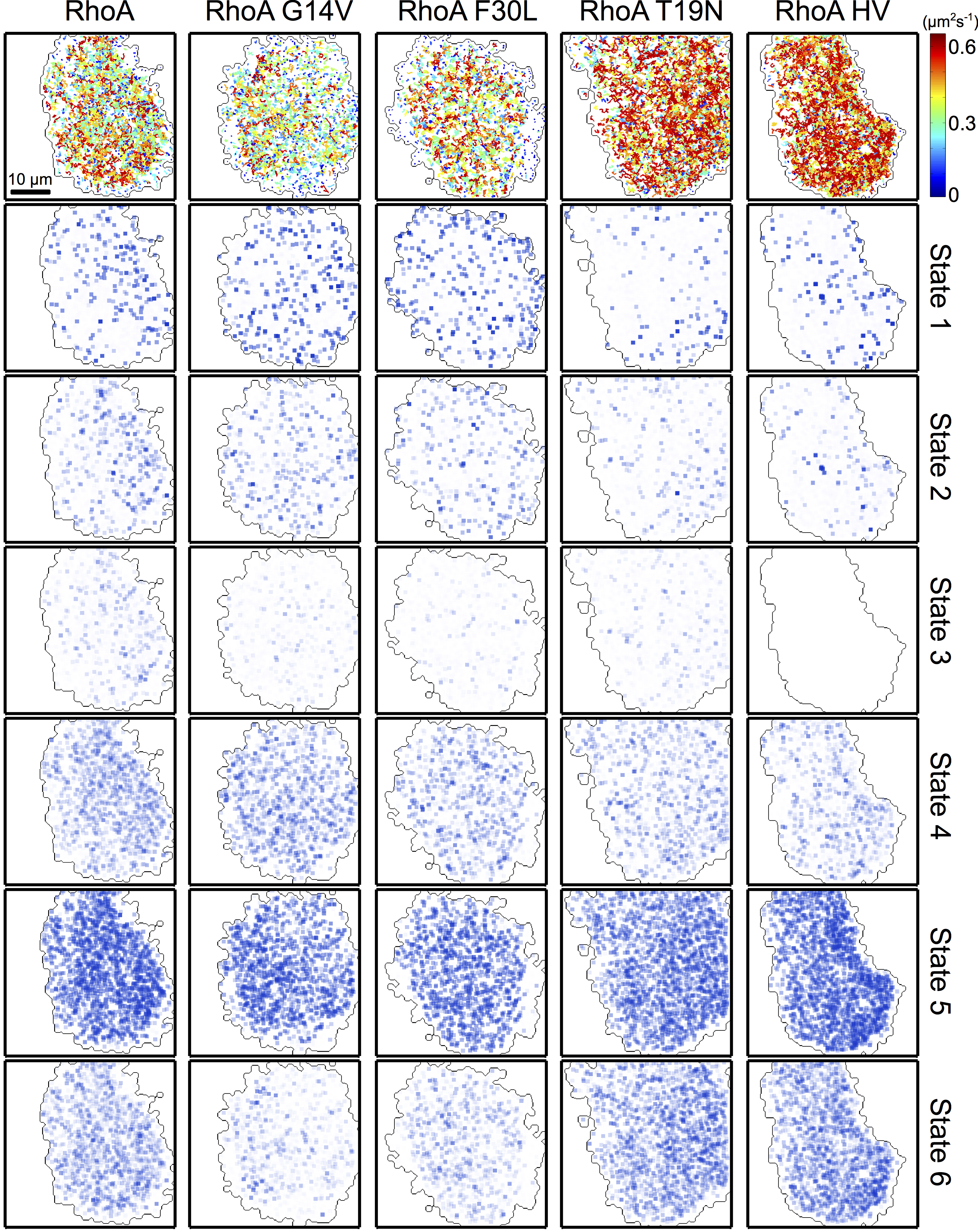

Supplement: S11 Fig — Each column corresponds to a representative cell of RhoA, RhoA G14V, RhoA F30L, RhoA T19N, and RhoA HV. For each of these cells, the top row shows 2,000 protein trajectories, rendered using a false color scale, with the color corresponding to the diffusivity estimates found using the CVE. The images shown in rows 2 through 7 constitute a posterior-weighted localization map for each diffusive state in each cell. Specifically, the centroid of each protein trajectory is rendered as a blue square with a transparency equal to one-third of the posterior probability that the track in question realizes that row’s diffusive state. Thus, dark blue regions represent regions inside the cell where there is a high posterior probability of finding one or more protein trajectories corresponding to the diffusive state in question. Pale and white regions represent regions of low and zero probability, respectively, to find a protein trajectory corresponding to that diffusive state. The cell boundary is outlined in black in each panel. Scale bar is 10 μm. (TIFF) [file pcbi.1004297.s015.tiff]

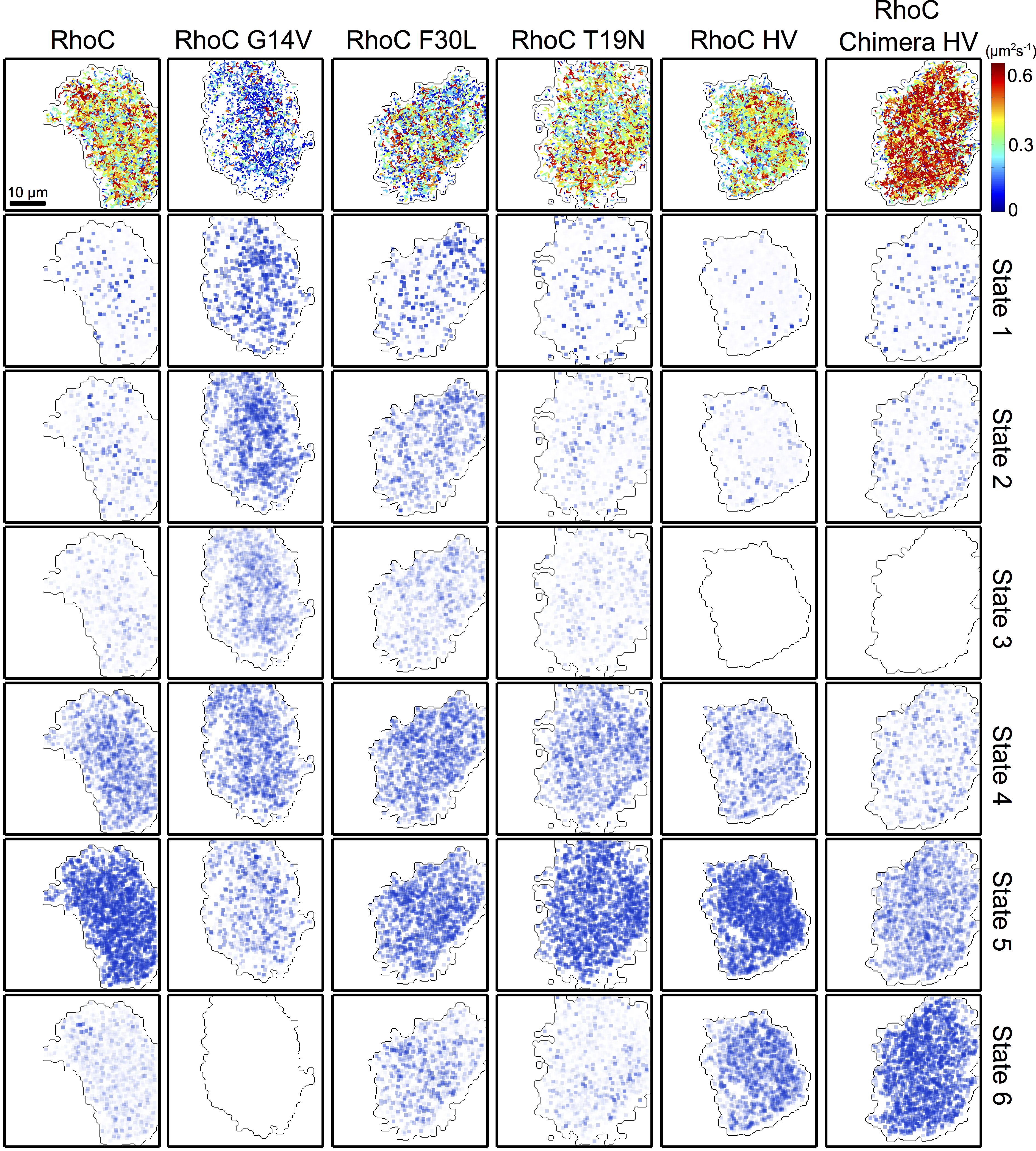

Supplement: S12 Fig — Each column corresponds to a representative cell of RhoC, RhoC G14V, RhoC F30L, RhoC T19N, RhoC HV, and RhoC Chimera HV. For each of these cells, the top row shows 2,000 protein trajectories, rendered using a false color scale, with the color corresponding to the diffusivity estimates found using the CVE. The images shown in rows 2 through 7 constitute a posterior-weighted localization map for each diffusive state in each cell. Specifically, the centroid of each protein trajectory is rendered as a blue square with a transparency equal to one-third of the posterior probability that the track in question realizes that row’s diffusive state. Thus, dark blue regions represent regions inside the cell where there is a high posterior probability of finding one or more protein trajectories corresponding to the diffusive state in question. Pale and white regions represent regions of low and zero probability, respectively, to find a protein trajectory corresponding to that diffusive state. The cell boundary is outlined in black in each panel. Scale bar is 10 μm. (TIFF) [file pcbi.1004297.s016.tiff]
