# Supplementary material for: Extracting Diffusive States of Rho GTPase in Live Cells: Towards In Vivo Biochemistry
Source: PLoS Comput Biol. 2015 Oct 29;11(10):e1004297. doi: 10.1371/journal.pcbi.1004297 (PMC4626024; doi:10.1371/journal.pcbi.1004297)
Supplement: S1 Text — (PDF) [file pcbi.1004297.s017.pdf]

S1 Text

Distribution of diffusion coefficients

The canonical physical model, underlying mean square displacement (MSD) analysis, for protein motion is that the protein undergoes diffusion as a result of its isotropic Brownian motion, which is described theoretically by Einstein's equation [67]:

$$\langle \rho(\Delta_m) \rangle = 4Dm\Delta t \; , \tag{Eq. S1}$$

where D is the diffusion coefficient, and m is an integer value corresponding to the number of lag time increments, Δt.

Although Eq. S1 describes the ensemble behavior of the diffusing protein, the ergodic property for normal diffusion specifies that the ensemble-averaged behavior is equivalent to the time-averaged behavior of the diffusing protein in the limit that the protein track length is infinite [68]. To calculate the time-averaged MSD (taMSD), it is common to augment the number of samples by drawing overlapping displacements from within a given protein trajectory. For a stationary sequence of T two-dimensional (2D) protein positions, $\mathbf{r} = \{x_t, y_t\}$ for $t = 1$ through T, each separated one from the next by a time, Δt, the overlapping taMSD is calculated according to [26, 28]:

$$\overline{\delta(\Delta_m, T)} = \frac{1}{T - \Delta_m} \sum_{t=1}^{T-\Delta_m} \left(\mathbf{r}(t + \Delta_m) - \mathbf{r}(t)\right)^2$$

where $\overline{\delta(\Delta_m, T)}$ is the taMSD for the mth time lag, $\Delta_m = m\Delta t$ and the bar on top of $\delta(\Delta_m, T)$ is used to distinguish the time average.

In analyzing individual protein trajectories via time-averages, we make the underlying assumption that the governing motion is ergodic. Hence, the time-averaged motions are representative of the ensemble behavior. However, due to the stochastic nature of diffusion, the equivalence only holds in the limit of an infinitely-long protein track [69]. Thus, when the ergodicity condition is satisfied, there is no variability in the diffusion coefficients estimates between protein trajectories. Experimental protein trajectories, however, have finite length. As a consequence, the ergodicity condition cannot be satisfied, ultimately resulting in a scatter of the taMSD about the ensemble-averaged MSD [68]. By plotting the taMSD calculated from synthetic protein trajectories *without* localization noise, we illustrate that the level of scatter increases with decreasing trajectory length (see S13 Fig.). As expected for normal diffusion, the average over all taMSD curves recovers ergodicity [68], i.e. $\langle \overline{\delta(\Delta_m, T)} \rangle = \langle \rho(\Delta_m) \rangle$, regardless of the length of the protein trajectories.

The progressively increasing scatter of the taMSD for higher time lags renders the determination of the underling diffusion mode difficult, especially for short protein trajectories. For instance, the shape of the taMSD curves for 15 steps and 30 steps exhibit significant deviations from a linear behavior, which may be mistaken for confined, driven, or anomalous diffusion. Thus, applying an unweighted least squares fit for each of these diffusion models inevitably results in artifacts. The reduced statistics from shorter protein trajectories renders the taMSD unreliable for classification for macroscopic diffusion modes. Thus, it is common to focus on the short-time diffusive behaviors, which models diffusion at timescales prior to interactions with the environment which may cause deviations from a linear behavior in the taMSD. For brevity, we drop the short-time prefix in the remainder of this Text.

S13 Fig. also displays the probability distributions of the diffusivity estimates for various track lengths calculated using only the first time lag of the taMSD, which is optimal for protein trajectories without localization noise [70]. When the protein trajectories are long, the distribution of diffusivities is narrow and the mode of the distribution is centered near the ensemble diffusion coefficient. That is to say, there is minimal variability in the diffusivity estimates between protein trajectories. As the protein track lengths become shorter, however, ergodicity is further broken, resulting in a broadening of the distribution and an

S13 Fig. Ergodicity breaking due to limited statistics. (a-d) Representative plot of 100 taMSD traces for the first 12 time lags from synthetic protein trajectories *without* localization noise with $D = 0.3$ μm^{2}s^{-1} and a track length of (a) 15, (b) 50, (c) 100, and (d) 500 steps. (e) Shows the probability distribution of the diffusivities estimated from the first time lags of the taMSD. Each distribution was generated by analyzing 2,000 "noiseless" synthetic protein trajectories with $D = 0.3$ μm^{2}s^{-1} for various track lengths (shown in a different color). The probability distributions in (e) were generated by building a histogram of the diffusivity estimates and normalizing each bin with the total number of protein trajectories.

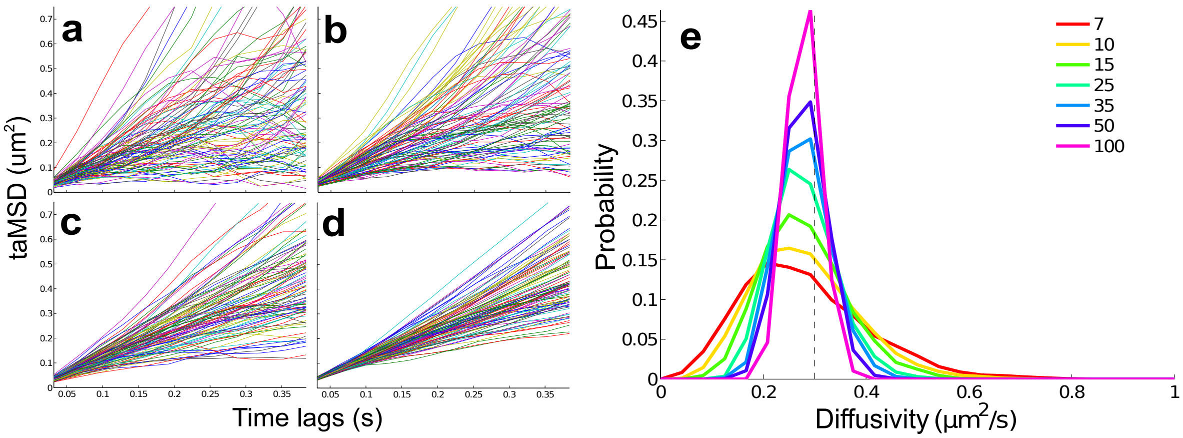

increased positive skew, so that the mode is further suppressed from the mean. The mean over the entire distribution, however, remains an unbiased estimate of the ensemble diffusion coefficient, irrespective of the protein track lengths.

Localization noise serves to obscure the exact positions of the diffusing protein. Thus, analysis of synthetic protein trajectories *with* localization noise inevitably results in a lower accuracy, compared to the analysis of similar synthetic protein trajectories *without* localization noise. Here, at least two MSD time lags are required to determine the two unknowns, namely the diffusion coefficient and the static localization noise. Even when the static localization noise is known, using only the first time lag of the MSD is not optimal because of the correlations in the nearest-neighbor displacements, that result from localization noise. However, a nearly optimal estimation of the diffusion coefficient and static localization noise for individual protein trajectories undergoing normal diffusion can be achieved with the covariance-based estimator (CVE) of Ref. [49].

We explore the impact of localization noise on the CVE-generated distribution of diffusivities by simulating protein trajectories with various levels of localization noise while maintaining a constant diffusion coefficient and track length of $D^{sim} = 0.3$ μm^{2}s^{-1} and $N^{sim} = 15$, respectively. S14 Fig. illustrates that the distribution of diffusivities broadens as the level of static localization noise increases. Nonetheless, the mean over the entire distribution, including any negative diffusivity estimates, continues to yield an unbiased estimate of the diffusion coefficient, irrespective of the value of the localization noise.

In general, the diffusion coefficient distributions are well-defined, in the sense that analyzing additional protein trajectories with the same properties, namely D^{sim}, N^{sim}, and σ^{sim}, does not produce more accurate estimates, *i.e.* the shape of the distributions remain unchanged as additional tracks are included. This feature of the probability distributions clearly implies that each diffusivity estimate is not variable as a result of experimental measurement errors, but rather the variation arises from noise inherent to the process under study in accordance with the Cramer-Rao lower bound [35]. Thus, as a consequence of ergodicity breaking, the diffusivity estimates can be understood as a random variable drawn from a

S14 Fig. Effect of localization noise on diffusivity distributions. The probability distribution of the diffusivities by applying CVE analysis on 2,000 "noisy" synthetic protein trajectories with $D = 0.3$ $\mu m^2 s^{-1}$ and a track length $N = 15$, for four values of the static localization noise, each shown in a different color. The probability distributions were generated by building a histogram of the diffusivity estimates and normalizing each bin with the total number of protein trajectories.

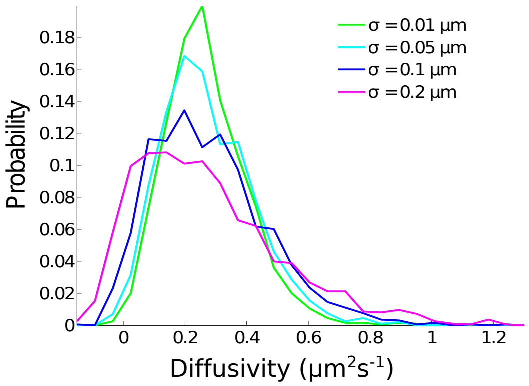

well-defined distribution [71], whose shape depends on the underlying diffusivity, mean protein track length, and static localization noise. Because the underlying diffusivity is an intrinsic property of the protein/cell system, improving the accuracy of diffusion coefficient estimates can only be achieved by analyzing longer protein trajectories and/or by reducing the level of localization noise.
