# Supplementary material for: Extracting Diffusive States of Rho GTPase in Live Cells: Towards In Vivo Biochemistry
Source: PLoS Comput Biol. 2015 Oct 29;11(10):e1004297. doi: 10.1371/journal.pcbi.1004297 (PMC4626024; doi:10.1371/journal.pcbi.1004297)
Supplement: S2 Text — (PDF) [file pcbi.1004297.s018.pdf]

S2 Text

Derivation of likelihood framework for diffusive states

The likelihood of observing an individual protein trajectory with N one-dimensional (1D) displacements, $\Delta\mathbf{x} = \{\Delta x(n)\}_{n=1}^{N}$, separated by Δt, in a diffusive state with a covariance matrix, $\boldsymbol{\Sigma}$, is given by a multivariate Gaussian distribution: [34]

$$P(\Delta\mathbf{x}|\boldsymbol{\Sigma}) = \frac{1}{(2\pi)^{N/2}|\boldsymbol{\Sigma}|^{1/2}} \exp\left[-\frac{1}{2}\Delta\mathbf{x}^T\boldsymbol{\Sigma}^{-1}\Delta\mathbf{x}\right],$$

where $\Delta\mathbf{x}^T$ is the transpose, $|\boldsymbol{\Sigma}|$ is the determinant, and $\boldsymbol{\Sigma}^{-1}$ is the inverse. Explicitly, the covariance matrix for a protein undergoing normal diffusion is given by: [34]

$$\boldsymbol{\Sigma}_{ij} = \begin{cases} 2D\Delta t + 2\sigma^2 - 4RD\Delta t & i = j \\ -\sigma^2 + 2RD\Delta t & i = j \pm 1 \\ 0 & \text{otherwise} \end{cases}$$

where D is the diffusion coefficient, σ is the static localization noise, and R is the motion blur coefficient [19], which depends on the shutter state during the camera integration time. For a shutter that is open throughout Δt, as we assume in this paper, $R = \frac{1}{6}$.

Thus the complete likelihood of observing M protein trajectories, $\Delta\hat{\mathbf{x}} = \{\Delta\mathbf{x}_m\}_{m=1}^{M}$, in this same diffusive state is given by the product of the individual protein trajectory likelihoods, according to $P(\Delta\hat{\mathbf{x}}|\boldsymbol{\Sigma}) = \prod_{m=1}^{M} P(\Delta\mathbf{x}_m|\boldsymbol{\Sigma})$. However, this assumes that all of the protein trajectories are homogeneous, sharing the same covariance matrix.

We can extend the likelihood of individual protein trajectories to include multiple diffusive states with a Gaussian mixture model [36]. When there are K diffusive states each characterized by a different $\boldsymbol{\Sigma}_k$, the formulation for the complete likelihood for the a population of protein trajectories is simplified by introducing a hidden variable, $\mathbf{z}$, which corresponds to an indicator of the underlying diffusive state. $\mathbf{z}$ is a K-dimensional binary random variable which has one element equal to 1 corresponding to the underlying diffusive state and the other elements are equal to 0, $\mathbf{z} = \{z_m(k)\}_{k=1}^{K}$.

In this representation, the likelihood distribution of the mth protein trajectory can be given by marginalizing the joint distribution, $P(\Delta\mathbf{x}_m, \mathbf{z}_m|\hat{\boldsymbol{\Sigma}})$, over the hidden variable, $\mathbf{z}$, according to:

$$\begin{aligned} P(\Delta\mathbf{x}_m|\hat{\boldsymbol{\Sigma}}) &= \sum_{z=1}^{K} P(\Delta\mathbf{x}_m, \mathbf{z}_m|\hat{\boldsymbol{\Sigma}}) \\ &= \sum_{z=1}^{K} P(\mathbf{z}_m|\hat{\boldsymbol{\Sigma}})P(\Delta\mathbf{x}_m|\mathbf{z}_m, \hat{\boldsymbol{\Sigma}}), \end{aligned} \qquad \text{(Eq. S2)}$$

where $\hat{\boldsymbol{\Sigma}} = \{\boldsymbol{\Sigma}_k\}_{k=1}^{K}$.

For protein trajectory m, we define the probability of a particular diffusive state is given by $P(z_m(k) = 1) = \pi_k$, which we call the population fraction with properties $0 \leq \pi_k \leq 1$ and normalization $\sum_{k=1}^{K} \pi_k = 1$. Thus, the probability over the hidden variables across all states given the $P(\mathbf{z}_m|\hat{\boldsymbol{\Sigma}})$, can be expressed in the form:

$$P(\mathbf{z}_m|\hat{\boldsymbol{\Sigma}}) = P(\mathbf{z}_m) = \prod_{k=1}^{K} \pi_k^{z_m(k)}. \qquad \text{(Eq. S3)}$$

Since the likelihood of $\Delta\mathbf{x}_m$ given a particular value for $\mathbf{z}_m$ is the same as the likelihood for an individual protein trajectory, *i.e.* $P(\Delta\mathbf{x}_m|z_m(k) = 1, \hat{\boldsymbol{\Sigma}}) = P(\Delta\mathbf{x}_m|\boldsymbol{\Sigma}_1)$, the full conditional probability can then be written in the form:

$$P(\Delta\mathbf{x}_m|\mathbf{z}_m, \hat{\boldsymbol{\Sigma}}) = \prod_{k=1}^{K} \left[P(\Delta\mathbf{x}|\boldsymbol{\Sigma}_k)\right]^{z_m(k)}. \qquad \text{(Eq. S4)}$$

Substituting Eq. S3 and Eq. S4 into Eq. S2, the marginal distribution of $\Delta\mathbf{x}_m$ becomes:

$$\begin{aligned}
P(\Delta\mathbf{x}_m|\hat{\boldsymbol{\Sigma}}) &= \sum_{k=1}^{K} P(\mathbf{z}_m(k)|\hat{\boldsymbol{\Sigma}})P(\Delta\mathbf{x}_m|\mathbf{z}_m(k),\hat{\boldsymbol{\Sigma}}) \\
&= \sum_{k=1}^{K}\left(\prod_{k=1}^{K}\pi_k^{z_m(k)}\right)\left(\prod_{k=1}^{K}[P(\Delta\mathbf{x}_m|\boldsymbol{\Sigma}_k)]^{z_m(k)}\right) \\
&= \sum_{k=1}^{K}\pi_k P(\Delta\mathbf{x}_m|\boldsymbol{\Sigma}_k).
\end{aligned}$$

Thus, the marginal distribution of $\Delta\mathbf{x}_m$ is a mixture of K multivariate Gaussians of individual protein trajectory likelihoods for each diffusive state. For M protein trajectories, the complete likelihood is given with a product of these Gaussian mixtures for each protein trajectory:

$$\mathcal{L}(\Delta\hat{\mathbf{x}}|\hat{\boldsymbol{\pi}},\hat{\boldsymbol{\Sigma}}) = \prod_{m=1}^{M}\sum_{k=1}^{K}\pi_k P(\Delta\mathbf{x}_m|\boldsymbol{\Sigma}_\mathbf{k}).$$

where $\hat{\boldsymbol{\pi}} = \{\pi_k\}_{k=1}^{K}$. Equivalently, the complete log-likelihood function:

$$\ln\mathcal{L}(\Delta\hat{\mathbf{x}}|\hat{\boldsymbol{\pi}},\hat{\boldsymbol{\Sigma}}) = \sum_{m=1}^{M}\ln\left\{\sum_{k=1}^{K}\pi_k P(\Delta\mathbf{x}_m|\boldsymbol{\Sigma}_k)\right\}.$$

Derivation of D_k and σ_k

To find the maximization relations for D_k and σ_k, we first maximize the log-likelihood (Eq. 1) with respect to $\boldsymbol{\Sigma}_k$, according to $\frac{\partial \ln\mathcal{L}}{\partial\boldsymbol{\Sigma}_k} = 0$.

$$\begin{aligned}
0 &= \frac{\partial}{\partial\boldsymbol{\Sigma}_k}\ln\mathcal{L}(\Delta\hat{\mathbf{x}}|\hat{\boldsymbol{\pi}},\hat{\boldsymbol{\Sigma}}) \\
&= \frac{\partial}{\partial\boldsymbol{\Sigma}_k}\sum_{m=1}^{M}\ln\left\{\sum_{k=1}^{K}\pi_k P(\Delta\mathbf{x}_m|\boldsymbol{\Sigma}_k)\right\} \\
&= \sum_{m=1}^{M}\frac{\pi_k\frac{\partial}{\partial\boldsymbol{\Sigma}_k}P(\Delta\mathbf{x}_m|\boldsymbol{\Sigma}_k)}{\sum_{j=1}^{K}\pi_j P(\Delta\mathbf{x}_m|\boldsymbol{\Sigma}_j)} \\
&= \sum_{m=1}^{M}\frac{\pi_k P(\Delta\mathbf{x}_m|\boldsymbol{\Sigma}_k)}{\sum_{j=1}^{K}\pi_j P(\Delta\mathbf{x}_m|\boldsymbol{\Sigma}_j)}\left(-\frac{1}{2}\right)\left[\boldsymbol{\Sigma}_k^{-1} - \boldsymbol{\Sigma}_k^{-1}\Delta\mathbf{x}_m\Delta\mathbf{x}_m^T\boldsymbol{\Sigma}_k^{-1}\right] \\
&= \sum_{m=1}^{M}\gamma_{mk}\left[\boldsymbol{\Sigma}_k^{-1} - \boldsymbol{\Sigma}_k^{-1}\Delta\mathbf{x}_m\Delta\mathbf{x}_m^T\boldsymbol{\Sigma}_k^{-1}\right] \\
\sum_{m=1}^{M}\gamma_{mk}\boldsymbol{\Sigma}_k &= \sum_{m=1}^{M}\gamma_{mk}\Delta\mathbf{x}_m\Delta\mathbf{x}_m^T \\
M_k\boldsymbol{\Sigma}_k &= \sum_{m=1}^{M}\gamma_{mk}\Delta\mathbf{x}_m\Delta\mathbf{x}_m^T \\
\boldsymbol{\Sigma}_k &= \frac{1}{M_k}\sum_{m=1}^{M}\gamma_{mk}\Delta\mathbf{x}_m\Delta\mathbf{x}_m^T, \qquad \text{(Eq. S5)}
\end{aligned}$$

where $\gamma_{mk} = \frac{\pi_k P(\Delta\mathbf{x}_m|\mathbf{\Sigma}_k)}{\sum_{j=1}^{K}\pi_j P(\Delta\mathbf{x}_m|\mathbf{\Sigma}_j)}$, and $M_k = \sum_{m=1}^{M}\gamma_{mk}$. Interestingly, γ_{mk} follows directly from Bayes' theorem, given by:

$$\begin{aligned}
\gamma_{mk} &= P(\mathbf{z}_m(k) = 1|\Delta\mathbf{x}_m, \hat{\mathbf{\Sigma}}) \\
&= \frac{P(\mathbf{z}_m(k) = 1|\hat{\mathbf{\Sigma}})P(\Delta\mathbf{x}_m|\mathbf{z}_m(k) = 1, \hat{\mathbf{\Sigma}})}{\sum_{j=1}^{K} P(\mathbf{z}_m(j) = 1|\hat{\mathbf{\Sigma}})P(\Delta\mathbf{x}_m|z_m(j) = 1, \hat{\mathbf{\Sigma}})} \\
&= \frac{\pi_k P(\Delta\mathbf{x}_m|\mathbf{\Sigma}_k)}{\sum_{j=1}^{K}\pi_j P(\Delta\mathbf{x}_m|\mathbf{\Sigma}_j)},
\end{aligned}$$

To determine the maximized forms of D_k from $\mathbf{\Sigma}_k$, we equate the maximized covariance matrix (Eq. S5) with the theoretical covariance matrix (Eq. 3) in a manner similar to the covariance-based estimator [49]. It is useful to equate the diagonal elements and the first off-diagonal elements separately. Equating the diagonal elements of the theoretical covariance matrix, which is equal to $2D_k\Delta t + 2\sigma_k^2 - 4RD_k\Delta t$, to the mean diagonal elements of the maximized $\mathbf{\Sigma}_k$ yields:

$$\begin{aligned}
\mathrm{diag}\left[\mathbf{\Sigma}_k\right] &= \left\langle \mathrm{diag}\left[\frac{1}{M_k}\sum_{m=1}^{M}\gamma_{mk}\Delta\mathbf{x}_m\Delta\mathbf{x}_m^T\right]\right\rangle \\
2D_k\Delta t + 2\sigma_k^2 - 4RD_k\Delta t &= \left\langle \frac{1}{M_k}\sum_{m=1}^{M}\gamma_{mk}\ \mathrm{diag}\left[\Delta\mathbf{x}_m\Delta\mathbf{x}_m^T\right]\right\rangle \\
2D_k\Delta t + 2\sigma_k^2 - 4RD_k\Delta t &= \frac{1}{M_k}\sum_{m=1}^{M}\gamma_{mk}\ \left\langle \mathrm{diag}\left[\Delta\mathbf{x}_m\Delta\mathbf{x}_m^T\right]\right\rangle \\
2D_k\Delta t + 2\sigma_k^2 - 4RD_k\Delta t &= \frac{1}{M_k}\sum_{m=1}^{M}\gamma_{mk}\langle\Delta x_m(n)^2\rangle \qquad \text{(Eq. S6)}
\end{aligned}$$

where $\langle\Delta x_m(n)^2\rangle = \frac{1}{N_m}\sum_{n=1}^{N_m}\Delta x_m(n)^2$ represents the mean of the N_m displacements square for protein trajectory m, and $\Delta\mathbf{x}_m = \{\Delta x_m(n)\}_{n=1}^{N_m}$.

Similarly, equating the nearest-neighbor covariance of the theoretical covariance matrix, which is equal to $-\sigma_k^2 - 2RD_k\Delta t$, to the mean nearest-neighbor covariance of the maximized covariance matrix, $\mathrm{corr}\left[\mathbf{\Sigma}_k\right]_{n,n+1}$, yields:

$$\begin{aligned}
\mathrm{corr}\left[\mathbf{\Sigma}_k\right]_{n,n+1} &= \left\langle \mathrm{corr}\left[\frac{1}{M_k}\sum_{m=1}^{M}\gamma_{mk}\Delta\mathbf{x}_m\Delta\mathbf{x}_m^T\right]_{n,n+1}\right\rangle \\
-\sigma_k^2 + 2RD_k\Delta t &= \left\langle \frac{1}{M_k}\sum_{m=1}^{M}\gamma_{mk}\ \mathrm{corr}\left[\Delta\mathbf{x}_m\Delta\mathbf{x}_m^T\right]_{n,n+1}\right\rangle \\
-\sigma_k^2 + 2RD_k\Delta t &= \frac{1}{M_k}\sum_{m=1}^{M}\gamma_{mk}\ \left\langle \mathrm{corr}\left[\Delta\mathbf{x}_m\Delta\mathbf{x}_m^T\right]_{n,n+1}\right\rangle \\
-\sigma_k^2 + 2RD_k\Delta t &= \frac{1}{M_k}\sum_{m=1}^{M}\gamma_{mk}\langle\Delta x_m(n)\Delta x_m(n+1)\rangle \qquad \text{(Eq. S7)}
\end{aligned}$$

where $\langle\Delta x_m(n)\Delta x_m(n+1)\rangle = \frac{1}{N_m-1}\sum_{n=1}^{N_m-1}\Delta x_m(n)\Delta x_m(n+1)$ is the mean correlation between nearest neighbor displacements for protein trajectory m.

Using the relations for the diagonal terms (Eq. S6) and off-diagonal terms (Eq. S7), the maximized diffusivity estimates for diffusive state k can be solved according to:

$$
\begin{aligned}
2D_k\Delta t &= \operatorname{diag}[\mathbf{\Sigma}_k] + 2 \operatorname{corr}[\mathbf{\Sigma}_k]_{n,n+1} \\
2D_k\Delta t &= \frac{1}{M_k}\sum_{m=1}^{M}\gamma_{mk}\langle\Delta x_m(n)^2\rangle + \frac{2}{M_k}\sum_{m=1}^{M}\gamma_{mk}\langle\Delta x_m(n)\Delta x_m(n+1)\rangle \\
2D_k\Delta t &= \frac{1}{M_k}\sum_{m=1}^{M}\gamma_{mk}\left(\langle\Delta x_m(n)^2\rangle + 2\langle\Delta x_m(n)\Delta x_m(n+1)\rangle\right) \\
D_k &= \frac{1}{2\Delta t M_k}\sum_{m=1}^{M}\gamma_{mk}\left(\langle\Delta x_m(n)^2\rangle + 2\langle\Delta x_m(n)\Delta x_m(n+1)\rangle\right) \qquad \text{(Eq. S8)}
\end{aligned}
$$

Analogously, the maximized static localization noise estimates for diffusive state k can be solved by substituting Eq. S8 into Eq. S6, and solving for σ_k^2 according to:

$$
\begin{aligned}
2D_k\Delta t + 2\sigma_k^2 - 4RD_k\Delta t &= \frac{1}{M_k}\sum_{m=1}^{M}\gamma_{mk}\langle\Delta x_m(n)^2\rangle \\
\sigma_k^2 &= \frac{1}{2M_k}\sum_{m=1}^{M}\gamma_{mk}\langle\Delta x_m(n)^2\rangle - D_k\Delta t(1-2R),
\end{aligned}
$$

where the square root of σ_k^2 gives the static localization noise.

The covariance-based maximization equations for D_k and σ_k represent the posterior-weighted average of the covariance-based estimators for each diffusive state across the population of protein trajectories. It should be noted that the covariance-based estimator was shown to be nearly optimal and unbiased when analyzing protein trajectories on an track-by-track basis [49].

Derivation of π_k

To find π_k, we maximize the log-likelihood (Eq. 1) with respect to π_k subject to the constraint that $\sum_{k=1}^{K}\pi_k = 1$, which we implemented via a Lagrange multiplier, λ.

$$
\begin{aligned}
0 &= \frac{\partial}{\partial \pi_k}\left[\ln \mathcal{L}(\Delta\hat{\mathbf{x}}|\hat{\boldsymbol{\pi}},\hat{\boldsymbol{\Sigma}}) + \lambda\left(\sum_{k=1}^{K}\pi_k - 1\right)\right] \\
&= \sum_{m=1}^{M}\frac{\partial}{\partial \pi_k}\left[\ln\left\{\sum_{k=1}^{K}\pi_k P(\Delta\mathbf{x}_m|\boldsymbol{\Sigma}_k)\right\}\right] + \lambda\frac{\partial}{\partial \pi_k}\left[\left(\sum_{k=1}^{K}\pi_k - 1\right)\right] \\
&= \sum_{m=1}^{M}\frac{P(\Delta\mathbf{x}_m|\boldsymbol{\Sigma}_k)}{\sum_{j=1}^{K}\pi_j P(\Delta\mathbf{x}_m|\boldsymbol{\Sigma}_j)} + \lambda \\
\lambda &= -\sum_{m=1}^{M}\frac{P(\Delta\mathbf{x}_m|\boldsymbol{\Sigma}_k)}{\sum_{k=1}^{K}\pi_k P(\Delta\mathbf{x}_m|\boldsymbol{\Sigma}_k)} \\
\sum_{k=1}^{K}\pi_k\lambda &= -\sum_{k=1}^{K}\pi_k\sum_{m=1}^{M}\frac{P(\Delta\mathbf{x}_m|\boldsymbol{\Sigma}_k)}{\sum_{j=1}^{K}\pi_j P(\Delta\mathbf{x}_m|\boldsymbol{\Sigma}_j)} \\
\lambda &= -\sum_{m=1}^{M}\frac{\sum_{k=1}^{K}\pi_k P(\Delta\mathbf{x}_m|\boldsymbol{\Sigma}_k)}{\sum_{j=1}^{K}\pi_j P(\Delta\mathbf{x}_m|\boldsymbol{\Sigma}_j)} \\
\lambda &= -\sum_{m=1}^{M}1 \\
\lambda &= -M.
\end{aligned}
$$

Substituting λ back in to the maximized equation and solving for π_k, we find:

$$
\begin{aligned}
0 &= \sum_{m=1}^{M}\frac{P(\Delta\mathbf{x}_m|\boldsymbol{\Sigma}_k)}{\sum_{j=1}^{K}\pi_j P(\Delta\mathbf{x}_m|\boldsymbol{\Sigma}_j)} + \lambda \\
M &= \sum_{m=1}^{M}\frac{P(\Delta\mathbf{x}_m|\boldsymbol{\Sigma}_k)}{\sum_{j=1}^{K}\pi_j P(\Delta\mathbf{x}_m|\boldsymbol{\Sigma}_j)} \\
\pi_k M &= \sum_{m=1}^{M}\frac{\pi_k P(\Delta\mathbf{x}_m|\boldsymbol{\Sigma}_k)}{\sum_{j=1}^{K}\pi_j P(\Delta\mathbf{x}_m|\boldsymbol{\Sigma}_j)} \\
\pi_k M &= \sum_{m=1}^{M}\gamma_{mk} \\
\pi_k &= \frac{M_k}{M},
\end{aligned}
$$

where $M_k = \sum_{m=1}^{M}\gamma_{mk}$.
