# Supplementary material for: Extracting Diffusive States of Rho GTPase in Live Cells: Towards In Vivo Biochemistry
Source: PLoS Comput Biol. 2015 Oct 29;11(10):e1004297. doi: 10.1371/journal.pcbi.1004297 (PMC4626024; doi:10.1371/journal.pcbi.1004297)
Supplement: S3 Text — (PDF) [file pcbi.1004297.s019.pdf]

S3 Text

Performance comparison of pEM versus standard EM

Example of how pEM improves EM estimates

pEM improves upon standard EM procedures by incorporating a mechanism that permits escape from local maxima of the likelihood surface, which can occur as a result of a poor choice of initial values. In particular, when the number of protein trajectories is too low, the likelihood surface may be noisy, leading the EM to be potentially more sensitive to initialization. S15 Fig. shows the evolution of the parameter estimates with iteration number through our pEM procedure to completion for 500 synthetic protein trajectories simulated according to case 2. The parameter estimates make a discontinuous jump at the start of each perturbation trial, but evolve smoothly within each trial until convergence has been reached, as expected for EM learning. The initial EM procedure with a random initialization converges to a local maximum, corresponding to the parameter values achieved at the number of iterations marked by the black vertical dashes. After further perturbation trials, however, a path to escape the local maximum is generated, initiated by the perturbation imposed at the number of iterations highlighted by the red vertical dashed line. When pEM approaches the global maximum, each perturbation trial completes very rapidly. Recall that the parameter estimates are only updated if a higher maximum likelihood value is found within the original data set. Thus, although the EM may find new parameter estimates on the perturbed likelihood landscape, most perturbation trials do not change the stored parameter estimates, which still remain optimal for the original likelihood landscape.

S15 Fig. Example pEM estimation on synthetic protein trajectories. Diffusivity and population fraction estimates of 50 perturbation trials of pEM for 500 synthetic protein trajectories given according to case 2 plotted versus iteration number on a logarithmic scale. The black vertical dashes mark the iteration upon completion of the initial EM algorithm with a random initialization. The red vertical dashes mark the beginning of the iteration where pEM became successful at escaping the local maximum. The ground truth, *i.e.* the simulated values, is shown as horizontal dashed lines. Each color corresponds to a particular diffusive state.

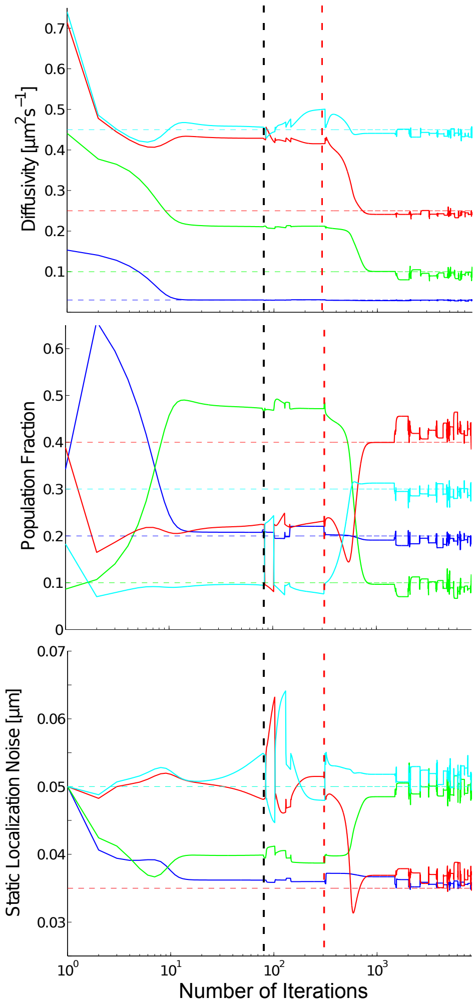

Comparison of pEM and EM estimates

The standard procedure to combat convergence to local maxima is to employ the EM algorithm with different initial parameters, a procedure called reinitialization expectation-maximization (rEM). The estimates which correspond to the rEM trial which yielded the highest maximum likelihood is then chosen as the final estimate. For rEM to be effective, a large number of reinitialization is required to sufficiently explore parameter space. The recommended number of trials, however, is not readily specified. Alternatively, we have introduced pEM, which performs one expectation-maximization trial and

subsequently applies a predetermined number of perturbation trials to sample the likelihood surface for an escape path. Similar to rEM, the number of perturbations is largely unknown. We shall see below, that the number of perturbations required depends on the complexity of the underlying data set.

To test the consistency of pEM's improved performance over rEM, we generated 3 sets of simulated data: 500 synthetic protein trajectories given by case 1 with a constant static localization noise, 1,000 synthetic protein trajectories given by case 2 with a constant static localization noise, and 5,000 synthetic protein trajectories given by case 3 with variable static localization noise (S1 Table). For each data set, the EM procedure was rerun starting from 10 different random initial parameters. Upon the completion of each EM run, we subsequently applied pEM with 100 perturbations initialized with the estimates found by each EM trial. Thus, each trial serves to show the variability of the EM and pEM estimates with random parameter initializations. S16 Fig. shows the maximum likelihood values found by each EM trial (shown in green), the maximum likelihood values determined by pEM (shown in red), and the theoretical maximum likelihood value calculated from the simulated parameter values (shown as a black dashed line). The mean and standard deviation of the parameter values given by EM and pEM are given in S4 Table, S5 Table, and S6 Table.

The best estimates given by rEM for case 1 are similar to the pEM estimates. However this results only occurs in 3 out 10 trials. In the other 7 cases, the estimates are very poor and, based on their BIC score, would favor a 3 state model. For case 2, the EM estimates corresponding to the highest maximum likelihood value occurred in 7 of the 10 trials. While these estimates are relatively close to the ground truth values, the subsequent application of pEM clearly improves upon the EM estimates (S5 Table), and correspondingly reaches the global maximum likelihood (S16 Fig.). For case 3, EM consistently yields suboptimal convergence, while pEM is able to reach the global maximum likelihood (S16 Fig.).

Evidently, pEM is able to consistently improve upon the standard EM algorithm by yielding estimates which converge to the global maximum likelihood, and in some trials, surpassing it. Although, in principle, the likelihood value calculated with the simulated parameters should correspond to the global maximum likelihood, the stochastic nature of diffusion and the statistical sampling of each diffusive state render the experimental global maximum likelihood slightly different from the theoretical global maximum likelihood.

Although error bars are presented in S4 Table, S5 Table, and S6 Table, in general, representative error estimation is problematic with multi-class problems. Traditional methods to provide errors of EM convergence relies on bootstrapping, which only characterizes the variability in the topology of a converged maximum likelihood. Moreover, these errors do not intrinsically give a sense of whether the model size is correct. Thus, the determination of proper errors is not straightforward for these kinds of problems.

S16 Fig. Representative comparison of the maximum likelihood found by pEM and EM with different initial parameters. Maximum likelihood values acquired by pEM (red) and EM (green) applied to (left) 500 synthetic protein trajectories corresponding to case 1 with a constant static localization noise, (middle) 1,000 synthetic protein trajectories corresponding to case 2 with a constant static localization noise, and (right) 5,000 synthetic protein trajectories corresponding to case 3 with variable static localization noises for the correct model size. Each of the EM data points represent the maximum likelihood value found from a single EM run with different initial parameter values. Each pEM data point represents the maximum likelihood value found by applying 100 perturbation trials starting from the EM converged parameters. The horizontal dashed line indicates the maximum likelihood value calculated using the simulation parameters.

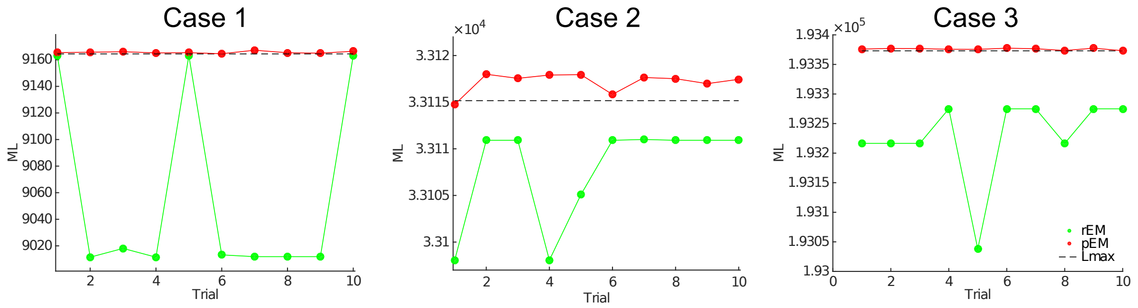

S4 Table. Comparison of EM and pEM on 500 synthetic protein trajectories given according to case 1. Average diffusivity, static localization noise, and population fraction estimates from 10 EM trials with different initializations and pEM, which consists of 100 perturbation trials initialized with each EM converged estimates from each trial, for each diffusive state given the correct model size. The errors represent the standard deviation of the estimates for each diffusive state across the 10 trials. Units: D [μm^{2}s^{-1}], σ [μm].

D^{sim}	0.01	0.30	1.20	2.8
D_{EM}	0.012 ± 0.001	0.22 ± 0.14	0.89 ± 0.42	2.37 ± 0.71
D_{pEM}	0.010 ± 0.001	0.31 ± 0.01	1.20 ± 0.02	2.97 ± 0.14
σ^{sim}	0.05	0.05	0.05	0.05
σ_{EM}	0.050 ± 0.002	0.042 ± 0.016	0.056 ± 0.007	0.056 ± 0.001
σ_{pEM}	0.050 ± 0.001	0.050 ± 0.001	0.056 ± 0.003	0.053 ± 0.005
π^{sim}	0.20	0.30	0.40	0.10
π_{EM}	0.188 ± 0.036	0.229 ± 0.135	0.343 ± 0.126	0.240 ± 0.201
π_{pEM}	0.201 ± 0.008	0.304 ± 0.012	0.408 ± 0.010	0.087 ± 0.004

S5 Table. Comparison of EM and pEM on 1,000 synthetic protein trajectories given according to case 2. Average diffusivity, static localization noise, and population fraction estimates from 10 EM trials with different initializations and pEM, which consists of 100 perturbation trials initialized with each EM converged estimates from each trial, for each diffusive state given the correct model size. The errors represent the standard deviation of the estimates for each diffusive state across the 10 trials. Units: D [$\mu m^2 s^{-1}$], σ [μm].

D^{sim}	0.03	0.10	0.25	0.45
D_{EM}	0.027 ± 0.004	0.075 ± 0.052	0.245 ± 0.071	0.511 ± 0.207
D_{pEM}	0.030 ± 0.001	0.099 ± 0.010	0.243 ± 0.007	0.451 ± 0.009
σ^{sim}	0.05	0.05	0.05	0.05
σ_{EM}	0.048 ± 0.006	0.052 ± 0.002	0.049 ± 0.001	0.049 ± 0.007
σ_{pEM}	0.051 ± 0.001	0.049 ± 0.002	0.050 ± 0.001	0.052 ± 0.001
π^{sim}	0.20	0.10	0.40	0.30
π_{EM}	0.140 ± 0.068	0.159 ± 0.090	0.375 ± 0.035	0.326 ± 0.142
π_{pEM}	0.202 ± 0.009	0.088 ± 0.011	0.402 ± 0.018	0.309 ± 0.023

S6 Table. Comparison of EM and pEM on 5,000 synthetic protein trajectories given according to case 3. Average diffusivity, static localization noise, and population fraction estimates from 10 EM trials with different initializations and pEM, which consists of 100 perturbation trials initialized with each EM converged estimates from each trial, for each diffusive state given the correct model size. The errors represent the standard deviation of the estimates for each diffusive state across the 10 trials. Units: D [$\mu m^2 s^{-1}$], σ [μm].

D^{sim}	1e − 8	0.01	0.06	0.16	0.34	0.55	0.94
D_{EM}	$0.0001 \pm$ 1e − 5	0.015 ± 0.003	0.065 ± 0.031	0.188 ± 0.067	0.334 ± 0.063	0.445 ± 0.088	0.787 ± 0.118
D_{pEM}	3e − 8 $\pm$ 1e − 6	0.012 ± 0.001	0.063 ± 0.002	0.172 ± 0.006	0.357 ± 0.010	0.537 ± 0.009	0.933 ± 0.030
σ^{sim}	0.03	0.035	0.04	0.05	0.04	0.05	0.06
σ_{EM}	0.0300 ± 0.0001	0.033 ± 0.003	0.041 ± 0.004	0.041 ± 0.015	0.034 ± 0.018	0.048 ± 0.004	0.055 ± 0.001
σ_{pEM}	0.0300 ± 0.0001	0.034 ± 0.001	0.041 ± 0.001	0.048 ± 0.001	0.040 ± 0.001	0.053 ± 0.001	0.055 ± 0.002
π^{sim}	0.2	0.02	0.05	0.1	0.28	0.3	0.05
π_{EM}	0.201 ± 0.068	0.002 ± 0.090	0.008 ± 0.035	0.032 ± 0.087	0.071 ± 0.134	0.511 ± 0.107	0.158 ± 0.102
π_{pEM}	0.200 ± 0.004	0.022 ± 0.002	0.048 ± 0.003	0.116 ± 0.011	0.272 ± 0.010	0.283 ± 0.012	0.059 ± 0.007

Comparison of pEM and EM efficiency

Despite generating new estimates on a perturbed landscape, by construction pEM never moves downwards on the original likelihood surface, because pEM does not update the parameter estimates if the new estimates from the perturbed likelihood landscape in fact yield a lower value on the original likelihood landscape. Thus, pEM only serves as a tool to improve the estimate of the standard EM algorithm. Consequently, the majority of pEM perturbations do not result in changes to the parameter estimates.

To compare the efficiency of pEM and EM with multiple initializations, we assess the total number of iterations for each procedure for the data set described in S16 Fig. For case 1, the total number of iterations for the EM with 10 different initialization trials was 294 ± 190, while the average number of iterations for pEM, which included 1 EM trial and 100 perturbation trials, was 149 ± 185. For case 2, the total number of iterations for the EM with 10 different initialization trials was $10,200 \pm 7090$, while the average number of iterations for pEM was 5500 ± 1192. For case 3, the total number of iterations for the EM with 10 different initialization trials was 6830 ± 5130 , while the average number of iterations for pEM was 2083 ± 519. Evidently, not only does pEM yield better estimates compared to rEM, but it also does so more efficiently.

The 10 reinitialization trials wasn't sufficient to explore the region of parameter space where the EM can converge to the global maximum. Increasing the number of reinitialization trials, however, increases the total computational time. pEM, on the other hand, only employs the EM once and then applies subsequent perturbation trials. The average number of perturbations necessary to reach the global maximum for case 1, case 2, and case 3 was 42 ± 15, 16 ± 10, and 73 ± 20, respectively.

The number of perturbations necessary depends on the complexity of the underlying data set. There is no preset number of perturbations which guarantees that pEM will escape from a local maximum, since perturbations are inherently stochastic. Thus, the process of pEM can be inefficient at times, especially if the global maximum has already been reached. To speed up pEM convergence, it could also be beneficial to employ EM with multiple initializations in tandem with pEM, where the best estimates from EM with multiple initializations is used as the initial parameter for pEM.
