# Supplementary material for: Extracting Diffusive States of Rho GTPase in Live Cells: Towards In Vivo Biochemistry
Source: PLoS Comput Biol. 2015 Oct 29;11(10):e1004297. doi: 10.1371/journal.pcbi.1004297 (PMC4626024; doi:10.1371/journal.pcbi.1004297)
Supplement: S4 Text — (PDF) [file pcbi.1004297.s020.pdf]

S4 Text

Classifying protein trajectories to respective diffusive states

To provide a visual representation of pEM's ability to classify protein trajectories into different diffusive states, we spatially distribute the synthetic protein trajectories onto a rectangular "cell image", where each pixel represents a protein trajectory and the color of each pixel corresponds to a particular diffusive state. Examples of such images are shown in S17 Fig. When the underlying diffusive states are well-separated, as in case 1, classification of each protein trajectory into a particular diffusive state can be made with high fidelity on the basis of the maximum posterior probability (classified images in S17 Fig.). However, when the component distributions of two underlying diffusive states overlap significantly, as in case 2 and case 3 (see Fig. 2 in the Main Text), the accuracy of classification on the basis of the maximum posterior probability becomes increasingly unreliable. Furthermore, it is a major drawback with all-or-nothing classification into a definite state, that, after such a classification, every protein trajectory becomes equally weighted. Therefore, all information concerning the confidence that a given protein trajectory belongs to a specific diffusive state, which is given by the posterior probability, is lost.

S17 Fig. Representative posterior-weighted classification scheme for synthetic protein trajectories. Representative diffusive state classification from 10,000 synthetic protein trajectories for case 1 (top row) and case 2 (middle row), and 15,000 synthetic protein trajectories for case 3 (bottom row). In the first column, each pixel of the images corresponds to a single protein trajectory and is rendered with a color corresponding to the particular diffusive state used in the simulations. Cases 1 and 2 are represented by 100 pixels by 100 pixels, while case 3 is represented by 150 pixels by 100 pixels. The second column uses the same protein trajectories and color scheme, but the trajectories have now been classified according to their maximum posterior probability. In columns 3 through 9, each image again depicts the same protein trajectories, but now is rendered using a color scale that represents the posterior probability that the trajectory corresponds to the given diffusive state. White represents a low posterior probability and dark red represents a high posterior probability.

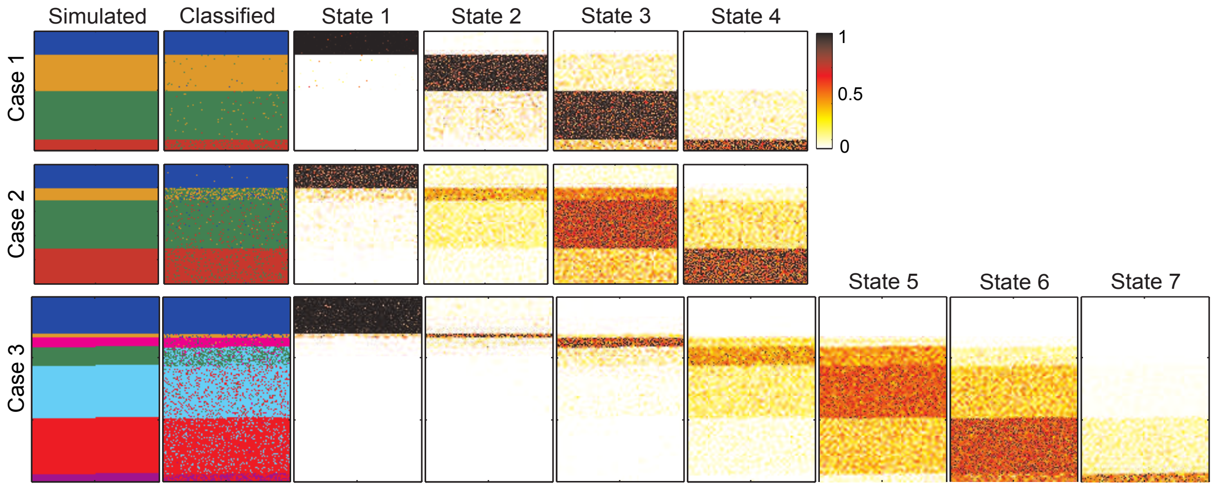

Alternatively, the spatial distribution of the protein trajectories for each diffusive state may be better represented by displaying the protein trajectories with a color corresponding to the magnitude of the posterior probability as a heat map (State images in S17 Fig.). Here, each panel corresponds to a different diffusive state, and within each panel, each point corresponds to a single trajectory and is rendered using a color scale that represents the posterior probability that the trajectory corresponds to the diffusive state in question. Zero and very low posterior probabilities are rendered in white, which is the same

as the background. Yellow, orange, red, and dark red correspond to progressively increasing posterior probabilities. In this manner, the relative likelihood that a protein trajectory was generated from a given diffusive state can be conveniently and intuitively visualized. Strikingly, the regions of high posterior probabilities are segmented well for case 1 (top row of state images in S17 Fig.). The fact that the neighboring diffusive states display a significantly lower posterior probability suggests that classification can be accomplished with high fidelity, as was observed with maximum posterior classification. On the other hand, case 2 shows that three diffusive states can be classified with high confidence (middle row of state images in S17 Fig.), namely states 1, 3 and 4. Diffusive state 2, on the other hand, does not exhibit a high posterior probability as a direct consequence of its relatively lower population fraction. Hence, the neighboring diffusive state, which has a higher population fraction, yields significant posterior probabilities in this region. Nevertheless, it is apparent that diffusive state 2, in isolation, yields relatively significant posterior probabilities within the appropriate region. For case 3, state 1, state 2, state 3, and state 7 exhibit high-contrast bands, indicating that the protein trajectories for these diffusive states can be classified with high fidelity (bottom row of State images in S17 Fig.). On the other hand, the bands for state 4, state 5, and state 6 display less contrast with their neighbors, corresponding to significant posterior probabilities of incorrect classification, when different states have similar parameters.

It should be noted that additional properties for each diffusive state, beyond D_k, σ_k^2, and π_k (which all emerge directly from pEM), may also be of interest, including, for example, the mean duration of protein trajectories for each diffusive state, or the symmetry of protein trajectories for each diffusive state, or the radius of gyration of protein trajectories for each diffusive state, *etc.*. These properties can properly be determined by calculating the posterior-weighted average of the desired property. Specifically, the property $\bar{\phi}_k$ for state k, given a population of M protein trajectories, is calculated according to

$$\bar{\phi}_k = \frac{1}{M_k} \sum_{m=1}^{M} \gamma_{mk} \phi_m$$

where ϕ_m is the protein trajectory property for trajectory m, and $M_k = \sum_{m=1}^{M} \gamma_{mk}$. Evidently, every protein trajectory is included and contributes to a particular diffusive state property, weighted by the posterior probability of being in that diffusive state.
