# Supplementary material for: Extracting Diffusive States of Rho GTPase in Live Cells: Towards In Vivo Biochemistry
Source: PLoS Comput Biol. 2015 Oct 29;11(10):e1004297. doi: 10.1371/journal.pcbi.1004297 (PMC4626024; doi:10.1371/journal.pcbi.1004297)

S5 Text

The dependence of pEM's performance on track length

For the majority of our simulations, each protein track length was a random variable drawn from an exponential distribution representative of our experimental protein track lengths. In our particle tracking algorithm, we set the minimum cutoff for a protein trajectory to be 15 steps. In this section, we motivate this choice. To this end, we explore pEM's performance on various synthetic protein trajectories given by case 2, but here, we constrain each protein trajectory to have the same length. Thus, we can better understand the limits of pEM as it relates to the length of the protein trajectories. Since longer protein trajectories contain more statistics, a fair comparison requires that each set of protein trajectories contain similar amounts of information. Thus, we compensate for the reduced information of short trajectories by including more protein trajectories. Here, every set of synthetic protein trajectories contained 25,000 total positions, regardless of the protein track length. To exemplify, we analyzed 5,000 synthetic protein trajectories each with only 5 steps or 500 synthetic protein trajectories each with 50 steps.

S18 Fig. shows the average diffusivity, static localization noise, and population fraction estimates from pEM applied to 5 sets of simulated protein trajectories with different protein track lengths, from a minimum of 5 steps to a maximum of 50 steps. When protein trajectories have only 15 steps or fewer, pEM consistently fails to uncover all of the diffusive states, ultimately favoring a 3-diffusive-state model. However, when protein trajectories with 20 steps or more were analyzed, pEM consistently uncovered a 4-diffusive-state model with reliable parameter values.

By including all trajectories with a length of 15 steps and more, we ensured that the average protein track length was 25 steps, safely beyond 20 steps, where pEM starts to give reliable results. Of course this conclusion was verified by demonstrating that pEM indeed yields accurate estimates in Fig. 2, S5 Fig., S6 Fig., and S7 Fig.

We described in S1 Text, that ergodicity breaking from finite protein trajectories results in a broadening of the diffusivity distributions, with increasing skew to the left and an increasing positive tail for protein trajectories with shorter lengths. S19 Fig. shows the consequence of this ergodicity breaking for protein trajectories of various lengths. When protein trajectories only have 5 steps, then the distributions of each diffusive state overlaps significantly. So much so that the total distribution appears more-or-less continuous. As the protein trajectories becomes longer, however, the distributions become narrower, and more easily separable. Thus, the complexity of the underlying diffusive states is reduced and pEM is able to discriminate these diffusive states more consistently, despite analyzing fewer protein trajectories.

S18 Fig. pEM estimates for synthetic protein trajectories with a constant track length given by case 2. The average pEM estimates for each diffusive state: diffusivity (top), population fraction (middle), and static localization noise (bottom), determined by analyzing protein trajectories containing different number of steps given according to case 2. Each color corresponds to a different diffusive state. Each data point represents the average estimate from five sets of protein trajectories and the error bars represent the observed standard deviation. The horizontal dashes represent the ground truth, *i.e.* the values input to the simulation, with the color corresponding to the respective diffusive state in question. pEM yields a 3 diffusive state model for protein trajectories with 15 steps or less; while, pEM consistently yields a 4 diffusive state model for protein trajectories with 20 steps or more.

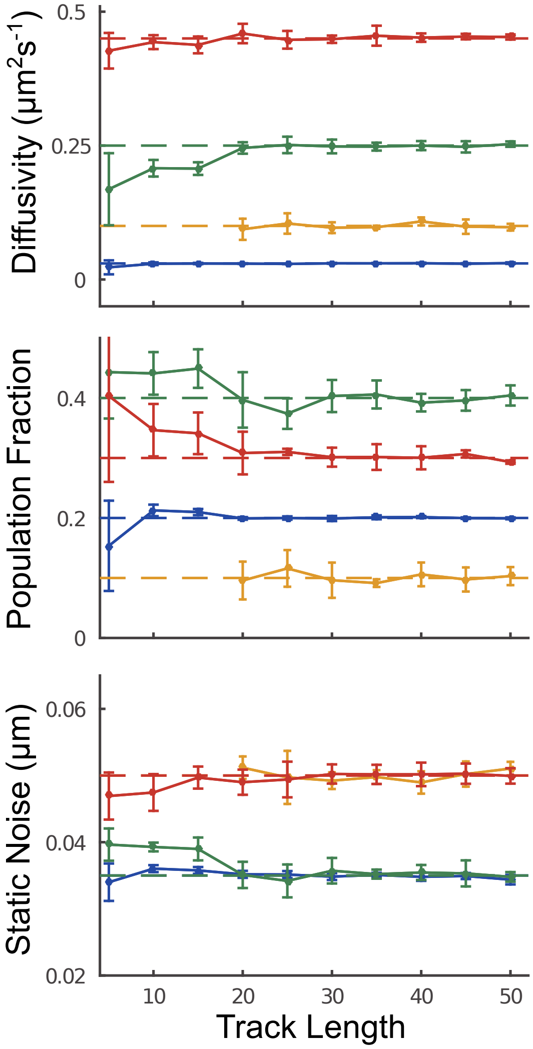

S19 Fig. Distribution of each diffusive state for synthetic protein trajectories given by case 2 for various transition probabilities. The probability distributions of each diffusive state (shown in a different color) generated by the covariance-based estimator analysis on synthetic protein trajectories for case 2 with a constant length: 5 steps (left), 25 steps (middle), and 50 steps (right). The inset of each figure shows the total diffusivity distribution using the same scale as the main figure.

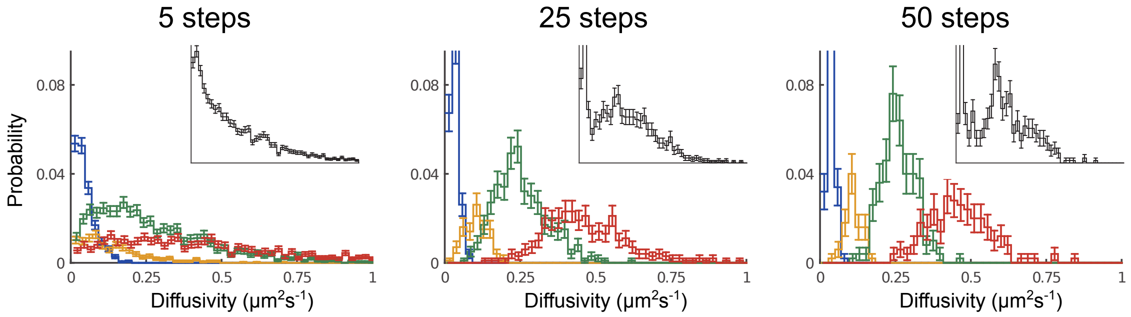

Supplement: S5 Text — (PDF) [file pcbi.1004297.s021.pdf]
