# Supplementary material for: Extracting Diffusive States of Rho GTPase in Live Cells: Towards In Vivo Biochemistry
Source: PLoS Comput Biol. 2015 Oct 29;11(10):e1004297. doi: 10.1371/journal.pcbi.1004297 (PMC4626024; doi:10.1371/journal.pcbi.1004297)

S6 Text

Testing pEM performance when assumptions are not satisfied

pEM makes two key assumptions: (1) the underlying diffusion coefficient and static localization noise are both constant throughout the duration of the protein trajectory, and (2) the underlying diffusion mode is normal. When these assumptions hold, we have shown, in the Main Text, that pEM provides accurate estimates for the number of diffusive states and the properties of each diffusive state, namely their diffusion coefficient, static localization noise, and population fraction. However, these assumptions may not necessarily hold in experimental data. In this section, we test the performance of pEM when these assumptions fail for protein trajectories which can transition between different diffusive states within a given trajectory, and for protein trajectories which contain a non-normal mode of diffusion.

pEM performance on synthetic protein trajectories that can transition between diffusive states

To test the performance of pEM against protein trajectories which transition between different diffusive states, we generated 5 sets of synthetic protein trajectories which transition between 4 diffusive states, namely $D_k = \{0.0001,\ 0.01,\ 0.07,\ 0.17\}$ μm^{2}s^{-1}, $\pi_k = \{0.3,\ 0.1,\ 0.2,\ 0.4\}$, and a constant static localization noise, $\sigma_k = 0.05$ μm for all of the states. To incorporate transitions into each diffusive state, we generated a random Markov chain, which represents the underlying state sequence, with a known transition matrix, A, which was constructed to have a higher probability to stay within the same state between 0.98 and 1 ($0.98 \leq A_{i,i} \leq 1$) , and equal probabilities to transition into different states $A_{i,j} = \frac{1-A_{i,i}}{K-1}$ for $i \neq j$. The values of the population fractions determine how many protein trajectories are initialized with a given diffusive state. To mimic the kinds of trajectories realized experimentally, the length of the trajectories is randomly sampled from an exponential distribution similar to Fig. 3c. Each protein trajectory also contain motion blur, as described in the Methods section.

Within this framework, we then generated 5 sets of 5,000 synthetic protein trajectories $A_{1,1} = \{1,\ 0.995,\ 0.99,\ 0.985,\ 0.98\}$. S20 Fig.a shows the fraction of trajectories with the given number of transitions as a function of $A_{1,1}$. Different numbers of total transitions per trajectory are shown in different colors. When $A_{1,1} = 1$, all of the protein trajectories contain zero transitions. When $A_{1,1} = 0.995$, 70% of the trajectories contain zero transition, 25% contain 1 transition, and 5% contain 2 transitions. S20 Fig.b shows the mean number of transitions per trajectory for various transition probabilities.

The diffusivity, population fraction, and static localization noise estimates from applying pEM to synthetic protein trajectories for various transition rates is shown in S21 Fig. As expected, when $A_{1,1} = 1$, pEM yields excellent estimates for the diffusivities, static localization noises, and population fractions. As the number of transitions per track increases, the diffusivity estimates remain reasonable. The population fraction estimates, however, are more sensitive to the underlying transition rates. As the transition rate increases, the fastest and slowest diffusive state populations decrease, while the intermediary diffusive state populations increase. The static localization noise estimates are insensitive to the transition probabilities as all of the protein trajectories have the same level of localization noise.

When vbSPT, which models for the ability to transition between different diffusive states, is applied to the same protein trajectories, vbSPT consistently yields a two diffusive state model (S21 Fig.). Consistent with the results of case 1 (S5 Fig.), case 2 (S6 Fig.), and case 3 (S7 Fig.), we attribute vbSPT's underperformance to not taking into account nearest-neighbor correlations, which deems noisy protein trajectories non-Markovian.

Since the transition rates between diffusive states is not known *a priori*, a visual test to assess the existence of transitions is desirable. S22 Fig. shows a plot of the population fractions as a function of the protein track duration for three values of the mean number of transitions per track. This is accomplished by selecting all protein trajectories which have a given length and then calculating the population fractions for each diffusive state within this subset of the data, according to Eq. 7. Since the

S20 Fig. Fraction of tracks versus the probability of no transitions per step for various numbers of transitions. (a) The fraction of tracks found from 5,000 synthetic protein trajectories which transition between 4 diffusive states with $D_k = \{0.0001,\ 0.01,\ 0.07,\ 0.17\}$ μm^{2}s^{-1}, $\pi_k = \{0.3,\ 0.1,\ 0.2,\ 0.4\}$, and a constant static localization noise, $\sigma_k = 0.05$ μm for all of the states versus $A_{1,1}$, where $A_{1,1}$ is the transition probabilities per step to stay within the same state and $A_{i,j} = \frac{1-A_{i,i}}{K-1}$ with $i \neq j$ is the transition probability per step to transition from state i to state j. Each curve, shown in a different color, represents the number of protein trajectories found with either 0, 1, 2, 3, 4, or 5 total transitions within the protein trajectory, divided by the total number of trajectories. (b) The average number of transitions per track versus $A_{1,1}$. The error bars represent the mean of the standard deviation of the number of transitions per track within each trial.

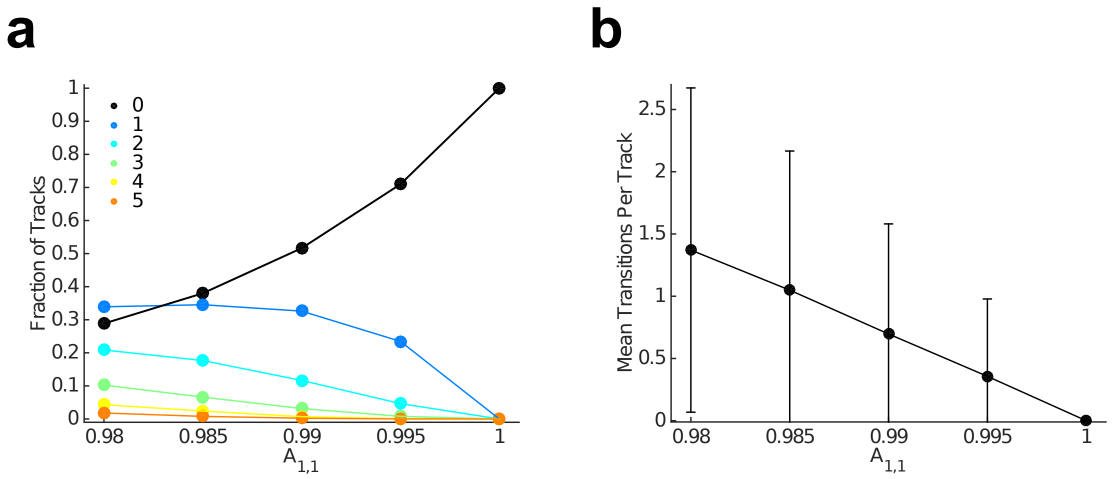

number of protein trajectories decreases exponentially with the track duration, there is less data for longer protein trajectories, and thus, these data points are more noisy.

When the protein trajectories contains no transitions, the population fractions reproduce the simulated values well, irrespective of the track duration. When the mean number of transitions per track is equal to 0.3, the population fractions appear to deviate from their short-track length values when the track durations become long. The changing behavior of the population fractions is more pronounced even for shorter protein trajectories when the mean number of transitions per track is equal to 1. Here, each protein trajectory contains, on average, 1 transition. These results demonstrate that the population fraction as a function of track duration can be used to assess the levels of transitions occurring within a collection of heterogeneous protein tracks.

S21 Fig. Comparison of pEM and vbSPT for synthetic protein trajectories that transition between 4 diffusive states. Diffusivity and population fraction estimates for 5,000 protein trajectories for versus the mean number of transitions per track between 4 diffusive states with $D_k = \{0.0001,\ 0.01,\ 0.07,\ 0.17\}$ $\mu\text{m}^2\text{s}^{-1}$, $\pi_k = \{0.3,\ 0.1,\ 0.2,\ 0.4\}$, and a constant static localization noise, $\sigma_k = 0.05$ μm for pEM (left) and vbSPT (right). The static localization noise is assumed to be known for vbSPT but must be determined by pEM (bottom-left). Each color corresponds to a different diffusive state. Each data point represents the average of five sets of synthetic protein trajectories and the error bars represent the observed standard deviations. The solid lines are guides-to-the-eye. The horizontal dashed lines represent the ground truth, *i.e.* the values input to the simulation. The color of each dashed line indicates the corresponding diffusive state.

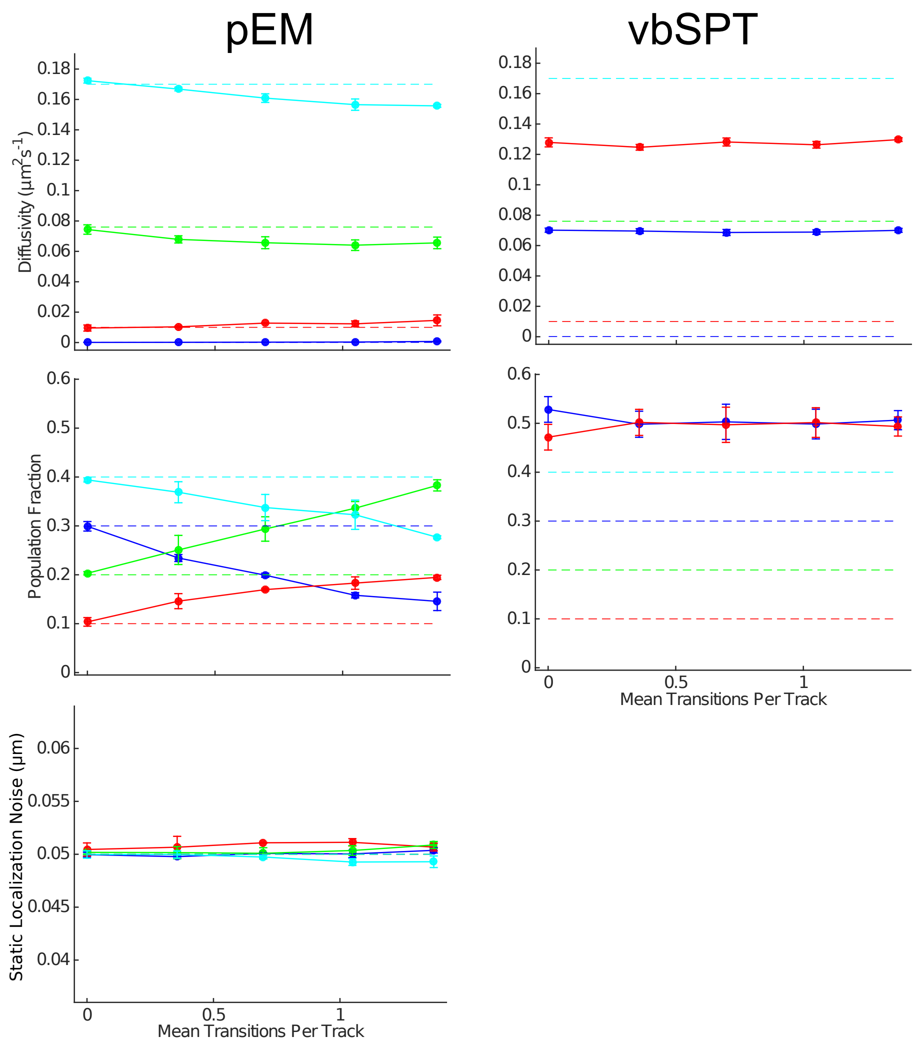

S22 Fig. Population fraction versus track duration for synthetic protein trajectories that transition between 4 diffusive states. The average population fraction calculated at each protein trajectory duration for a mean number of transition per trajectory of (a) 0, (b) 0.3, and (c) 1. Each diffusive state is shown in a different color: state 1 (dark blue), state 2 (red), state 3 (green), and state 4 (cyan). The minimum length of a protein trajectory was set by the tracking algorithm to 15 steps (0.48 s). The error bars represent the standard error of the mean. The horizontal dashed lines represent the simulated value for the population fractions, *i.e.* the values input to the simulation. The color of each dashed line indicates the corresponding diffusive state.

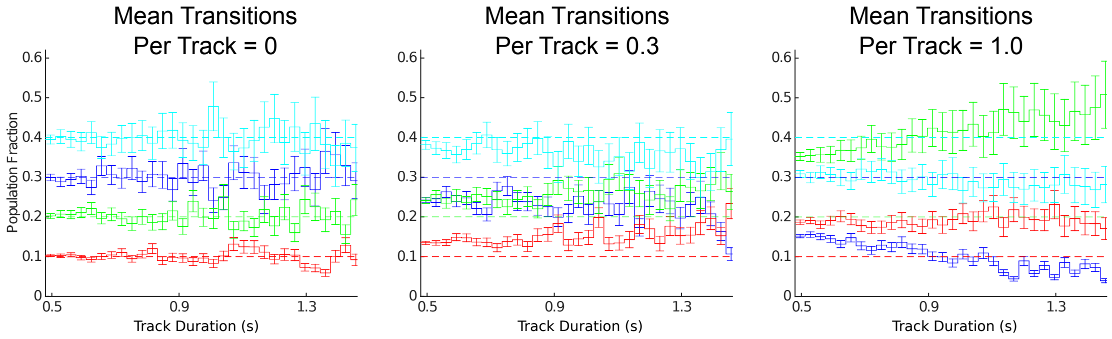

pEM performance on synthetic protein trajectories that have non-normal diffusive states

Besides assuming no transitions between diffusive states, pEM also makes the assumption that the underlying diffusion process is normal. To test the performance of pEM when this assumption is not met, we generated 3,000 synthetic protein trajectories with 2 diffusive states: (1) normal diffusion with $D = 0.2$ μm^{2}s^{-1} and (2) confined diffusion with $D = 0.1$ μm^{2}s^{-1} confined in a square geometry of size $L = [-0.15, 0.15]$ μm.

To generate synthetic protein trajectories undergoing normal diffusion confined to a finite square geometry with size $-L$ to L with reflecting boundary conditions, we simulate displacements following normal diffusion. At each time step, however, if the new position falls out of the finite domain, then the simulated position is set such that the difference between the proposed position and the boundary is reflected. In essence, the protein travels to the wall and gets reflected back, but the total travel distance remains the same as if the wall were not present. To allow for heterogeneous confinement sizes, we vary each confinement with a Gaussian random variable with zero mean and standard deviation of 0.1 μm.

To include transitions, we first generated a random Markov chain, which represents the underlying state sequence, with a known transition matrix. The population fraction was set to be equal at 0.5 for both state 1 and state 2. When the Markov state begins at state 2, the initial protein begins diffusing from the center of the confined geometry. When there is a switch to state 1, then the boundary constraint is released and the protein undergoes free diffusion with parameters of state 1. When the Markov state sequence changes from free diffusion to confined diffusion, we set the current position as the center of the new confinement. To mimic the kinds of trajectories we have experimentally, we impose that the length of the protein trajectories be randomly sampled from an exponentially decaying distribution similar distribution to Fig. 3c and that each protein trajectory also contain both motion blur, as described in the Methods section.

S23 Fig.a shows an example of the longer trajectories which contain 1 transition and 2 transitions, where the confined diffusion state is shown in green and the normal diffusion state is shown in blue. We applied pEM to the set of protein trajectories with $A_{1,1} = 0.997$, 0.99, and 0.95 per step, the corresponding mean number of transitions per trajectory was found to be 0.07, 0.24, and 1.2, respectively. For $A_{1,1} = 0.997$, the pEM estimates were $D_k = \{0.048,\ 0.194\}$ μm^{2}s^{-1}, $\sigma_k = \{0.053,\ 0.050\}$ μm, and $\pi_k = \{0.523,\ 0.477\}$. For $A_{1,1} = 0.99$, the pEM estimates were $D_k = \{0.0461,\ 0.1877\}$ μm^{2}s^{-1}, $\sigma_k = \{0.053,\ 0.0511\}$ μm, and $\pi_k = \{0.475,\ 0.525\}$. For $A_{1,1} = 0.95$, the pEM estimates were $D_k = \{0.058,\ 0.174\}$ μm^{2}s^{-1}, $\sigma_k = \{0.053,\ 0.051\}$ μm, and $\pi_k = \{0.434,\ 0.566\}$.

The posterior-weighted taMSD for each diffusive state (see S5 Text), however, clearly shows excellent agreement with the true taMSD calculated for each diffusive state, which can be accomplished because we know the underlying Markov sequence. As the transition rate increases to 1.2 transitions per track, however, the posterior-weighted taMSD does not correspond as well to the simulated taMSD. Notwithstanding, if a non-normal diffusion state were present within the population of protein trajectories, then a non-linear trend would manifest in the posterior-weighted taMSD, even though the diffusive states can transition. Thus, the posterior-weighted taMSD is a good method to discriminate whether any of the diffusive states follow non-normal diffusion.

S23 Fig. Performance of pEM on synthetic protein trajectories that transition between a confined diffusion and normal diffusion. (a) Example synthetic protein trajectory which has a single transition (left) and two transitions (right). The confined diffusion state is represented in green and the normal diffusion state is represented in blue. The scale bar represents 2 μm. (b) Posterior-weighted time-averaged MSD for each diffusive state (shown in a different color) for synthetic protein trajectories which transition between 2 diffusive states, namely a confined diffusion state (green) and a normal diffusion state (blue), for a number of transitions per trajectory equal to 0.07 ($A_{1,1}$ = 0.997), 0.24 ($A_{1,1}$ = 0.99), and 1.2 ($A_{1,1}$ = 0.95). The dashed curves represent the average taMSD curves calculated from the simulated state of each protein trajectory. The black dashed curve represents the average taMSD curve across all protein trajectories.

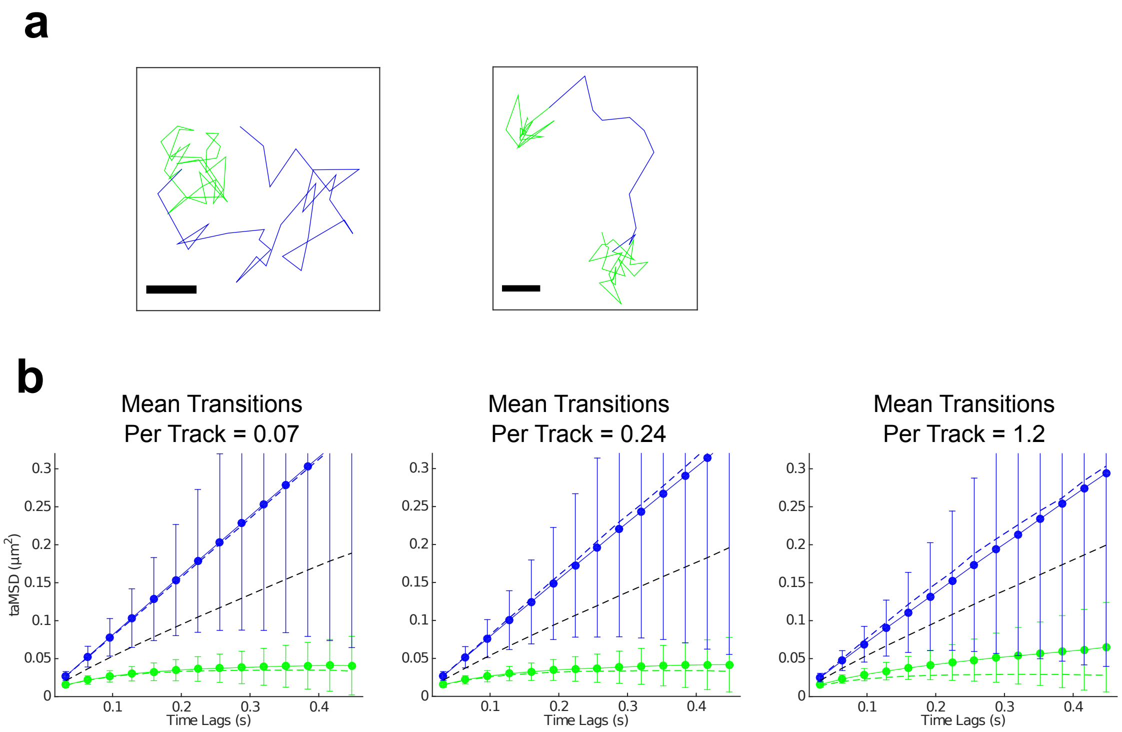

Supplement: S6 Text — (PDF) [file pcbi.1004297.s022.pdf]
