# Supplementary material for: Extracting Diffusive States of Rho GTPase in Live Cells: Towards In Vivo Biochemistry
Source: PLoS Comput Biol. 2015 Oct 29;11(10):e1004297. doi: 10.1371/journal.pcbi.1004297 (PMC4626024; doi:10.1371/journal.pcbi.1004297)

S7 Text

Validating pEM assumptions for protein trajectories of Rho GTPase

pEM makes two key assumptions: (1) the underlying diffusion mode is normal, and (2) the underlying diffusion coefficient and static localization noise are both constant throughout the duration of the protein trajectory. To test the validity of the first assumption, we have plotted the ensemble averaged MSD and the ensemble of the time-averaged MSDs for each of the mutants in S24 Fig. Evidently, the eaMSD and taMSD are in excellent agreement, indicating ergodic behavior, consistent with normal diffusion. In addition, each mutant exhibits a linear variation with time lag. Moreover, the posterior-weighted taMSDs is linear versus time for each diffusive state and each mutant (S25 Fig.). Thus, the assumption of normal diffusion is valid at the relevant timescales.

The second assumption pEM makes is that a given protein remains in the same diffusive state throughout the duration of its trajectory. To test this assumption, we grouped protein trajectories which have the same length, t, and calculated the posterior-weighted probability of being in each diffusive state, $\pi_k^{(t)}$, according to $\pi_k^{(t)} = \sum_{m=1}^{M_t} \gamma_{mk}^{(t)}/M_t$, where M_t is the total number protein trajectories with duration t and $\gamma_{mk}^{(t)}$ is the posterior probability of being in state k for protein trajectories of duration t. The corresponding population fractions versus protein track duration are shown in S26 Fig. S26 Fig. shows that the population fractions of each diffusive state remains constant for the first second in all variants.

Upon comparison to the trends observed for synthetic protein trajectories which transition between diffusive states (S22 Fig.), the apparent level of transitions is estimated to be less than 1 transition per track, a regime for which pEM is able to yield reasonable estimates.

S24 Fig. Ergodicity of Rho GTPase. (a-k) time-averaged MSD (black circles) averaged over the population of protein trajectories and the ensemble-averaged MSD (red squares) for the first 13 time lags of (a) RhoA, (b) RhoA G14V, (c) RhoA F30L, (d) RhoA T19N, (e) RhoA HV, (f) RhoC, (g) RhoC G14V, (h) RhoC F30L, (i) RhoC T19N, (j) RhoC HV, and (k) RhoC Chimera HV. The shaded region represents the standard deviation of the population of taMSD scatter at each time lag.

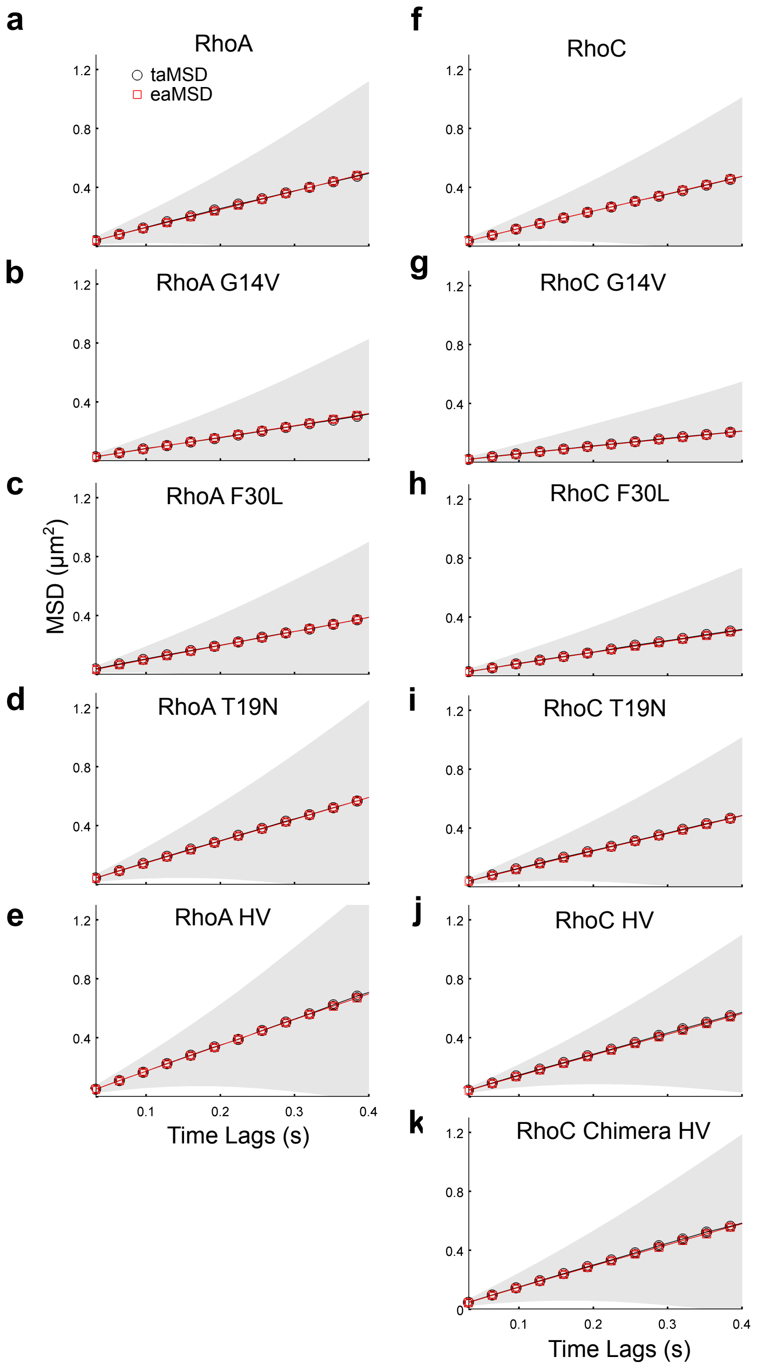

S25 Fig. Posterior-weighted time-averaged MSD for each diffusive state of Rho GTPase. (a-k) posterior-weighted taMSD for different diffusive states (shown in a different color) for the first 13 time lags of (a) RhoA, (b) RhoA G14V, (c) RhoA F30L, (d) RhoA T19N, (e) RhoA HV, (f) RhoC, (g) RhoC G14V, (h) RhoC F30L, (i) RhoC T19N, (j) RhoC HV, and (k) RhoC Chimera HV. The shaded region represents the standard deviation of the population of taMSD scatter about the mean taMSD at each time lag.

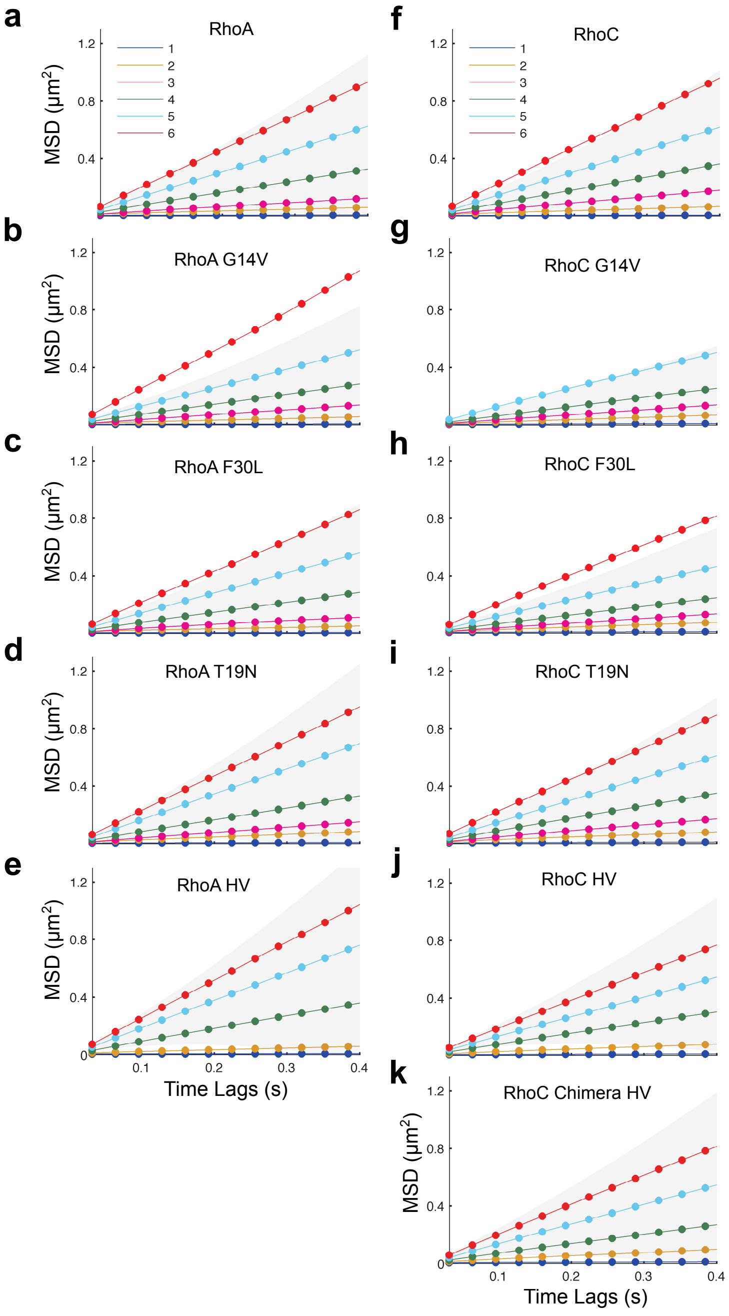

S26 Fig. Population fraction versus protein trajectory duration for each diffusive state of Rho GTPase. (a-k) The average population fraction calculated at each protein trajectory duration for each diffusive state: state 1 (blue), state 2 (orange), state 3 (magenta), state 4 (green), state 5 (cyan), and state 6 (red), for (a) RhoA, (b) RhoA G14V, (c) RhoA F30L, (d) RhoA T19N, (e) RhoA HV, (f) RhoC, (g) RhoC G14V, (h) RhoC F30L, (i) RhoC T19N, (j) RhoC HV, and (k) RhoC Chimera HV. The minimum length of a protein trajectory was set by the tracking algorithm to 15 steps (0.48 s). The error bars represent the standard error of the mean.

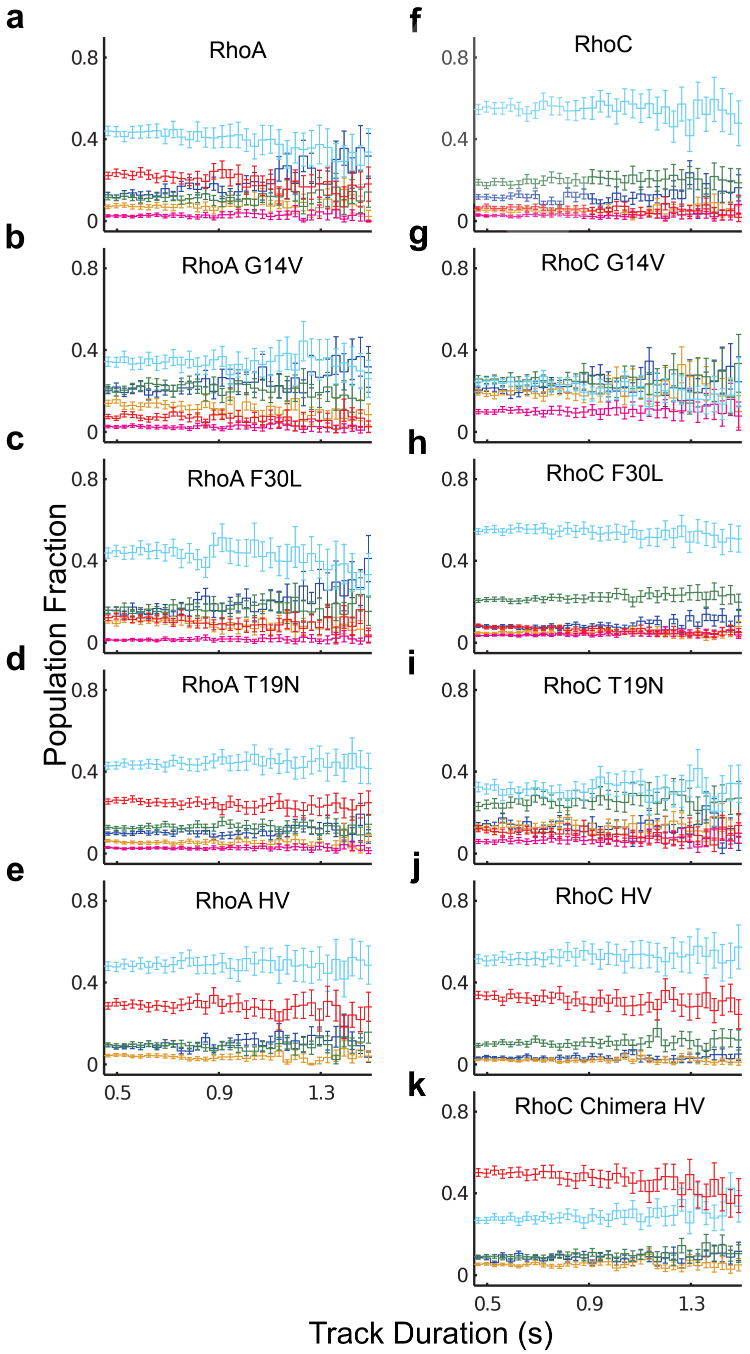

Supplement: S7 Text — (PDF) [file pcbi.1004297.s023.pdf]
