# Supplementary material for: Extracting Diffusive States of Rho GTPase in Live Cells: Towards In Vivo Biochemistry
Source: PLoS Comput Biol. 2015 Oct 29;11(10):e1004297. doi: 10.1371/journal.pcbi.1004297 (PMC4626024; doi:10.1371/journal.pcbi.1004297)

S8 Text

Determining the activation levels of Rho GTPase

To obtain an estimate of wild type Rho's activation state, we applied a minimization of the sum of the square difference between the cumulative distribution (CD) of the diffusivity for wild type (CD_{Rho}) from CVE analysis on individual protein trajectories with the weighted sum of the cumulative diffusivity distributions of the extreme activation states, CD_{G14V} and CD_{T19N}, where the weights of each distribution, w_i, are the free parameters, according to:

$$\arg\min_{w} \left\{ \sum_{D} \left[CD_{Rho} - (w_1 CD_{G14V} + w_2 CD_{T19N}) \right]^2 \right\}.$$

The CD are binned with a size of 0.001 μm^{2}s^{-1} to allow one-to-one correspondence across mutants.

Using this minimization scheme, the CD of RhoA was found to be 32% similar to the CD of RhoA G14V and 68% similar to the CD of RhoA T19N (S27 Fig., top-left); while, the CD of RhoC exhibited a stronger imbalance with 5% similar to the CD of RhoC G14V and 95% similar to the CD of RhoC T19N (S27 Fig., top-right). The fitted mixture of RhoA yielded low residuals for a wide range of diffusivities (S27 Fig., bottom-left), while RhoC yielded a slight divergence at lower diffusivities (S27 Fig., bottom-right).

Since global activation of Rho GTPases may potentially cause interference across other signaling pathways [66], the extent to which constitutively active and dominant negative mutations alters the underlying biology is unknown. The inability to exactly reproduce the wild type distribution is likely due to interruptions of normal signaling pathways when employing global activation of Rho via G14V and T19N mutants.

S27 Fig. Decomposition of the cumulative distribution of diffusivities for wild type Rho GTPase. (top-left) The MLE-generated cumulative distribution of diffusivities for wild type RhoA (blue), RhoA G14V (cyan), RhoA T19N (green), and a fitted mixture of RhoA G14V and RhoA T19N (red). (bottom-left) The residuals from subtracting the cumulative distribution of RhoA with RhoA G14V (cyan), RhoA T19N (green), and the fitted mixture of RhoA (red). (top-right) The cumulative distribution of diffusivities for wild type RhoC (blue), RhoC G14V (cyan), RhoC T19N (green), and a fitted mixture of RhoC G14V and RhoC T19N (red). (bottom-right) The residuals from subtracting the cumulative distribution of RhoC with RhoC G14V (cyan), RhoC T19N (green), and the fitted mixture of RhoC (red).

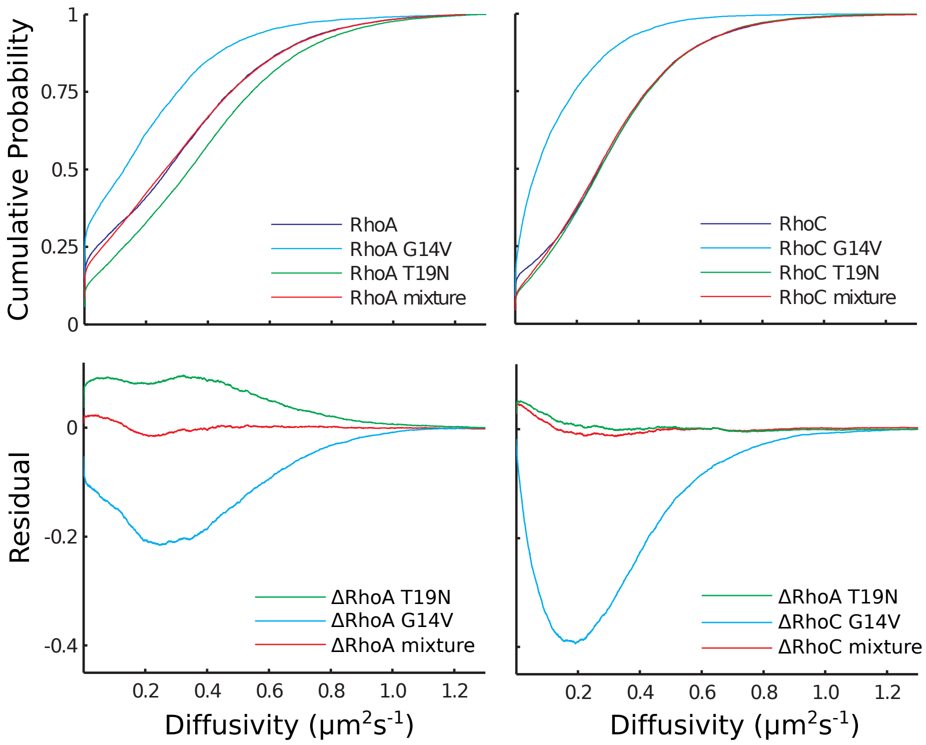

Supplement: S8 Text — (PDF) [file pcbi.1004297.s024.pdf]
