# Supplementary material for: Extracting Diffusive States of Rho GTPase in Live Cells: Towards In Vivo Biochemistry
Source: PLoS Comput Biol. 2015 Oct 29;11(10):e1004297. doi: 10.1371/journal.pcbi.1004297 (PMC4626024; doi:10.1371/journal.pcbi.1004297)
Supplement: S9 Text — (PDF) [file pcbi.1004297.s025.pdf]

S9 Text: Supporting information references

67. Einstein A (1956) Investigations on the Theory of the Brownian Movement. Courier Dover Publications.

68. Metzler R, Jeon JH (2012) The role of ergodicity in anomalous stochastic processes: analysis of single-particle trajectories. Physica Scripta 86: 058510.

69. Jeon JH, Metzler R (2010) Analysis of short subdiffusive time series: scatter of the time-averaged mean-squared displacement. Journal of Physics A: Mathematical and Theoretical 43: 252001.

70. Voisinne G, Alexandrou A, Masson JB (2010) Quantifying biomolecule diffusivity using an optimal Bayesian method. Biophysical Journal 98: 596–605.

71. Bel G, Barkai E (2005) Weak ergodicity breaking in the continuous-time random walk. Physical Review Letters 94: 240602.
